# Development of background-free tame fluorescent probes for intracellular live cell imaging

**DOI:** 10.1038/ncomms11964

**Published:** 2016-06-20

**Authors:** Samira Husen Alamudi, Rudrakanta Satapathy, Jihyo Kim, Dongdong Su, Haiyan Ren, Rajkumar Das, Lingna Hu, Enrique Alvarado-Martínez, Jung Yeol Lee, Christian Hoppmann, Eduardo Peña-Cabrera, Hyung-Ho Ha, Hee-Sung Park, Lei Wang, Young-Tae Chang

**Affiliations:** 1 Department of Chemistry & Med Chem Program, National University of Singapore, Singapore 117543, Singapore; 2 Department of Chemistry, Korea Advanced Institute of Science and Technology, Daejeon 305701, Republic of Korea; 3 Laboratory of Bioimaging Probe Development, Singapore Bioimaging Consortium, Agency for Science, Technology and Research, Singapore 138667, Singapore; 4 Department of Pharmaceutical Chemistry and the Cardiovascular Research Institute, University of California San Francisco, California 94158, USA; 5 Departamento de Química, Universidad de Guanajuato, Guanajuato 36050, Mexico; 6 College of Pharmacy and Research Institute of Life and Pharmaceutical Sciences, Sunchon National University, Sunchon 540950, Republic of Korea

## Abstract

Fluorescence labelling of an intracellular biomolecule in native living cells is a powerful strategy to achieve in-depth understanding of the biomolecule's roles and functions. Besides being nontoxic and specific, desirable labelling probes should be highly cell permeable without nonspecific interactions with other cellular components to warrant high signal-to-noise ratio. While it is critical, rational design for such probes is tricky. Here we report the first predictive model for cell permeable background-free probe development through optimized lipophilicity, water solubility and charged van der Waals surface area. The model was developed by utilizing high-throughput screening in combination with cheminformatics. We demonstrate its reliability by developing CO-1 and AzG-1, a cyclooctyne- and azide-containing BODIPY probe, respectively, which specifically label intracellular target organelles and engineered proteins with minimum background. The results provide an efficient strategy for development of background-free probes, referred to as ‘tame' probes, and novel tools for live cell intracellular imaging.

## Abstract (web summary)

The success of a fluorescent dye as a molecular probe to monitor the intracellular activity of biomolecules depends on its physicochemical characteristics. Here, the authors use a predictive model to identify key features that allow them to design cell permeable, background-free fluorescent probes.

## Graphical abstract

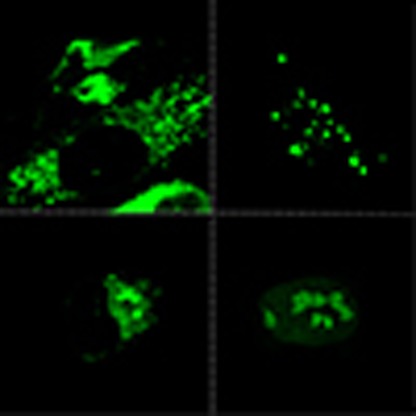

Fluorescent probes have emerged as one of the widely-used tools for investigating biological systems, thanks to its sensitivity and technical simplicity. Labelling a biomolecule, particularly protein, with a functional small probe in living cell is a popular approach to obtain greater knowledge of its biological roles and functions. However, labelling a specific intracellular biomolecule is a challenging task because cells are complex, highly organized and composed of various biological events. As a result, most labelling probes reported thus far leave fluorescence background due to nonspecific interactions with other cellular components^1^,^2^,^3^,^4^,^5^. In addition, probes delivery into live cells can also be demanding as some probes are not able to cross plasma membranes in native condition, thereby limiting their utility for intracellular labelling.

Despite their intricacies, physicochemical properties of probes can be defined numerically through their structural information, so-called descriptors. Descriptors have long been exploited in computational quantitative structure activity relationship study, for instance, to predict aqueous solubility and permeability of drugs^6^,^7^, binding affinities of antitumor drugs^8^,^9^ and also cellular uptake and localization of fluorescent probes^10^,^11^.

Rational design of cell permeable background-free ‘tame' probes is tricky and generally involves a long process of trial-and-errors. Here we utilize probes' descriptors for statistics-based quantitative structure activity relationship study to develop a model that can serve as a guideline to design such ideal ‘tame' probes by predicting probes' cellular uptake and retention through their structural parameters. The model identifies three features: SlogP (lipophilicity), logS (water solubility) and Q_VSA_FNEG (negatively charged van der Waals surface area), as key factors influencing cell permeability and nonspecific intracellular retention of small fluorescent probes. This new model is then applied to design cyclooctyne- and azide-containing BODIPY ‘tame' probes that can be utilized for labelling various intracellular organelles and engineered proteins via copper-free strain-promoted alkyne-azide cycloaddition (SPAAC).

## Results

### Cellular retention and efflux characteristics of the probes

**Figure 1:**
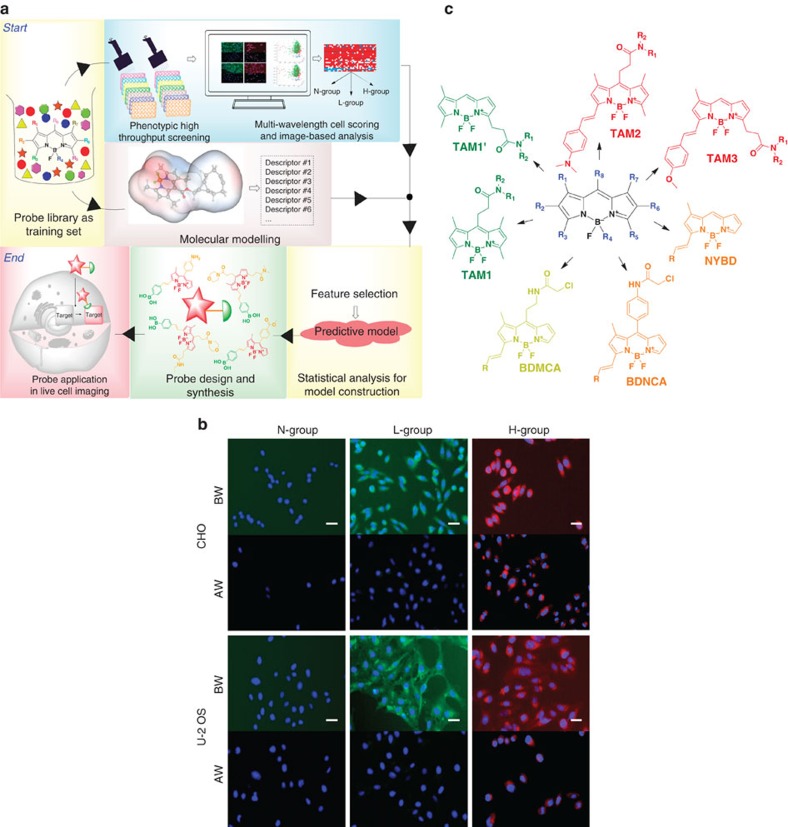
Experimental set and probes' cellular responses. (**a**) Flowchart of the overall experimental strategy. (**b**) Representative cellular responses in CHO and U-2 OS. Cells were stained with probes at 1 μM final concentration. The overlayed images of Hoechst33324 (blue) and probe signals (according to emission wavelength of the probes) show three phenotypic groups: cell-impermeable group where probes are unable to stain the cell (N-group); cell permeable with low nonspecific binding group (L-group); and cell permeable with high nonspecific binding group (H-group). BW and AW indicate image before and after washing, respectively. Probes signal is significantly decreased after washing in L-group, while is retained in H-group. Scale bar, 10 μm (green, red and blue signals are from FITC, Texas Red and DAPI channel, respectively). (**c**) Core structures of the BODIPY libraries used in training set.

To expedite an efficient strategy for ‘tame' probe development, we set up a system to predict cellular properties of fluorescent probes via phenotypic high-throughput screening (HTS), cheminformatics and statistical analysis (Fig. 1a). As the first step, we conducted phenotypic HTS to investigate cell permeability and nonspecific binding properties of the probes in a training set. We tested about 2,085 probes consisted of a wide range photophysical properties from numerous fluorophore cores such as BODIPY^12^,^13^,^14^,^15^, xanthone^16^,^17^, rosamine^18^, cyanine^19^,^20^ and ACEMAN^21^. Their cellular influx and efflux profiles were examined in two types of mammalian cells, U-2 OS and CHO. These cell lines were chosen due to their flexibility in genetic manipulation, adaptability to various culture conditions and versatility in wide biomedical research such as gene expression, toxicity screening, recombinant protein and live cell imaging. Each of the probes was added into live cells and cell image was taken after 30 min by ImageXpress Micro cellular imaging system (Molecular Device). The cells were then washed with fresh growth media, and after another 10 min, a second image was taken to check the completeness of probe washing out of the cell. The first and second imaging facilitated influx (before washing, BW) and efflux (after washing, AW) measurements, respectively (Supplementary Fig. 1).

Three major phenotypic groups were observed: (i) N-group, non-stain group where probes are unable to pass through the cell membrane, (ii) L-group, low nonspecific binding group where probes enter the cell and leave after washing, and (iii) H-group, high nonspecific binding group where probes pass through cell membrane and retain inside after washing (Fig. 1b). L-group signifies the desired feature of the properly tamed probes as the influx and efflux occur rapidly with minimal background. We observed that probes in L-group came from only the BODIPY family. Probes from the rosamine and cyanine family strongly bound to mitochondria, hence mainly in H-group, whereas most xanthone and ACEMAN probes were not able to pass through cellular membrane. Therefore, we focus only on BODIPY family (920 probes) as a model system for further analysis (Fig. 1c). BODIPY can be an ideal fluorophore as it has an electrically neutral core and superior optical properties in terms of high quantum yield, tunable fluorescence characteristics, high photostability and narrow emission bandwidth^22^.

### Retaining ratio for automated cellular phenotypic grouping

**Figure 2:**
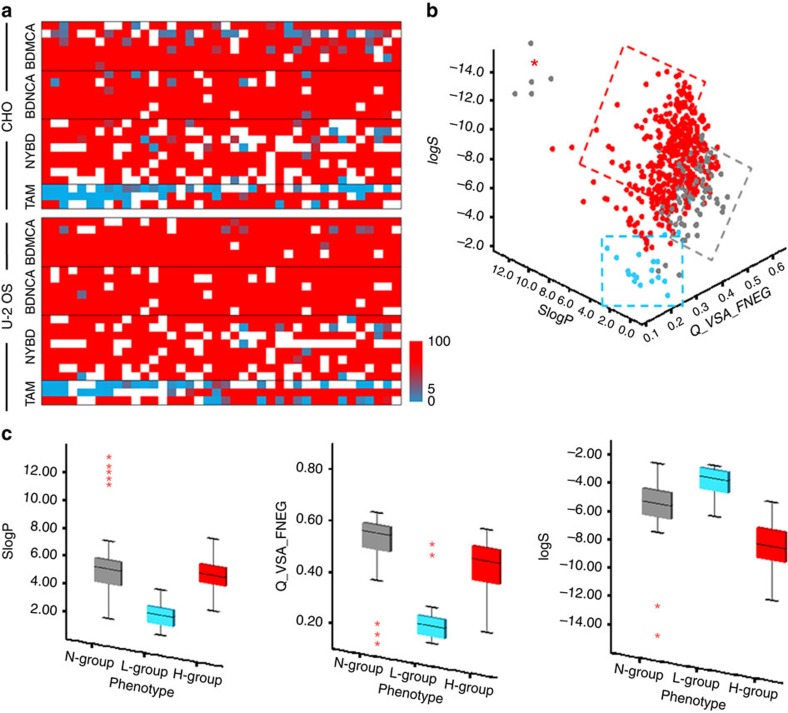
Predictive model. (**a**) Heat maps of RR for each library in U-2 OS and CHO cell lines. White colour represents probes in N-group. The red and torquise colour represents high and low RR, respectively. (**b**) Three dimensional scatter plot for three key descriptors: SlogP, Q_VSA_FNEG and logS. Grey, turquoise and red dotted box represents area containing mostly probes from N-group, L-group and H-group, respectively. (**c**) Preferred criteria for ‘tame' probes. Descriptor values in each group are presented as box plots with medians, quartiles, and interquartile range. The whiskers represent the minimum and maximum scores, and the red asterisks show extreme scores.

To measure the influx and efflux of the probes, an automated quantitative intensity-based image analysis was then performed by Multi-Wavelength Cell Scoring analysis using MetaXpress High Content Image software. Retaining ratio (RR, per cent ratio of the average intensity of AW image over BW image) was computed to classify probes into the three phenotypic groups. For each cell in the image, the nuclei and cell region were segmented to provide approximate cellular boundaries. Fluorescence intensity was then quantified on a cell-by-cell basis by the following steps: (i) selecting the image of interest, (2) specifying the width of the stained cell area, (3) adjusting the intensity above local background for detection specificity, and (4) calculating fluorescence intensity with specified reporting parameters. Average fluorescence intensity for each BW and AW images was calculated for each probes and cell lines, and was then used as input in RR calculation (Supplementary Figs 2 and 3). RR heat map showed that about 88% of the probes behaved similarly in both U-2 OS and CHO, implying that these two cell lines are sufficient to serve as a proxy for phenotypic screening (Fig. 2a, Supplementary Fig. 4). From this 88% population, a majority of them (81%) were in H-group, 16% of the probes were observed to be cell-impermeable, and 3% were ‘tame' probes (Supplementary Fig. 5).

### Molecular descriptors can predict ‘tame' probes' properties

Next, we investigated the structural properties of the training set. Active three-dimensional (3D) conformation of the libraries was set through energy minimization by Hamiltonian AM1 method using Molecular Operating Environment (MOE) software. A total of 327 molecular descriptors for each probe were then generated (Supplementary Table 1). Although hundreds of descriptors were generated, not all are relevant to the study; therefore, we cleaned up the data by removing redundant descriptors. Independency between descriptors was evaluated by checking their degree of noncorrelation using SPSS v.13.0.1 software package (SPSS, Inc., Chicago, IL). A number of redundant descriptors were then exempted, such as: highly correlated descriptors (for example, Q_VSA_FHYD and Q_VSA_FPOL, these descriptors have a perfect negative correlation as shown by the independency test, hence one of them can be exempted to eliminate redundancy), duplicated descriptors (for example, PC+ and RPC+; PC− and RPC−), insignificant descriptors (for example, rsynth, which describe the easiness of the synthesis process) and uninterpretable descriptors (for example, BCUT_PEOE_0, BCUT_PEOE_1, BCUT_PEOE_2, BCUT_PEOE_3). This elimination resulted in 39 descriptors for further investigation (Supplementary Table 2).

To identify the important descriptors responsible for probe phenotypic grouping, we carried out significance test-based feature selection (STFS). In STFS, the statistical significant differences of descriptors among the three phenotypic groups were calculated to evaluate the pattern recognition ability of individual descriptors. We firstly carried out a normality test to check if our data set follows a normal distribution so as to apply proper statistical methods. Formal statistical Shapiro–Wilk test was used to check data normality. Conceptually, Shapiro–Wilk test involves an arrangement of the sample values and measurement against expected means and/or variances. The measure of central tendency is important: if data distribution is normal, then both mean and median can be measured to be presented; otherwise the median should be more appropriate. Results in Supplementary Table 3 show that nonparametric type STFS analysis was suitable for our database.^23^,^24^,^25^

Next, the nonparametric Kruskal–Wallis test followed by Eta-squared test was conducted to statistically evaluate whether there is a significant difference between descriptors' median in the three phenotypic groups (descriptors were considered to have a statistically significant difference if *P<*0.01). Kruskal–Wallis significance level was estimated on the capability of individual descriptor to form a distinct separable pattern, while Eta-squared test was used to measure the effectiveness of the descriptors intervention relative to phenotypic grouping. Our analysis suggests that lipophilicity, water solubility, surface area properties and size^26^ of the probes significantly contributed to their cellular permeability and nonspecific intracellular retention (Supplementary Fig. 6, Supplementary Table 4). To make an interpretable and uncomplicated model, descriptors with large effect in probes' phenotypic grouping (*η*^2^≥0.1) were selected and analysed by using principal component analysis method to further portray the patterns and highlight the relations of those selected descriptors.

The first three principal components account for 98.0% of the data set variance (Supplementary Table 5). Examination of the component loadings shows that descriptors are arranged into three clusters: lipophilicity (SlogP, logPo/w), water solubility (logS), and charged-surface area properties (Q_VSA_NEG and Q_VSA_FNEG), indicating that these three cluster properties are the major determinant for cellular behaviour of the probes (Supplementary Figs 7 and 8). Interestingly, principal component analysis (PCA) loading plot indicated that three descriptors (Q_VSA_FNEG (*χ*^2^(2)=88.823, *P*=0.000, *η*^2^=0.110), logS (*χ*^2^(2)=110.411, *P*=0.000, *η*^2^=0.137), and SlogP (*χ*^2^(2)=102.268, *P*=0.000, *η*^2^=0.1272)) are sufficient to summarize the data with minimal loss of information (Fig. 2b). Our results thus suggest that criteria for the ideal probes are adequately lipophilic (SlogP=1 to 4), moderately negatively charged on its surface (Q_VSA_FNEG=0.15 to 0.35) and highly soluble in water (logS=−2 to −6; Fig. 2c, Supplementary Fig. 9).

### Validation set for the interpretable model

The ultimate goal of a modelling exercise is not only to fit a model into a data set, but importantly to also provide a clear guide to design and synthesize molecules with desired properties. As a proof of concept, we applied the model to an extended set of our in-house BODIPY library (EP library) consists of 107 various structures (Supplementary Fig. 10). On the basis of their descriptors value, the model predicted that nine of the EP probes are in L-group (‘tame' probes). Cell experiment showed that eight of the nine probes are indeed in L-group, which is corresponding to a prediction accuracy rate of 89% (Supplementary Table 6).

It is interesting to note that BODIPY is known to be sticky/hydrophobic and usually leaves high background in the cells. To tame BODIPY into background-free probe while keeping its cellular permeability, we synthesized boronic acid-bearing BODIPY probes, which have a tremendous importance in chemical biology field, by following our model guidelines. Based on their SlogP, Q_VSA_FNEG and logS values, BOR-1H and BOR-2H were predicted to be in H-group while BOR-1 and BOR-2 were in L-group (Supplementary Table 7, Supplementary Fig. 11). We found that the predicted phenotypic groups for these probes are in agreement with the observed cell experimental results, hence validating our predictive model for background-free probes (Supplementary Table 8, Supplementary Fig. 12).

### ‘Tame' probes for intracellular labelling in living cells

To further demonstrate the model and its applications, we synthesized BCN alcohol-containing BODIPY probes, CO-1 and CO-1H, to fit the L-group and H-group, respectively, by varying substituents at different positions of the BODIPY core for SPAAC-based labelling. Their absorbance and emission are similar to GFP, thus enabling their visualization on most standard fluorescence microscope (Supplementary Fig. 13). Fascinatingly, cellular retention and efflux study showed the phenotypic grouping of CO-1 and CO-1H as predicted (Supplementary Fig. 14, Supplementary Table 9).

**Figure 3:**
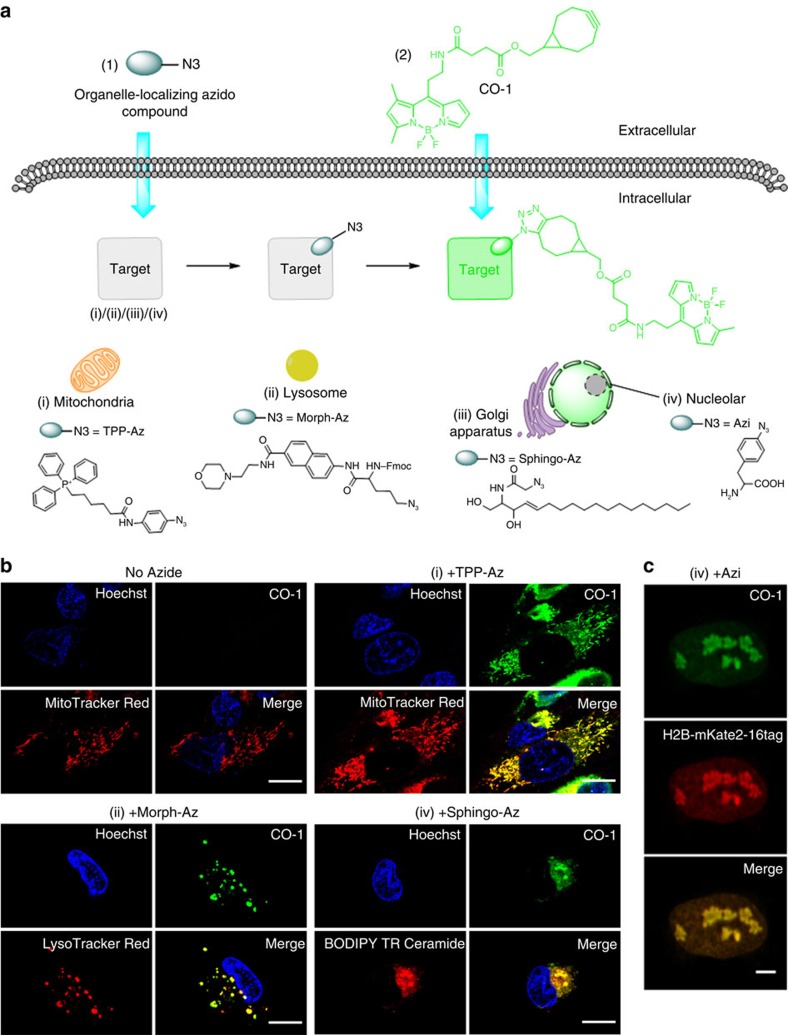
Live cell imaging with CO-1. (**a**) Chemical structures and schematic illustration of the covalent labelling of azide-tagged organelles using CO-1 in live cells. (**b**) Fluorescence imaging of mitochondria, lysosome and golgi apparatus in U-2 OS cells labelled with CO-1. Cells pre-treated with TPP-Az, Morph-Az or Sphingo-Az were incubated with 2 μM CO-1 in growth media at 37 °C for 1 h and followed by counterstaining with organelle trackers. Scale bar, 15 μm. (**c**) Fluorescence imaging of histone H2B in live U-2 OS. U-2 OS cells were co-transfected with plasmids pIre-Azi3 and pmH2B-6-mKate2-16tag to incorporate UAA Azi into H2B-mKate2 at site 16. After Azi incorporation, cells were labelled with 10 μM CO-1 for 90 min at 37 °C. Cells were then washed and imaged. The mKate2 (red) signals were detected in cell nuclei only, and colocalized with the CO-1 (green) signals in the merged image. Scale bar, 5 μm.

Next, we investigated intracellular imaging in live cells using CO-1. We synthesized various azide-bearing reporters: TPP-Az, a triphenylphosphonium analogue which accumulates in mitochondria; Sphingo-Az, a ceramide analogue to target golgi apparatus; and Morph-Az, an azide derivative with morpholine moiety as a directing group for lysosome (Fig. 3a). SPAAC covalent labelling reactions between CO-1 and all azide reporters *in vitro* were clean and rapid. We anticipated that the cell permeable CO-1 is capable of labelling any azide reporters effectively, regardless the reporters' cellular localization. We added 2 μM CO-1 to the growth media of U-2 OS cells pre-treated with TPP-Az, Sphingo-Az or Morph-Az separately. Confocal fluorescence images were then taken after probe washing. As shown in Fig. 3b, CO-1 clearly labelled the azide-tagged intracellular organelles in live cells, and CO-1 signal was nicely colocalized with respective organelle probe trackers. Clean fluorescence background was observed due to washable property of the unreacted CO-1. In contrast, when CO-1H was added to the cells pre-treated with TPP-Az, Sphingo-Az or Morph-Az, high-fluorescence background from CO-1H interfered with the observation of the intracellular organelles (Supplementary Fig. 15).

We further demonstrated the utility of CO-1 for visualization of engineered protein with an example of human histone H2B, which localizes at the nucleolus. The red fluorescent protein mKate2 was fused to the C terminus of human histone H2B as a red fluorescent marker. Using the *in vivo* unnatural amino acid (UAA) technology,^27^,^28^ an azide-bearing UAA, *p*-azido-L-phenylalanine (Azi), was site-specifically incorporated at site 16 of mKate2 of the fusion protein H2B-mKate2, which was expressed in live U-2 OS cells. The cells were labelled with CO-1, washed to remove unreacted CO-1, and then imaged with laser scanning confocal microscopy. To our delight, CO-1 was not only able to label cytosolic organelles, but also to pass through nuclear membrane. Clear CO-1 signal was detected in the live cell nucleolus out of low background, and was highly colocalized with mKate2 signals, suggesting specific labelling of histone H2B (Fig. 3c, Supplementary Fig. 16). Moreover, CO-1 exhibited superior photostability under intensive radiation of strong light source in aqueous solution (Supplementary Fig. 17) and was found to be nontoxic and did not interfere with cell proliferation (Supplementary Fig. 18), making CO-1 suitable for prolonged time lapse imaging.

**Figure 4:**
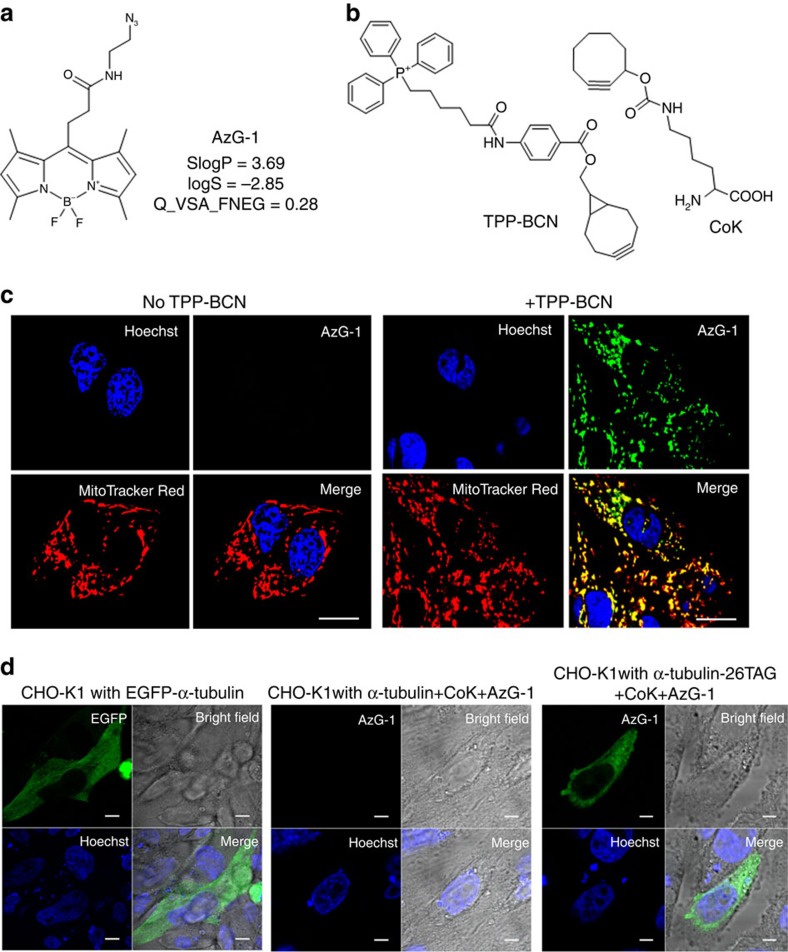
Live cell imaging with AzG-1 (**a**) Chemical structures of azide-bearing probe AzG-1 and its descriptor values. (**b**) Structure of triphenylphosphonium analogues TPP-BCN and cyclooctyne-containing unnatural amino acid, CoK. (**c**) Fluorescence imaging of mitochondria in U-2 OS cells labelled with AzG-1. Cells were incubated without (left side) and with (right side) TPP-BCN and then labelled with 10 μM AzG-1 for 2 h followed by counterstaining with mitochondria marker. Scale bar, 10 μm. (**d**) Fluorescence imaging of α-tubulin in live CHO-K1 cells. CHO-K1 cells were co-transfected with plasmids pCoKRS-tRNA and pTub-26TAG to incorporate CoK into α-tubulin at position 26. After incorporation of CoK, cells were labelled with 10 μM AzG-1 for 2 h at 37 °C. CHO-K1 cells were also transfected with plasmid pTubwt to express wild type α-tubulin and treated with CoK and AzG-1 in the same way. CHO-K1 cells transfected with plasmid pEGFP-Tubwt expressing EGFP-fused wild type α-tubulin was used as a control. Live cell images show that AzG-1 labelled mitochondria (**c**) and is conjugated specifically with CoK-bearing α-tubulin (**d**). Scale bar, 10 μm.

Excited with the results, we further challenged the model and developed another ‘tame' azide-bearing probe, AzG-1, to label molecules in mitochondria and the fancy tubulin protein in living cells (Figs 4a,b). To target mitochondria, U-2 OS cells were pre-treated with TPP-BCN (an analogue of triphenylphosphonium) before labelled with 10 μM AzG-1. For tubulin labelling, a cyclooctyne-containing UAA, N-ɛ-(cyclooct-2-yn-1-yloxy)carbonyl)L-lysine (CoK) was selected for incorporating into tubulin and reacting with AzG-1 via SPAAC.^29^ CHO-K1 cells were co-transfected with plasmids pCoKRS-tRNA and pTub-26TAG to incorporate CoK into α-tubulin at position 26. Site-specific incorporation of CoK using an orthogonal tRNA^Pyl^/CoKRS pair was confirmed via western blot analysis (Supplementary Figs. 19 and 20). After incorporation of CoK, cells were then labelled with 10 μM AzG-1 at 37 °C. CHO-K1 cells transfected with plasmid pEGFP-Tubwt expressing EGFP-fused wild-type α-tubulin was used as a control. Confocal fluorescence images were then taken AW of unreacted AzG-1. Cell images clearly show that AzG-1 specifically labelled mitochondria (Fig. 4c) and was also conjugated specifically with CoK-bearing α-tubulin (Fig. 4d, Supplementary Fig. 21).

## Discussion

The combination of cheminformatics and HTS of in-house probe library yielded a novel predictive model to guide the development of cell permeable background-free ‘tame' probes for intracellular imaging in live cells. Our systematic analysis showed that cellular behaviour of fluorescent probes can be predicted from their photophysical properties; more importantly, we found that lipophilicity, charged van der Waals surface area and water solubility are significant determinants for probes' cellular permeability and nonspecific binding in living cells. These descriptors may account for not only polar noncovalent interactions between probes and phosphate group of the plasma membranes during cellular intake, but also cumulative charge interactions with macromolecules inside the cells.

Our result supports previous studies that hypothesized hydrophobicity and surface charge may correlate with nonspecific binding properties of fluorescent probes^10^,^30^,^31^. In addition, water solubility also plays a key factor in slow cellular efflux of the probes. Our observation in cellular retention and efflux study showed that probes were stably solubilized when added to the cells, indicating that extracellular insoluble formation of the probes is unlikely. However, after the probes were incubated in cells for 30 min, the formation of intracellular insoluble or precipitate-like form which is hardly washed out of cells was observed from some of the probes in H-group. It suggests that intracellular precipitation or aggregation due to the low water solubility may be one of the mechanisms behind the slow cellular efflux and high retention of probes in H-group^32^,^33^,^34^.

We utilized this model strategy to synthesize probes for copper-free SPAAC in living cells^3^,^35^. Early study on SPAAC reported nonpermeant cyclooctyne probes for imaging extracellular surface glycans^36^,^37^,^38^. Following that, an attempt was also made to develop cyclooctyne probe for targeting intracellular biomolecules^4^. However, this probe still suffers from high nonspecific binding. Here we show that through structure optimization on the three interpretable descriptors (lipophilicity, charged van der Waals surface area and water solubility), we are able to modulate it to meet the demands of designing smart ‘tame' probes.

In summary, our predictive model provides a powerful strategy to efficiently develop background-free BODIPY probes for intracellular labelling in native living cells. We successfully demonstrated the robustness of this strategy by developing novel labelling tools, CO-1 and AzG-1, which are not only highly cell permeable and washable, but also exhibit a remarkable capability of labelling various intracellular organelles and engineered proteins. The strategy should allow the exploration of dynamic processes (for example, cellular localization, expression level and biological activities) of newly synthesized intracellular biomolecules in their native cellular environment.

## Methods

### Probes' synthesis and characterization

Details of synthesis and characterization of the probes used in this study can be found in the Supplementary Methods. General synthetic schemes and structures of the BODIPY libraries used in training set can be seen in Supplementary Figs 22 and 23 and Supplementary Tables 10 and 11. For NMR spectra of CO-1, AzG-1 and CO-1H see Supplementary Figs 24–26.

### Cellular retention and efflux characteristics test

U-2 OS, a human osteosarcoma cell line, and CHO, a Chinese hamster ovary cell line, (from ATCC) were cultured in Dulbecco's modified eagle's medium (DMEM) (Invitrogen, CA, USA) supplemented with fetal bovine serum (FBS) (10%) and penicillin–streptomycin (1%). Materials used in the cell culture were purchased from Invitrogen. U-2 OS and CHO cells were seeded onto 96-well plate in growth media at 37 °C in the presence of 5% CO_2_ and were then allowed to attach and grow to 70–80% confluence. Library probes were dissolved in dimethylsulfoxide (DMSO) to make the 1 mM solution, and stored in −20 °C. Before HTS experiment, the growth media was aspirated and replaced by 200 μl fresh growth media containing probes in final concentration of 1 μM and nuclei dye Hoechst 33342. Plates were incubated for 30 min at 37 °C then were imaged using ImageXpress Micro cellular imaging system (Molecular Device) with × 10 objective lens. Immediately after image acquisition, the cells were washed with fresh growth media, and transferred back to a 37 °C cell incubator for further incubation. After 10 min, cells were again imaged (AW image). The first imaging step allowed influx measurement of BW image , while the second imaging step allowed out-flux measurement of AW image. Images of two regions per well were acquired with DAPI, FITC, TRITC or Texas Red filter sets according to probes' excitation and emission wavelength. Experiment was performed in duplicate. Cellular retention and efflux characteristics test for other synthesized probes were also done by using the same protocol.

### RR calculation

RR was computed to quantitatively estimate probes retained inside the cells AW. RR was calculated by:


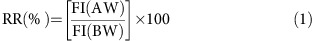


FI_(AW)_ is the average fluorescence intensity AW and FI_(BW)_ is the average fluorescence intensity BW. RR is a value ranging from 0 to 100, where higher the value, higher the nonspecific binding of the probes. Background correction was applied to each image to compensate the uneven background intensity, thus improving image segmentation and reducing calculation bias. Probes in which BW images having FI_(BW)_ ≤200 were classified as N-group. Probes (FI_(BW)_ >200) with rapid efflux were observed to have a RR of <5%, and thus were classified into L-group, while those with RR >5% were classified into H-group.

### Molecular descriptor for library training set

Chemical Computing Group (CCG) MOE 2011 software package was used for generating 3D structure and molecular descriptors for each probe. Hydrogens and lone-pair electrons were adjusted as required. Partial charge was set and protonation state was corrected for each structure. The active 3D conformation of the libraries was then acquired through energy minimization by Hamiltonian AM1 method. A total of 327 molecular descriptors were generated including topological, molecular connectivity, electrotopological geometric and quantum chemical descriptors.

### Significance test-based feature selection

STFS analysis was done using SPSS v.13.0.1 software package (SPSS Inc., Chicago, IL). *η*^2^-test was calculated by:


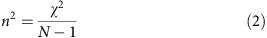


where *η*^2^ is eta squared, *χ*^2^ is chi square value and *N* is sample size. PCA was performed using the same software. In PCA, each of the descriptors and probes were processed and their factors of correlation were extracted.

### Cell maintenance and preparation for cell labelling

The reagents Hoechst 33342 (1:5000, H1399), MitoTracker Deep Red FM (referred to as MitoTracker Red), BODIPY TR ceramide and LysoTracker Red were purchased from Life Technologies (Carlsbad, CA, USA). A stock solution of CO-1, CO-1H, TPP-Az, Morph-Az, Hoechst 33342, MitoTracker Red, and LysoTracker Red were first prepared in DMSO. The fluorescence excitation and emission spectra were measured using a SpectraMax M2 plate reader (Molecular devices Corp, USA). U-2 OS cells were cultured at 37 °C with 5% CO_2_ in DMEM supplemented with 10% (v/v) FBS, penicillin (100 units per ml) and streptomycin (100 mg ml^−1^). Cells were passaged two to three times a week, seeded at a density of 75,000 cells per cm into 35-mm glass bottom dishes, and grown in DMEM media overnight before labelling.

### Sphingo-Az stock solution preparation

BODIPY TR ceramide were prepared according to manufacturer protocol. Sphingo-Az was prepared as a form of complex with BSA similar to the preparation of BODIPY TR ceramide. Solid Sphingo-Az was dissolved in chloroform:ethanol (19:1 v/v) to give 50 μl of 1 mM stock solution. The stock solution was dried and redissolved in 200 μl 100% ethanol. This solution was then added to 10 ml of HBBS/BSA solution (HBSS+10 mM HEPES pH 7.4+0.34 mg ml^−1^of defatted BSA) on a vortex mixer to give 5 μM Sphingo-Az/5 μM BSA stock solution. This solution can then be stored at −20 °C.

### Mitochondria imaging in live cells

U-2 OS cells were treated with 5 μM TPP-Az, in culture media for 2 h at 37 °C. After incubation, cells were washed and added with 2 μM CO-1 probe. Labelling was allowed to proceed for 1 h at 37 °C in the incubator chamber. Following incubation, cells were treated with nuclei dye Hoechst 33342 (1 μg μl^−1^) and MitoTracker Red (1 μM). After counterstaining, cells were washed three times with growth media and imaged using Nikon A1R+ confocal laser microscope system.

### Lysosome imaging in live cells

U-2 OS cells were treated with 5 μM Morph-Az, in culture media for 1 h at 37 °C. After incubation, cells were washed and added with 2 μM CO-1 probe then proceed for 1 h incubation at 37 °C. Following incubation, cells were treated with nuclei dye Hoechst 33342 (1 μg μl^−1^) and LysoTracker Red (1 μM). After counterstaining, cells were washed three times with growth media and imaged using Nikon A1R+ confocal laser microscope system.

### Golgi apparatus imaging in live cells

U-2 OS cells were treated with 5 μM Sphingo-Az/BSA and 5 μM BODIPY TR ceramide/BSA in HBSS/HEPES for 30 min at 4 °C. Afterwards, cells were washed with cold growth media and further incubated for 30 min at 37 °C. After incubation, cells were washed and incubated with 2 μM CO-1 in culture media for 1 h at 37 °C. Following incubation, cells were treated with nuclei dye Hoechst 33342 (1 μg μl^−1^). After counterstaining, cells were washed three times with growth media and imaged using Nikon A1R+ confocal laser microscope system.

### A1R+ confocal microscopes

Confocal imaging experiments were performed on an inverted Nikon A1R+ confocal laser microscope system using 562/672/405 nm lasers with Plan Apo TIRF 100X DiC oil H H2 objectives (Nikon Instruments, Inc., Japan). Image processing and overlay analysis were performed using NIS Elements 3.10 software (Nikon Instruments, Inc.).

### Molecular cloning for histone H2B labelling

Plasmids were cloned in DH10β *Escherichia coli* strain and confirmed by DNA sequencing. PfuTurbo (Stratagene, La Jolla, CA) was used for QuikChange reactions and Phusion (Finnzymes, Finland) for gene amplification. Primers were synthesized by ValueGene (San Diego, CA), and restriction enzymes were purchased from New England Biolabs (Ipswich, MA). Wild-type mKate2 was cloned into pmTagRFP-T-H2B-6 plasmid using the *Age*I and *Not*I sites. The amber stop codon UAG was introduced at site 16 of mKate2 to make the plasmid pmH2B-6-mKate2-16tag.

### Newly synthesized histone H2B imaging in live cells

U-2 OS cells were transfected with 1 μg DNA of each plasmid using Lipofectamine2000 in 35 mm glass bottom dishes according to the manufacturer protocol. Azi (purchased from Bachem) was added 1 h before transfection at a final concentration of 0.5 mM. Plasmid pIre-Azi3 (expressing the orthogonal amber suppressor transfer RNA (tRNA) and the E2AziRS specific for Azi) was co-transfected with plasmid pmH2B-6-mKate2-16tag in a 1:1 molar ratio. After 24 h, cells were washed three times with culture medium (DMEM with 10% FBS) (15 min each time). Growth media containing 10 μM CO-1 was then added to the dishes, and cells were incubated at 37 °C for 90 min followed by 4 times washing with growth media (30 min each time). Laser scanning confocal microscopy (Zeiss LSM 700) was used for imaging. The same conditions and settings were used for imaging control and experimental cells.

### Plasmid construction for site-specific CoK incorporation

Cyclooctyne-containing UAA, N-ɛ-(cyclooct-2-yn-1-yloxy)carbonyl)L-lysine (CoK) was selected for copper-free labelling of proteins to avoid the use of cytotoxic metals. To this end, cyclooctynyl lysine, CoK was synthesized according to the previous report^29^. For genetic incorporation of CoK, an engineered orthogonal tRNA^Pyl^/CoKRS pair was constructed as reported before^29^. To express proteins installed with CoK at a designated position in mammalian cells, plasmids expressing the tRNA^Pyl^/CoKRS pair and target protein(s) were constructed in the following way. CoKRS carrying C-terminal haemaglutinin (HA) tag is expressed under the control of EF-1α promoter (derived from pEFIRES vector, Clonetech) and tRNA under the control of human U6 promoter and the CMV enhancer. CoKRS and tRNA expression cassettes were cloned using *Kpn*I*/Not*I, and *BamH*I*/Asc*I sites of pCDNA3 vector (Invitrogen) respectively, generating plasmid pCoKRS-tRNA. For live cell labelling experiments, α-tubulin was used as a model protein. An amber stop codon was inserted into position 26 of α-tubulin and the resulting tubulin-26TAG under the control of EF-1α promoter was cloned between *Kpn*I and *Not*I of pCDNA3, generating plasmid pTub-26TAG. Plasmids pTubwt and pEGFP-Tubwt carrying α-tubulin wild type and EGFP-fused α-tubulin, respectively, were also constructed in a similar way for control experiments. To validate genetic incorporation of CoK using tRNA^pyl^/CoKRS pair, plasmids pEGFP-Tub-26TAG (carrying EGFP-fused α-tubulin with C-terminal FLAG tag and stop codon TAG at 26) and pEGFP-39TAG (carrying EGFP with stop codon TAG at 39) were constructed for Western blotting and fluorescence microcopy analysis.

### Protein labelling on live cells using CoK

CHO-K1 or HeLa cells were grown in DMEM-low glucose 1 g l^−1^ (Gibco) supplemented with 10% FBS (Gibco) in eight-well plate (ibid). At 70∼80% confluency, cells were transfected using 4 μl iN-fect *in vitro* transfection reagent with 100 ng pCoKRS-tRNA and 900 ng pTub-26TAG or with 900 ng pTubwt. After 6 h of incubation, media was replaced with fresh media (DMEM-low glucose medium with 10% FBS, 1% Pen-Strep, 2 Mm L-glutamate) containing 0.25 Mm CoK. After an additional 36 h, media was exchanged with normal growth media without CoK and cells were incubated for 2 h and washed (four times) to remove remaining CoK. Next, growth media containing 1 μM AzG-1 was added to the plates, and cells were incubated for 2 h with four times of washing (30 min each time) using growth media. High-resolution images of live cells were obtained using a Zeiss LSM510 META laser scanning confocal microscope.

### Western blotting

To demonstrate site-specific incorporation of CoK into α-tubulin, HEK293T cells were co-transfected with plasmids pCokRS-tRNA and pEGFP-Tub-26TAG at 80–90% confluency and cultured in the presence or absence of 0.5 mM CoK for 36 h. The cells were harvested and subjected to Western blot analysis using anti-HA antibody (Abcam) and anti-FLAG antibody (Agilent) for the detection of HA-tagged CoKRS expression and FLAG-tagged EGFP-α-tubulin expression, respectively.

### Cell viability assay

Cell viability was assessed using MTS assay kit (Promega). 1 × 10^5^ U-2 OS cells were seeded on 10 mm culture dishes; cells were stained with or without CO-1 (0–10 M) for 1–16 h. After incubation, the MTS reagent was added and incubated for 4 h at 37 °C. Absorbance was determined at 490 nm using SpectraMax M2 plate reader.

### Photostability measurements

10 μM of CO-1 solution in PBS buffer (pH 7.4) containing 1% DMSO were placed in a 96-well plates. Fluorescence measurement were recorded every 30 s interval for a total period of 12 h (Ex/Em=490/520) under a xenon flashlamp. For harsh condition experiment, photostability test was done under high intensity UV lamp (Blak Ray, 100 W, 365 nm). Plates were irradiated for 10 min up to 2.5 h at 10 cm distance.

### Data availability

The data that support the findings of this study are available from the corresponding authors on request.

## Additional information

**How to cite this article**: Alamudi, S.H. *et al.* Development of background-free tame fluorescent probes for intracellular live cell imaging. *Nat. Commun.* 7:11964 doi: 10.1038/ncomms11964 (2016).

## Supplementary Material

Supplementary Information Supplementary Figures 1-26, Supplementary Tables 1-11, Supplementary Methods and Supplementary References
