# Supplementary material for: Development of background-free tame fluorescent probes for intracellular live cell imaging
Source: Nat Commun. 2016 Jun 20;7:11964. doi: 10.1038/ncomms11964 (PMC4915154; doi:10.1038/ncomms11964)
Supplement: Supplementary Information — Supplementary Figures 1-26, Supplementary Tables 1-11, Supplementary Methods and Supplementary References [file ncomms11964-s1.pdf]

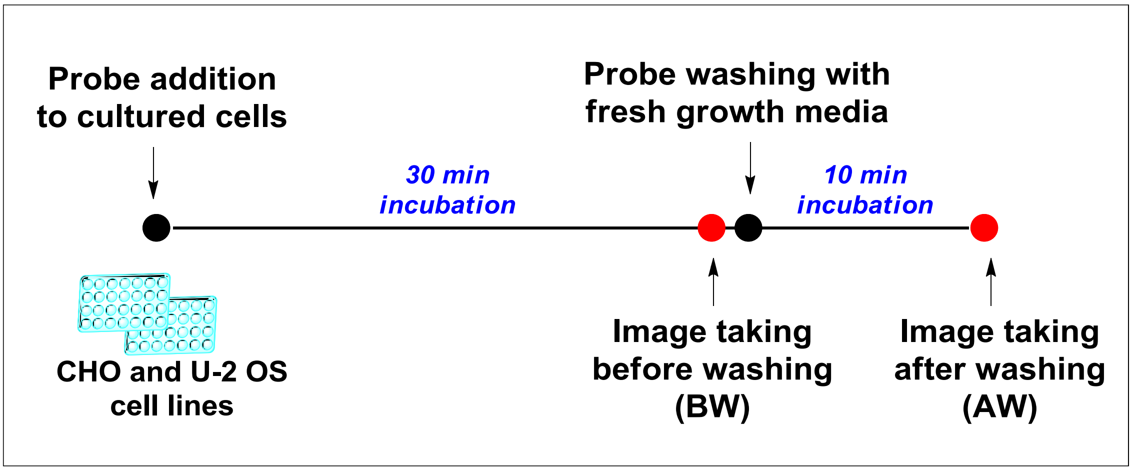

Supplementary Figure 1. Flowchart for cellular retention and influx-efflux study

200 uL fresh growth media containing probes in final concentration of 1 µM and nuclei dye Hoechst33342 was added into cultured cells. Cells were incubated for 30 minutes at 37 °C then were imaged (BW) using ImageXpress Micro™ cellular imaging system (Molecular Device). Immediately after image acquisition, the cells were washed with fresh growth media, and transferred back to a 37 °C cell incubator for further incubation. After 10 minutes, cells were again imaged (after washing image, AW). The first imaging step allowed in-flux measurement of before washing image (BW), while the second imaging step allowed out-flux measurement of after washing image (AW).

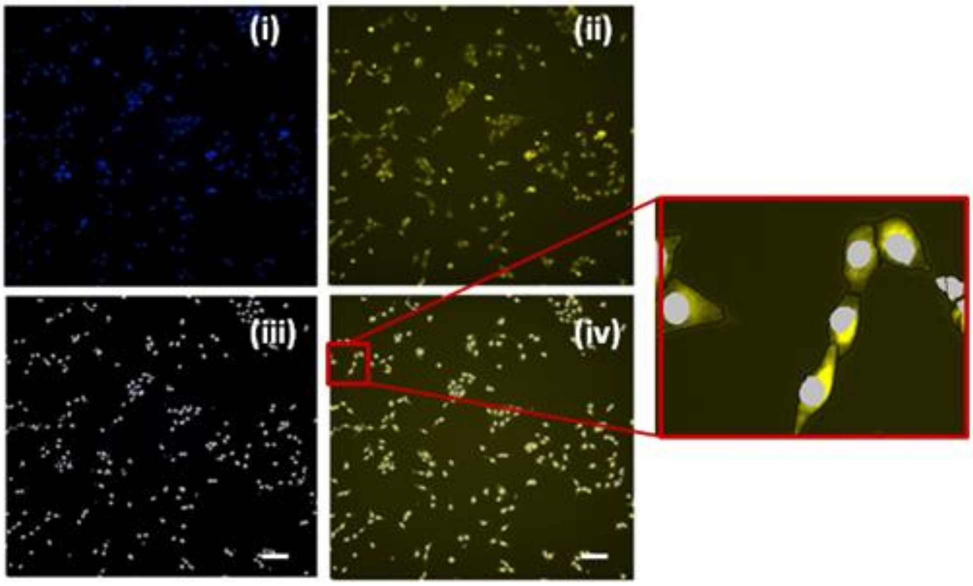

Supplementary Figure 2. Cell segmentation for quantitative image analysis.

(i) nuclear staining with Hoesch33324, (ii) probes staining throughout the cell, (iii) segmentation of nuclei from (i), (iv) segmentation of nuclei-cytoplasm from (ii) for spatial region for readout of fluorescence responce. Segmentation provides approximate cellular boundaries for cell-by-cell basis fluorescence intensity quantification. Scale bar 150 μm.

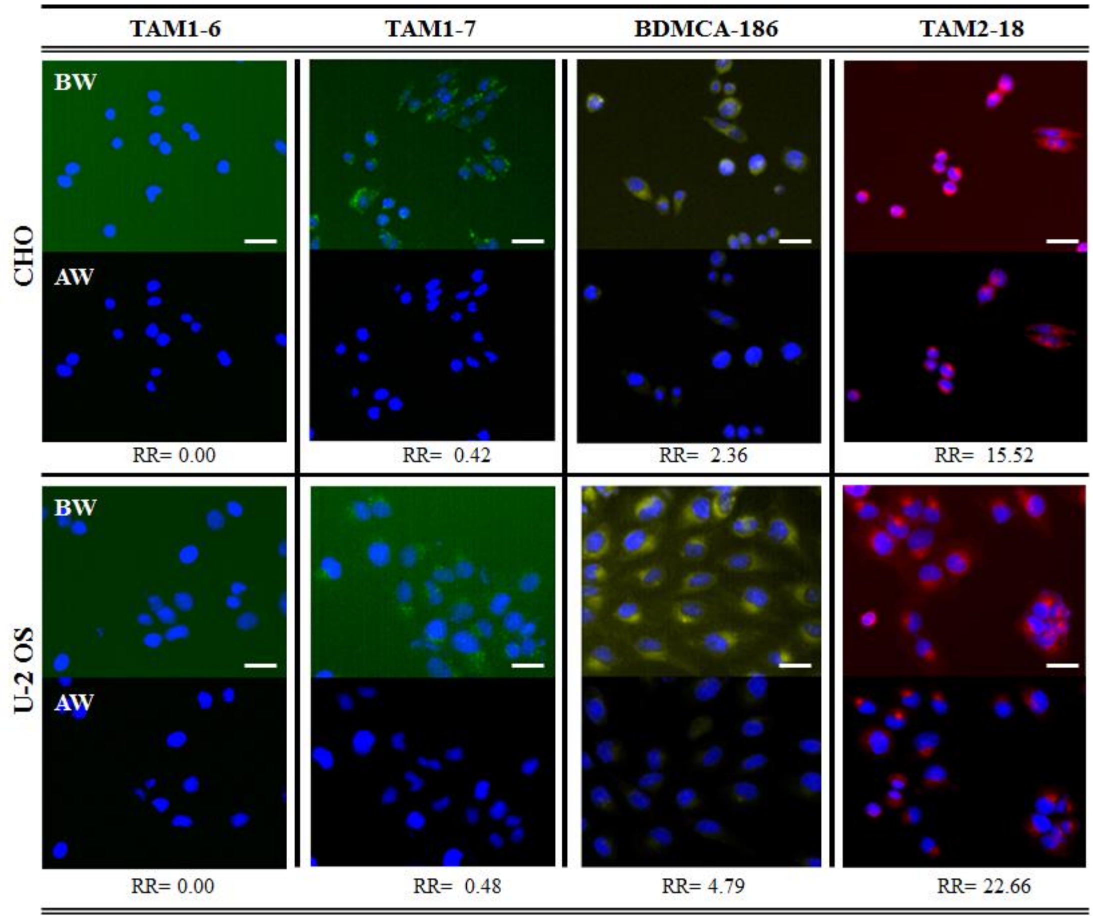
TAM1-6
TAM1-7
BDMCA-186
TAM2-18
CHO
U-2 OS
BW
AW
RR= 0.00
RR= 0.42
RR= 2.36
RR= 15.52
RR= 0.00
RR= 0.48
RR= 4.79
RR= 22.66

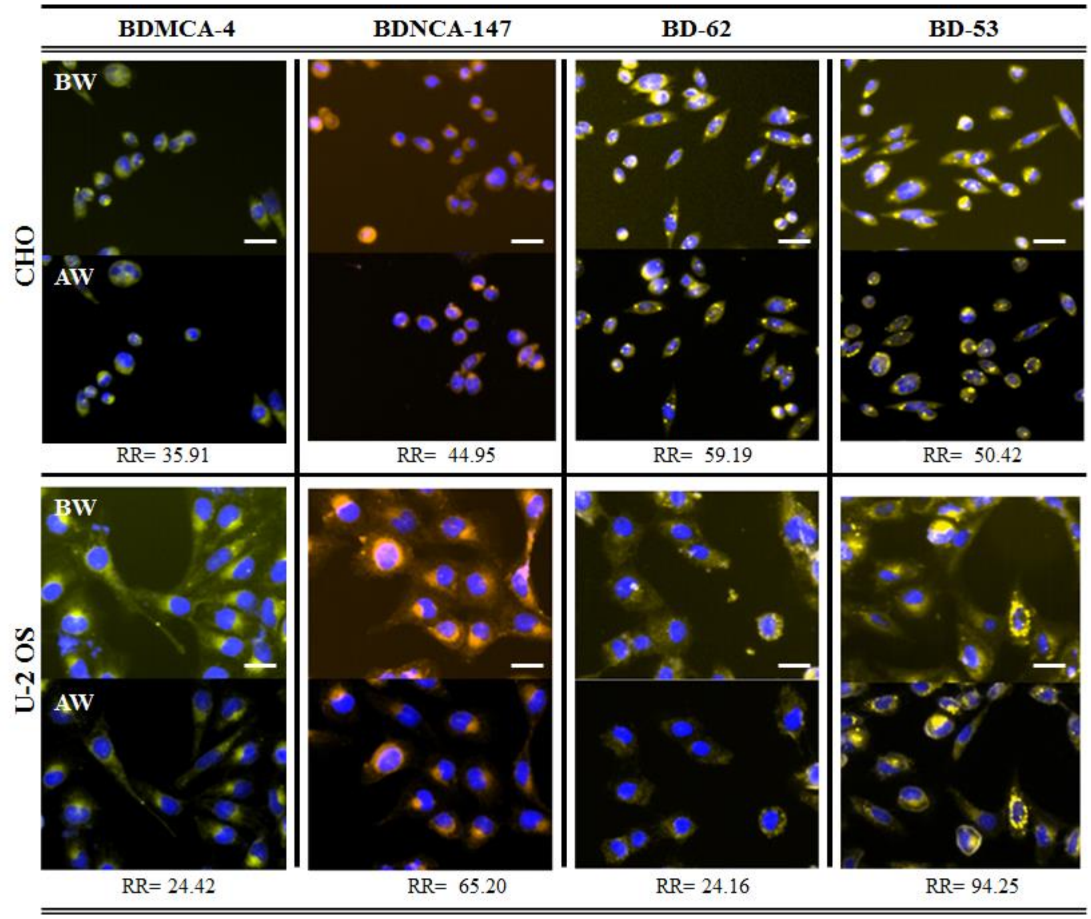
BDMCA-4
BDNCA-147
BD-62
BD-53
CHO
U-2 OS
BW
AW
RR= 35.91
RR= 44.95
RR= 59.19
RR= 50.42
RR= 24.42
RR= 65.20
RR= 24.16
RR= 94.25

Supplementary Figure 3. Representative overlayed images for different RR value.

Probes were incubated in U-2 OS and CHO cell lines at 1 uM final concentration. BW and AW indicate image before and after washing, respectively. Blue is Hoechst33324 signal and green, yellow, orange, red are probes signals according to emission wavelength of the probes. RR value is ranging from 0 to 100. Zero value indicates cell-impermeable probes, $0 < RR \geq 5$ indicates cell-permeable probess with minimal background and $RR > 5$ shows cell-permeable with high nonspecific binding probess. Scale bar, 20 μm.

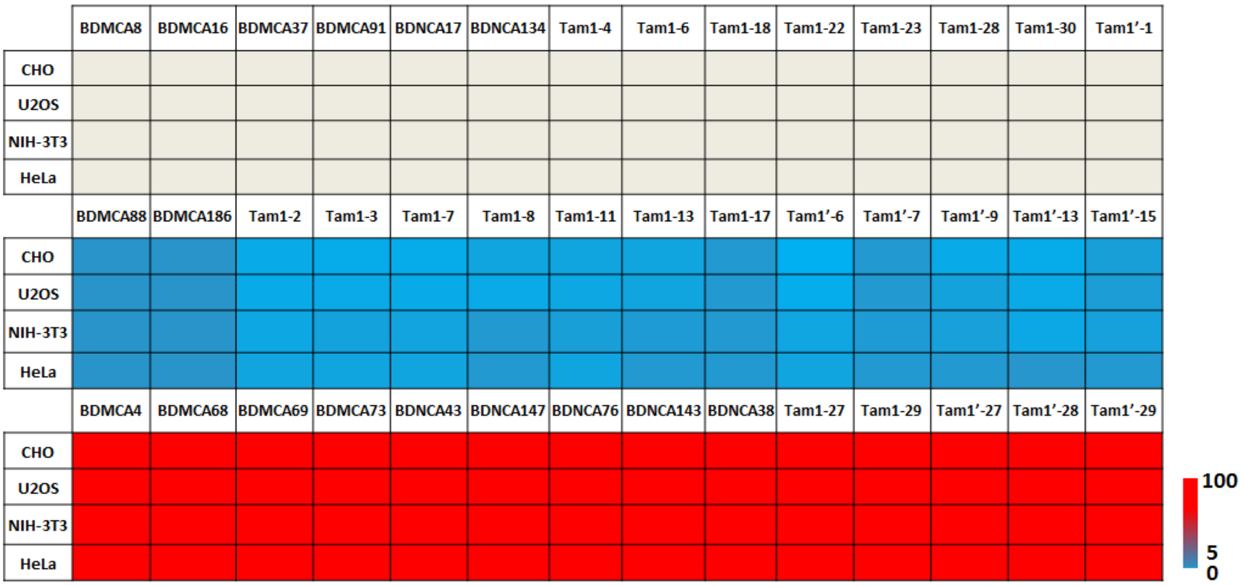

Supplementary Figure 4. Heat map of representative probes in CHO, U-2 OS, NIH-3T3 and HeLa.

Heatmap shows that probes behave similarly in the four cell lines. Grey color represents probes in N-group. The red and torquise color represents high (H-group) and low (L-group) RR value, respectively.

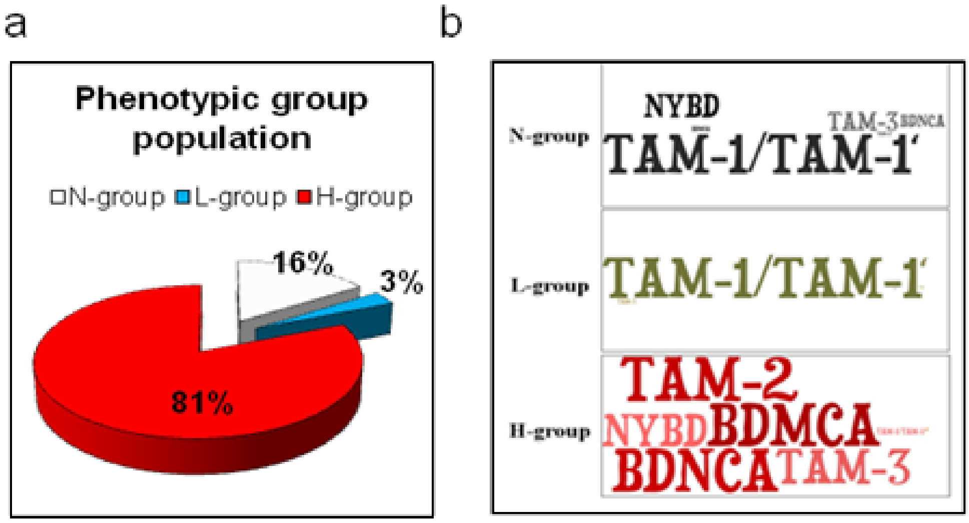

Supplementary Figure 5. Proportion of BODIPY libraries in each phenotypic group.

(a)Overall percentage of all probes in each phenotypic group. b) Word clouds diagram for distribution and portion of each library in three groups.

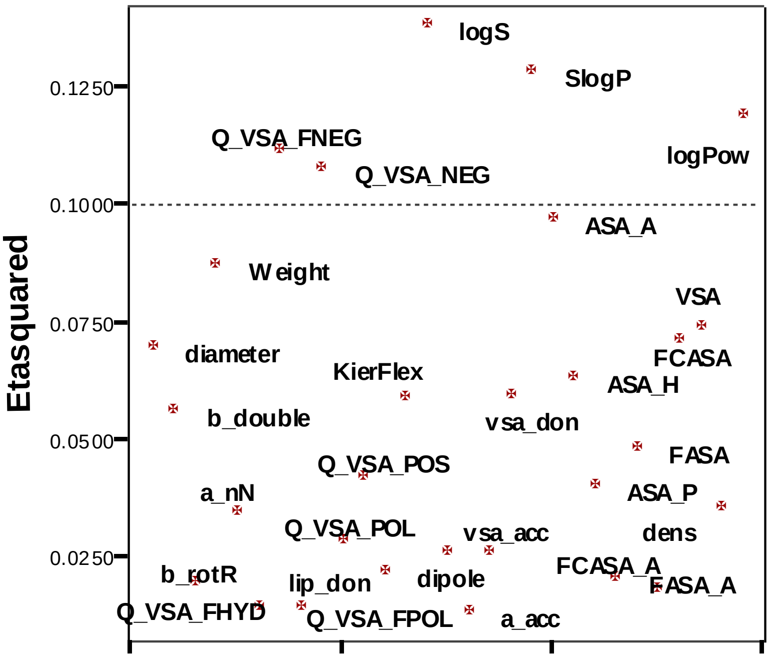

Supplementary Figure 6. Results of effect size measurement.

Descriptors with large effect (eta squared $\eta^2 \geq 0.1$) were selected.

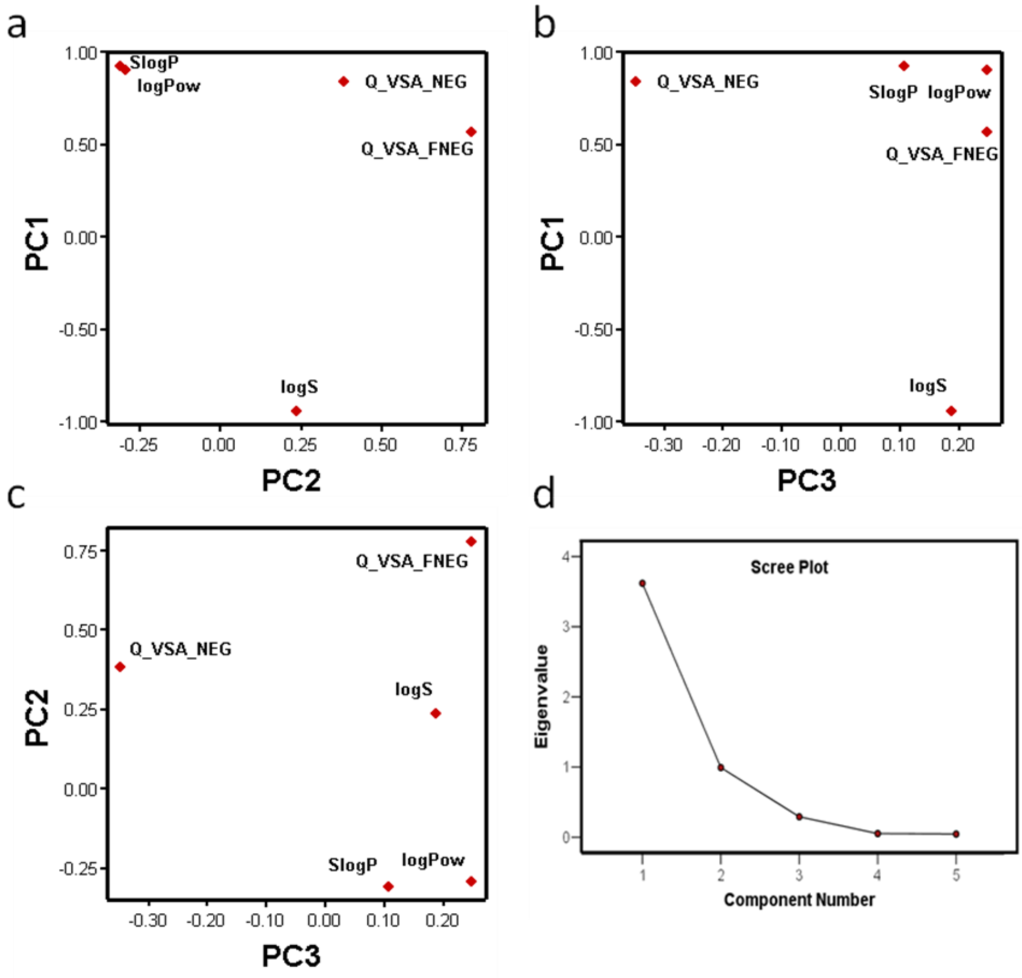

Supplementary Figure 7. PCA loading plot of five selected descriptors.

PCA plot. (a) PC1 versus PC2; (b) PC1 versus PC3, and (c) PC2 versus PC3. (d) Scree plot of the eigenvalues with respective component numbers.

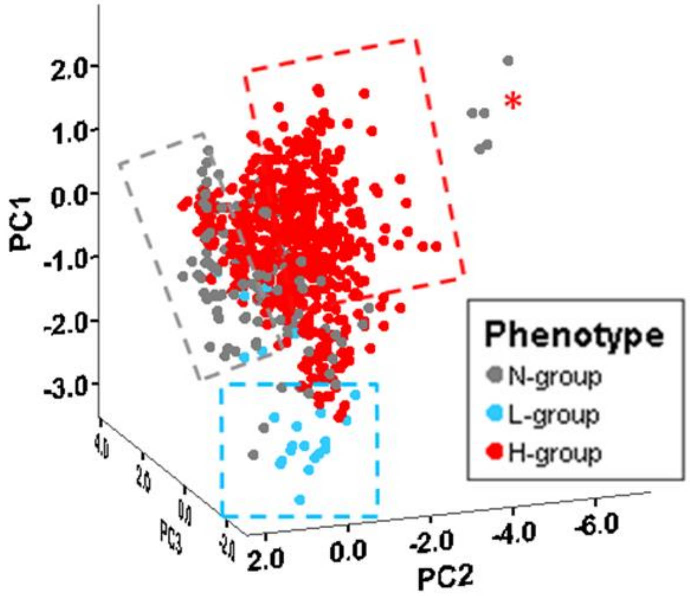

Supplementary Figure 8. PCA scores plot from probes.

Grey, turquoise and red dotted box represents area containing mostly compounds from N-group, L-group and H-group, respectively.

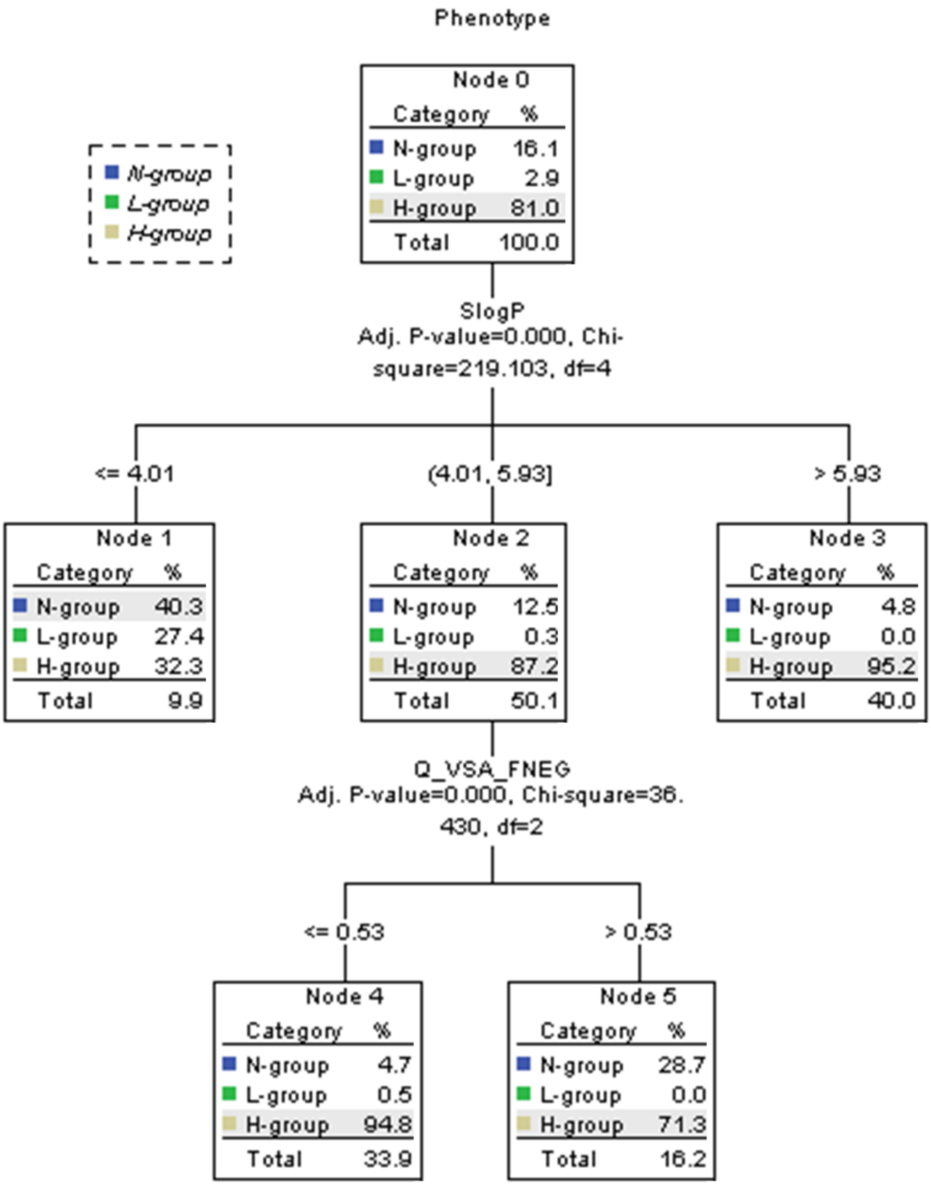

Supplementary Figure 9. The interactive decision tree diagram for phenotypic grouping of probes.

The descriptors used for the classification are SlogP, logS and Q_VSA_FNEG. Each node is idenfied with a node number.

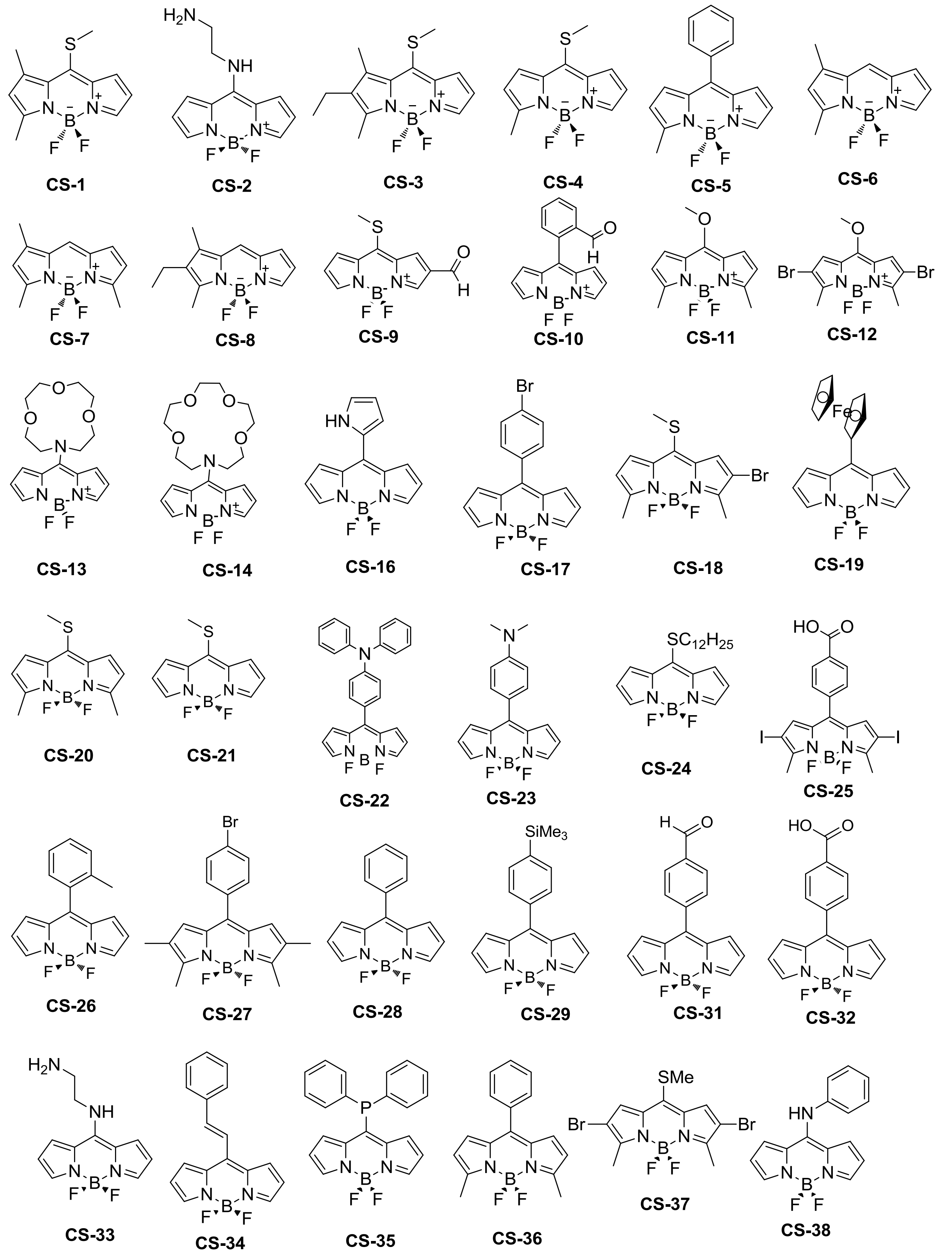

Supplementary Figure 10. Structures of EP library

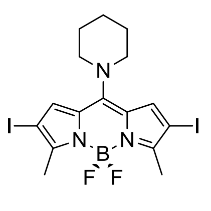
CS-39

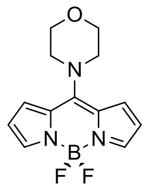
CS-40

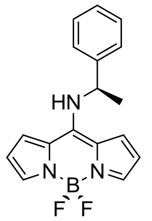
CS-41

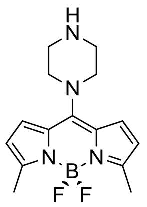
CS-42

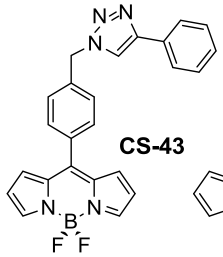
CS-43

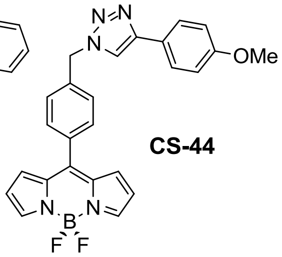
CS-44

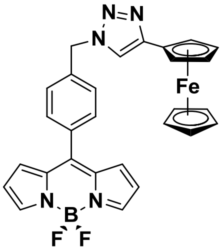
CS-45

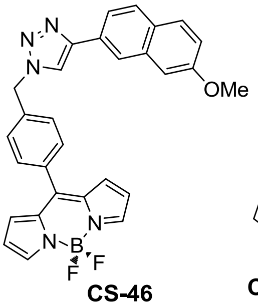
CS-46

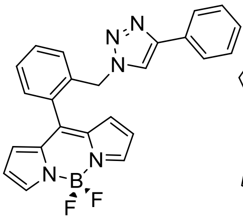
CS-47

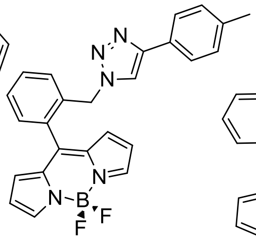
CS-48

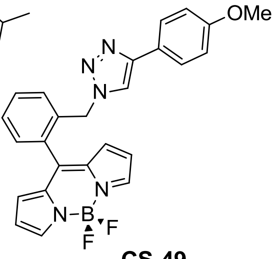
CS-49

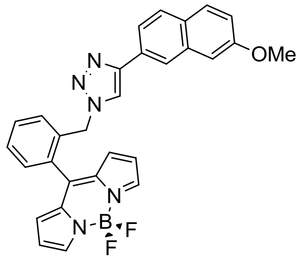
CS-50

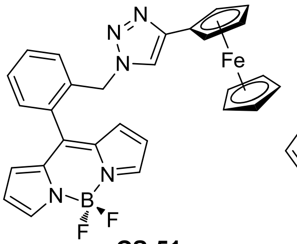
CS-51

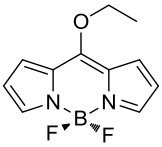
CS-52

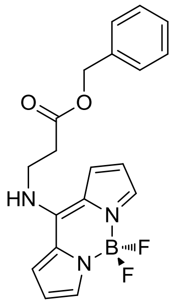
CS-53

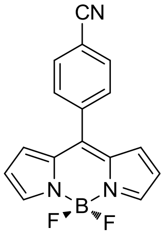
CS-54

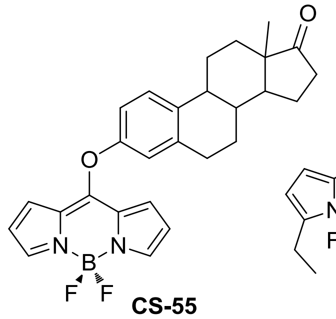
CS-55

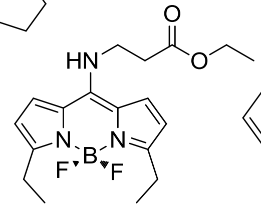
CS-56

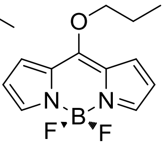
CS-57

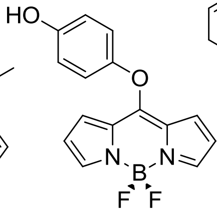
CS-58

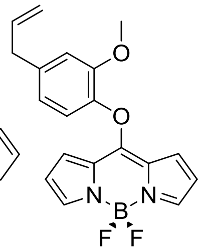
CS-59

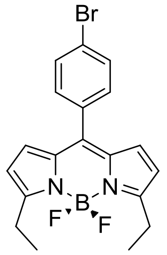
CS-60

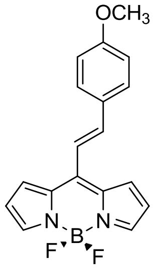
CS-61

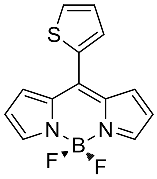
CS-62

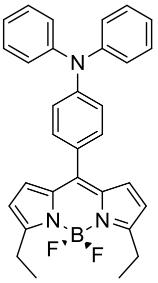
CS-63

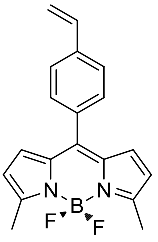
CS-64

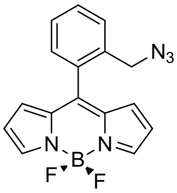
CS-65

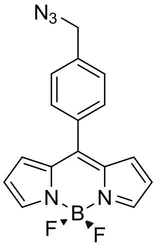
CS-66

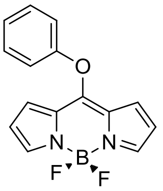
CS-67

(Cont'd) Supplementary Figure 10. Structures of EP library

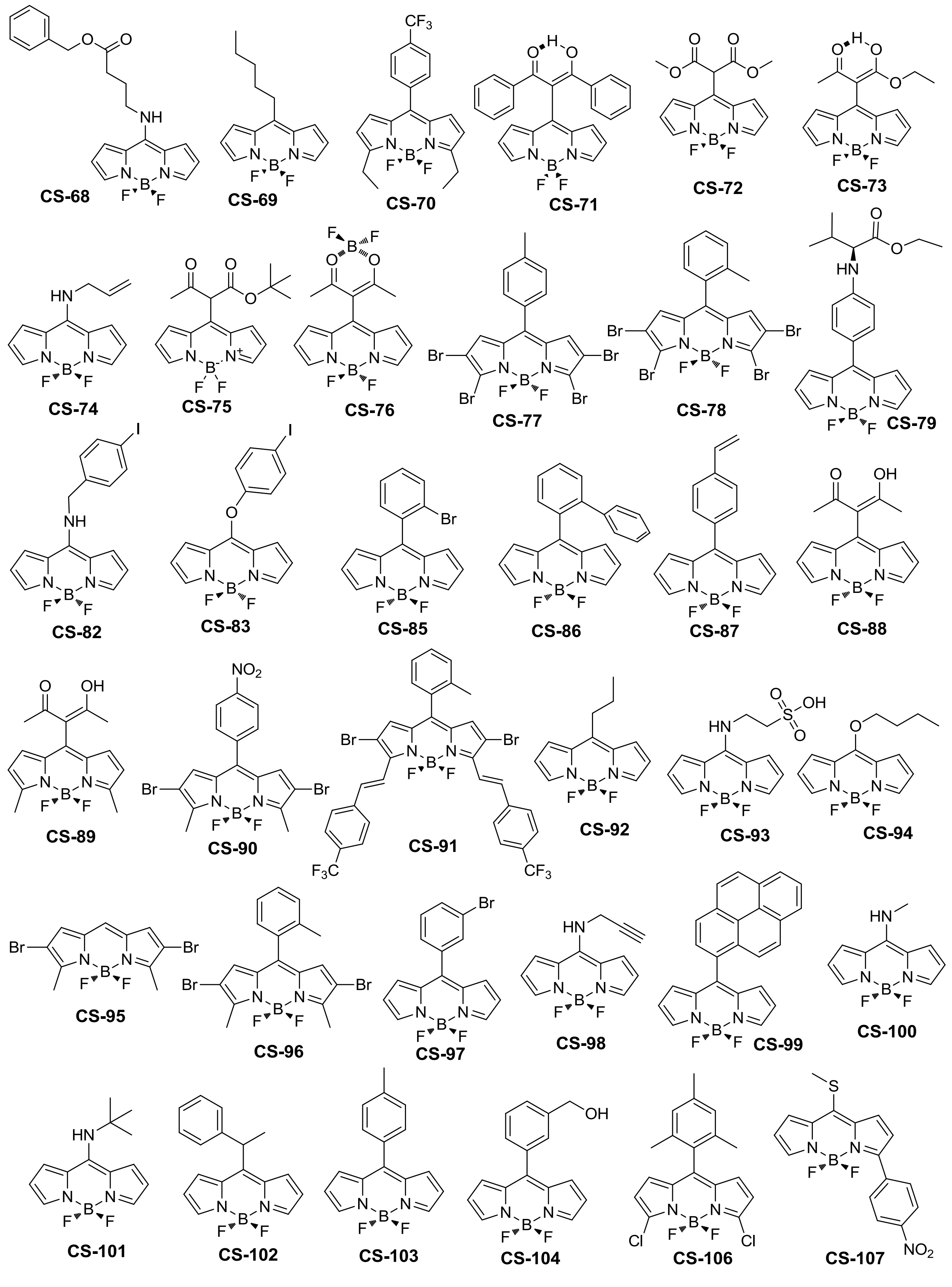

(Cont'd) Supplementary Figure 10. Structures of EP library

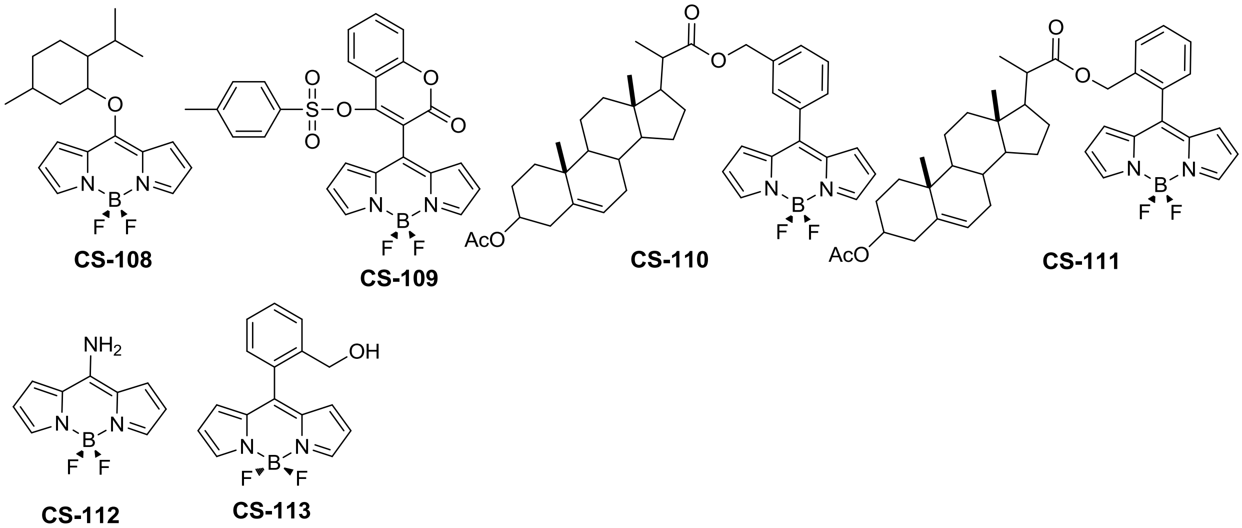

(Cont'd) Supplementary Figure 10. Structures of EP library.

The synthesis and characterization of these probes will be published elsewhere.

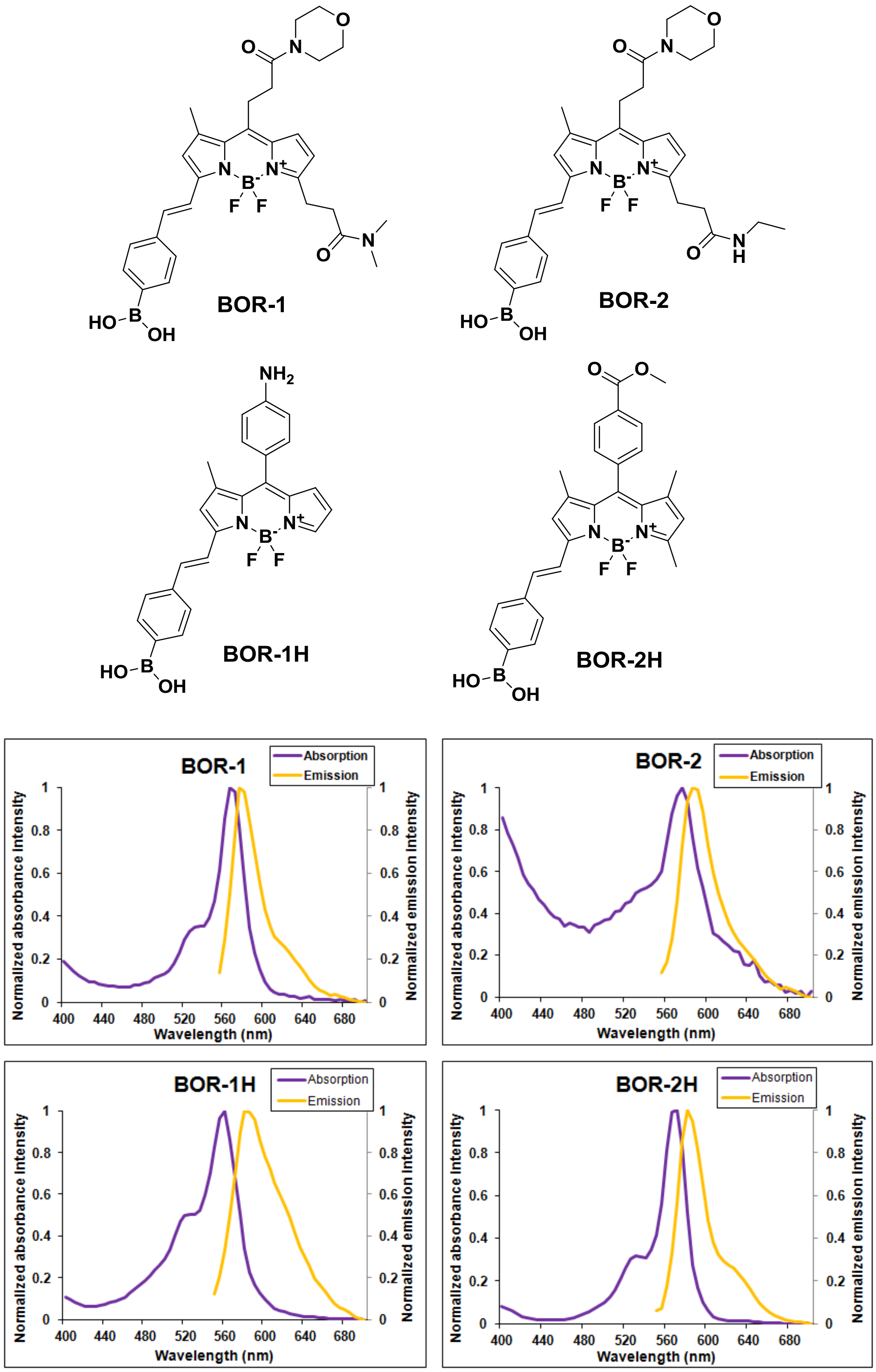

Supplementary Figure 11. Structure and absorption/emission spectra of BOR-1, BOR-2, BOR-1H and BOR-2H.

Absorbance and fluorescence emission were measured in DMSO at 10 uM of probe concentration.

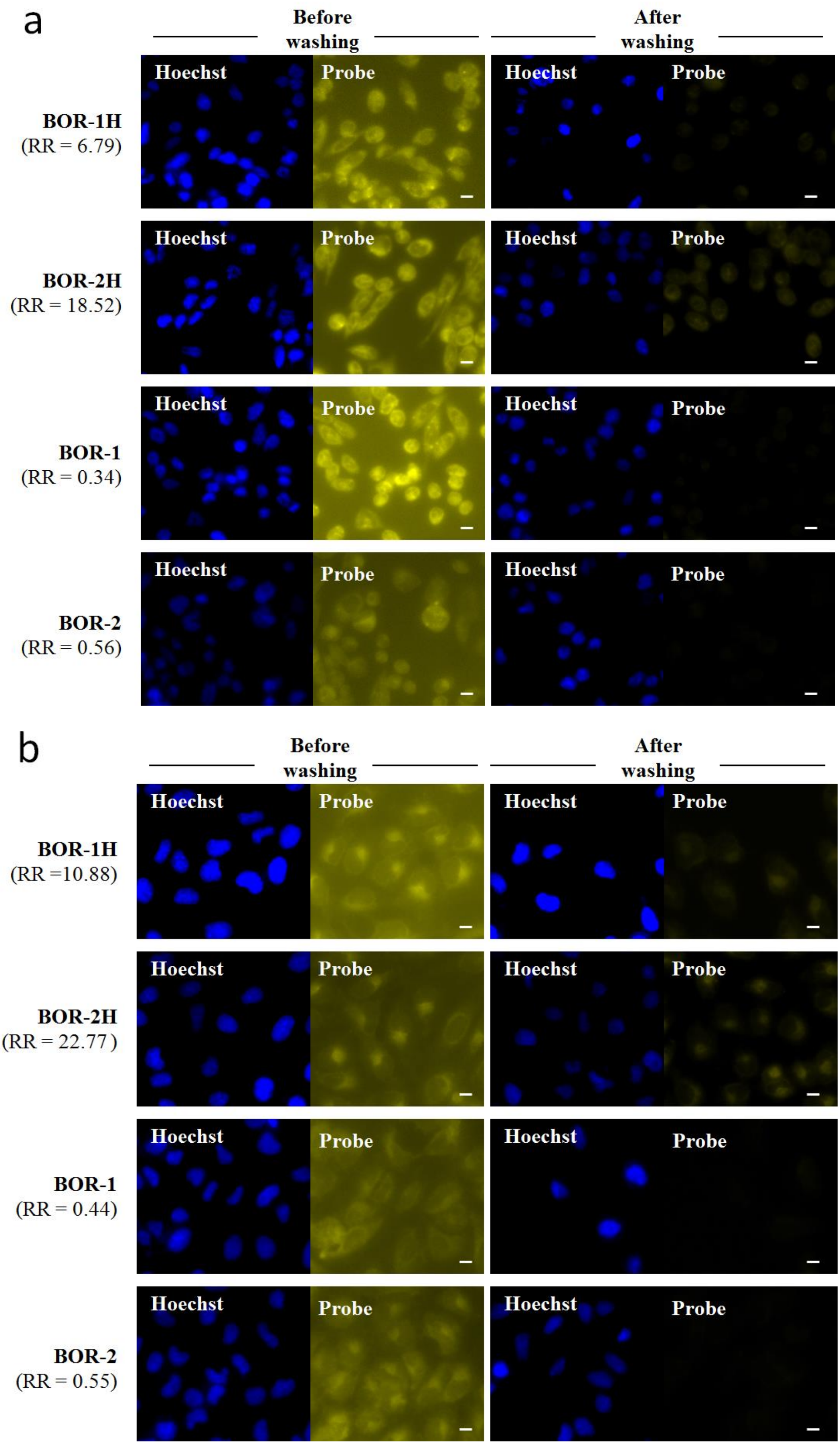
a
Before washing
After washing
Hoechst
Probe
BOR-1H
(RR = 6.79)
BOR-2H
(RR = 18.52)
BOR-1
(RR = 0.34)
BOR-2
(RR = 0.56)
b
BOR-1H
(RR =10.88)
BOR-2H
(RR = 22.77)
BOR-1
(RR = 0.44)
BOR-2
(RR = 0.55)

Supplementary Figure 12 Cellular retention BOR-1, BOR-2, BOR-1H and BOR-2H.

in CHO (a) and U-2 OS (b) cells and their RR value.

Cells were stained with probes at 1 uM final concentration for 30 min. Probe signal is significantly decreased after washing for **BOR-1** and **BOR-2.** (yellow signal from FITC channel and blue signal from DAPI channel). Scale bar, 10 μm.

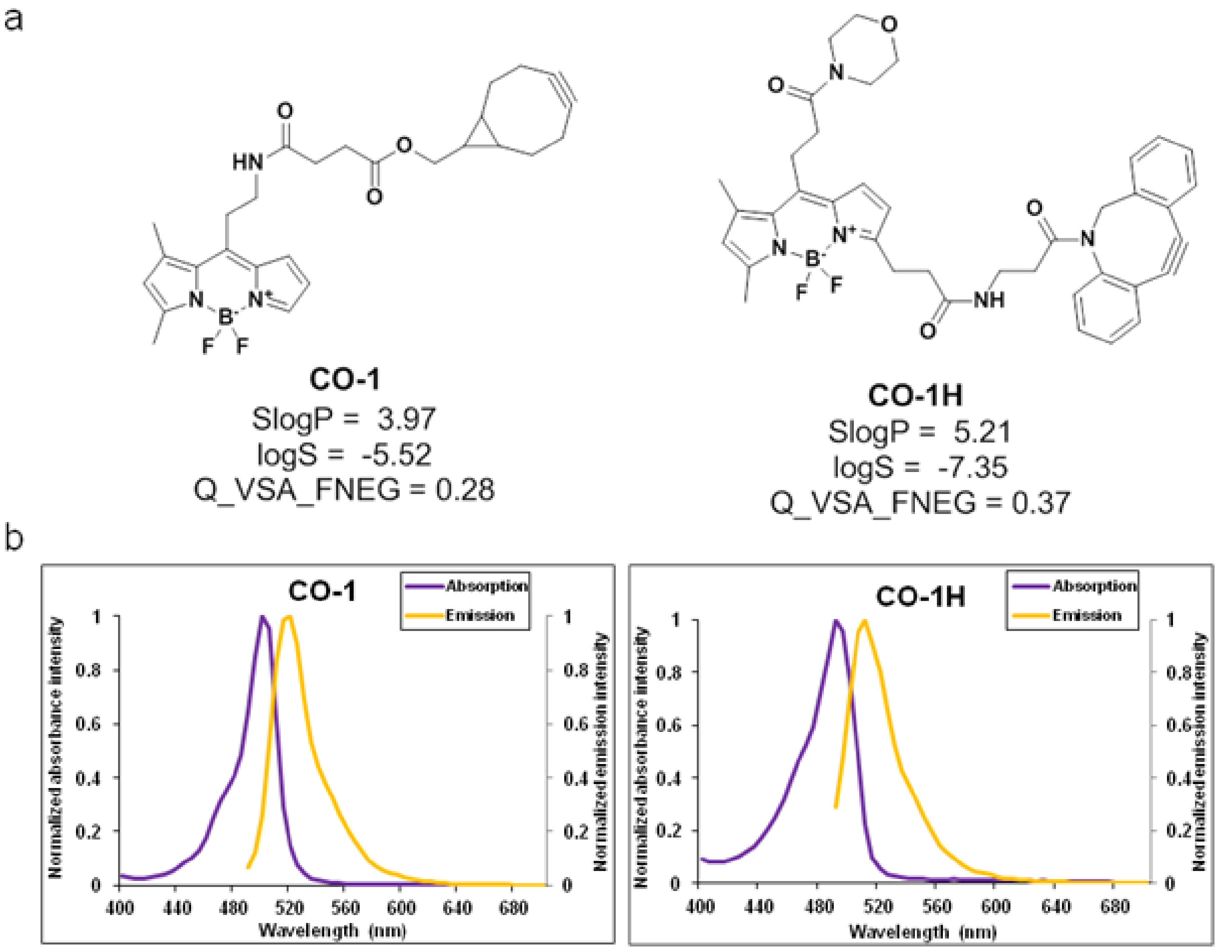

Supplementary Figure 13 Structure of CO-1 and CO-1H.

(a) Structure of **CO-1** and **CO-1H** and their descriptors values. (b) Absorption and emission spectra of **CO-1** and **CO-1H**. Absorbance and fluorescence emission were measured in DMSO at 10 uM of compound concentration.

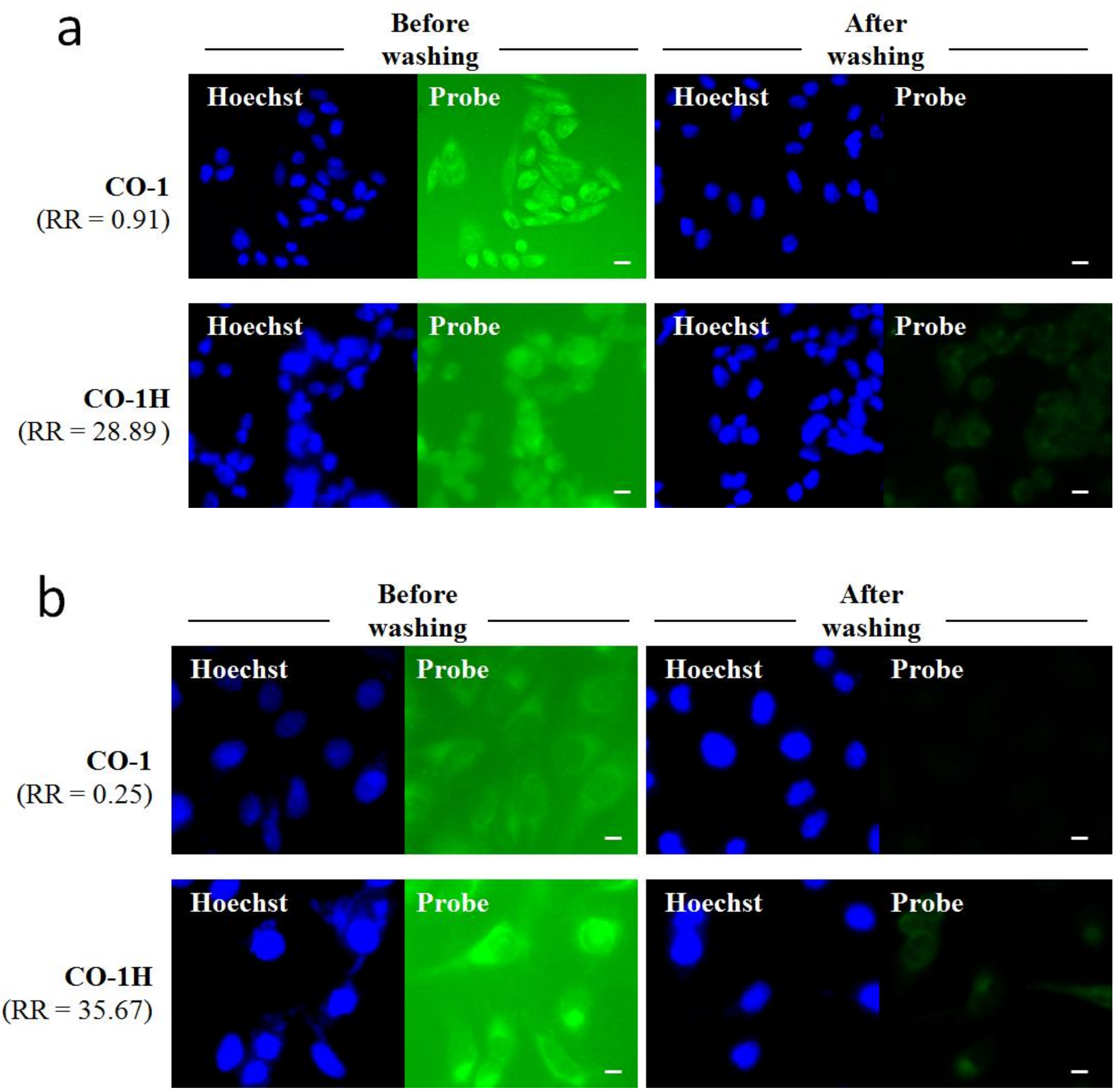

Supplementary Figure 14 Cellular retention of CO-1 and CO-1H in CHO (a) and U-2 OS (b) cells and their RR value.

Cells were stained with probes at 1 uM final concentration for 30 min. Both probes were observed to enter the cells, however, **CO-1** leave the cells after washing while **CO-1H** is more retained inside the cells (green signal from FITC channel). Scale bar, 20 µm.

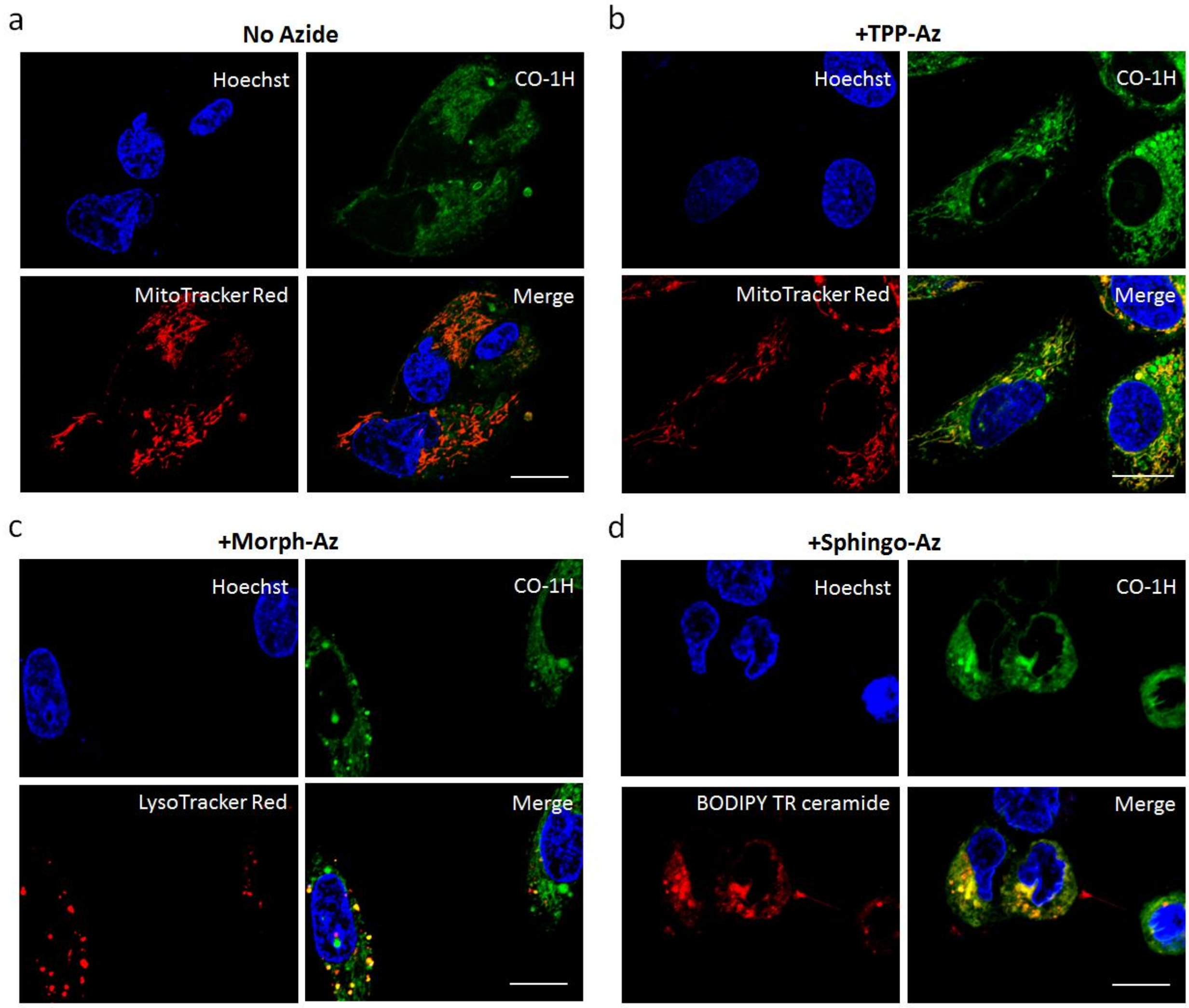

Supplementary Figure 15 Live cell imaging with CO-1H.

Fluorescence imaging of mitochondria, lysosome and golgi apparatus in U-2 OS cells labeled with **CO-1H.** Cells were incubated: (a) without azide reporter and with (b) **TPP-Az**, (c) **Morph-Az** or (d) **Sphingo-Az** in culture media at 37 °C for 1 hr with 2 μM **CO-1H** and followed by counterstaining with organelle trackers. In contrast to **CO-1**, high fluorescence background from **CO-1H** was observed. Scale bar, 15 μm.

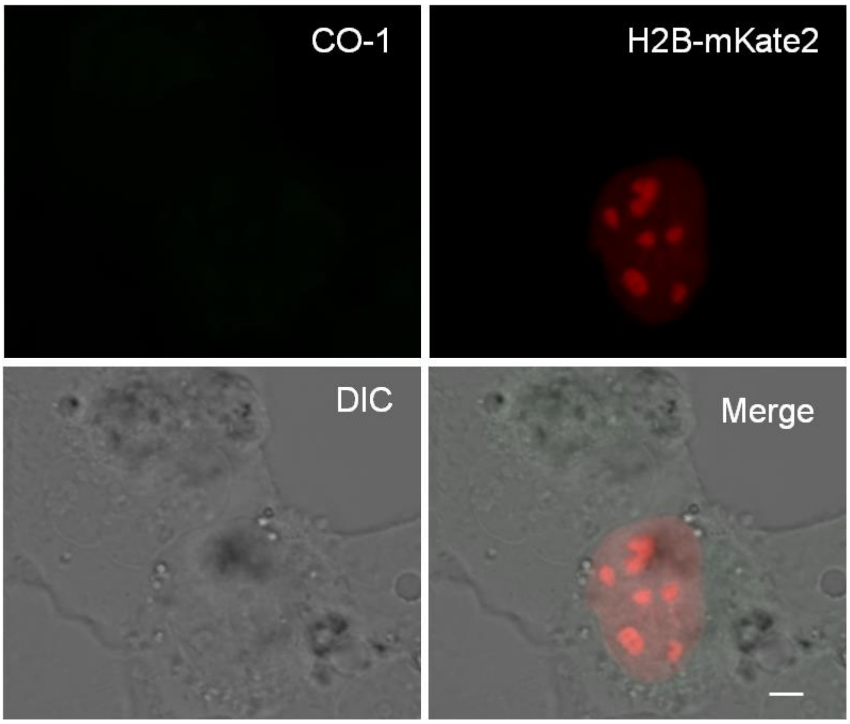

Supplementary Figure 16 CO-1 did not label wild type H2B-mKate2 in live U-2 OS cells. U-2 OS cells were transfected with plasmid pmH2B-6-mKate2. After 24 hours, cells were labeled with 10 μM **CO-1** for 90 minutes at 37 °C. Cells were washed to remove the unreacted **CO-1** and then imaged for mKate2 (red) and **CO-**1 (green) signals. Scale bar, 5 μm.

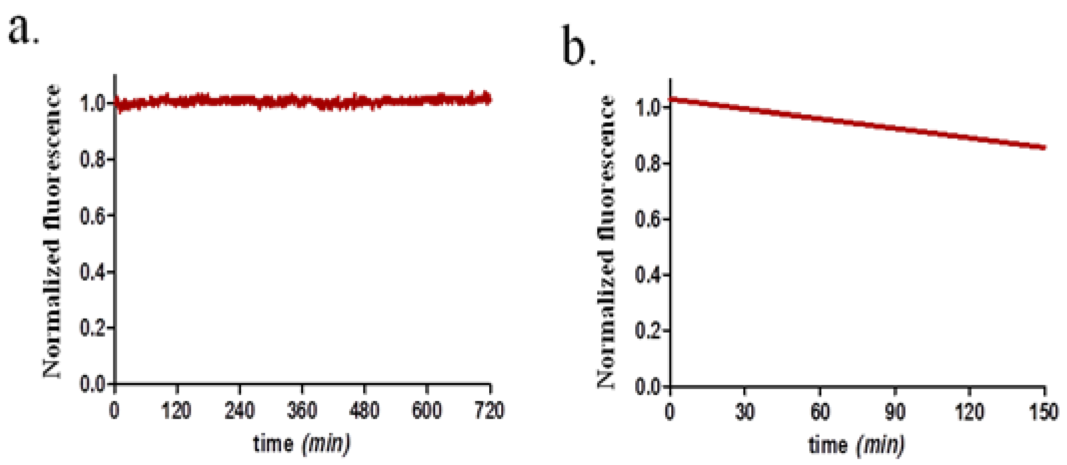

Supplementary Figure 17 Photostability analysis of CO-1.

10 µM **CO-1** solution in PBS buffer (pH 7.4) containing 1% DMSO were placed in a 96-well plates. (a) Fluorescence measurement were recorded every 30 seconds interval for a total period of 12 hours (Ex/Em = 490/520) under a xenon flashlamp. (b) Photostability test under high intensity UV lamp (Blak Ray, 100W, 365 nm). Plates were irradiated for 10 minutes up to 2.5 hours at 10 cm distance. Values are represented as means (n=3) and fitted to a non-linear regression one-phase exponential decay (GraphPad Prism 5.0)

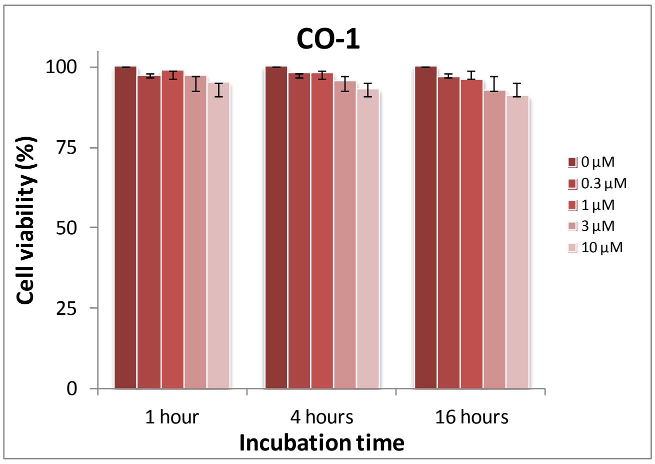

Supplementary Figure 18 Cell viability test for CO-1.

Cell viability was measured by MTS assay for concentration of probe at 0 µM, 0.3 µM, 1 µM, 3 µM and 10 µM in 1 hour, 4 hours and 16 hours incubation in U-2 OS cells. Absorbance was determined at 490 nm. Each absorbance value was subtracted with blank sample (blank sample = cells containing compound at respective concentration without MTS reagent). Cell viability was calculated by: (viable cells)% = (OD of treated sample/OD of untreated sample)×100. Data are presented as the means ± SD obtained from triplicate experiments.

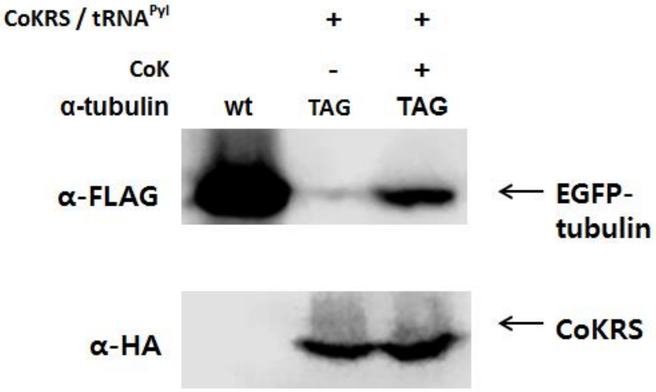

Supplementary Figure 19 Site-specific incorporation of CoK using an orthogonal tRNA^{Pyl}/CoKRS pair.

HEK293T cells were transfected with pCoKRS-tRNA (containing tRNA^{Pyl}/CoKRS pair) and pEGFP-Tub-26TAG (containing EGFP-fused α-tubulin with C-terminal FLAG tag and stop codon TAG at 26) and cultured in the presence or absence of 0.5 mM **CoK**. The cells were harvested and analyzed by Western blotting for the detection of HA-tagged CoKRS and FLAG-tagged EGFP-α-tubulin using anti-HA antibody and anti-FLAG antibody, respectively. HEK293T cells transfected with pTubwt (carrying α-tubulin wild type) was used as a control. Western blot analysis clearly indicates that EGFP-α-tubulin-26TAG can be expressed only in the presence of **CoK** and an orthogonal tRNA^{Pyl}/CoKRS pair, demonstrating that genetic incorporation of **CoK** into a specific position of α-tubulin using an orthogonal tRNA^{Pyl}/CoKRS pair.

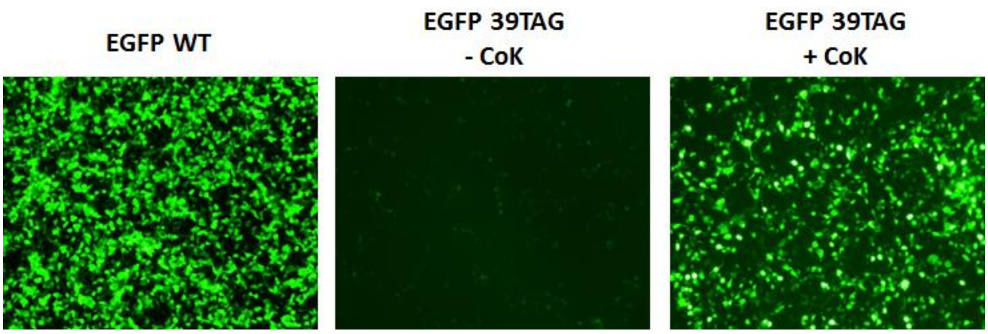

Supplementary Figure 20 Site-specific incorporation of CoK using an orthogonal tRNA^{Pyl}/CoKRS pair.

HEK293T cells were transfected with pCokRS-tRNA (containing tRNA^{Pyl}/CoKRS pair) and pEGFP-39TAG (containing EGFP with stop codon TAG at 26) and cultured in the presence or absence of 0.5 mM **CoK**. Expression of EGFP was analyzed using fluorescence microscopy. HEK293T cells transfected with pEGFP (carrying EGFP wild type) was used as a control. Fluorescence images clearly show that EGFP-39TAG can be expressed only in the presence of **CoK**, demonstrating genetic incorporation of **CoK** into a specific position of EGFP using an orthogonal tRNA^{Pyl}/CoKRS pair.

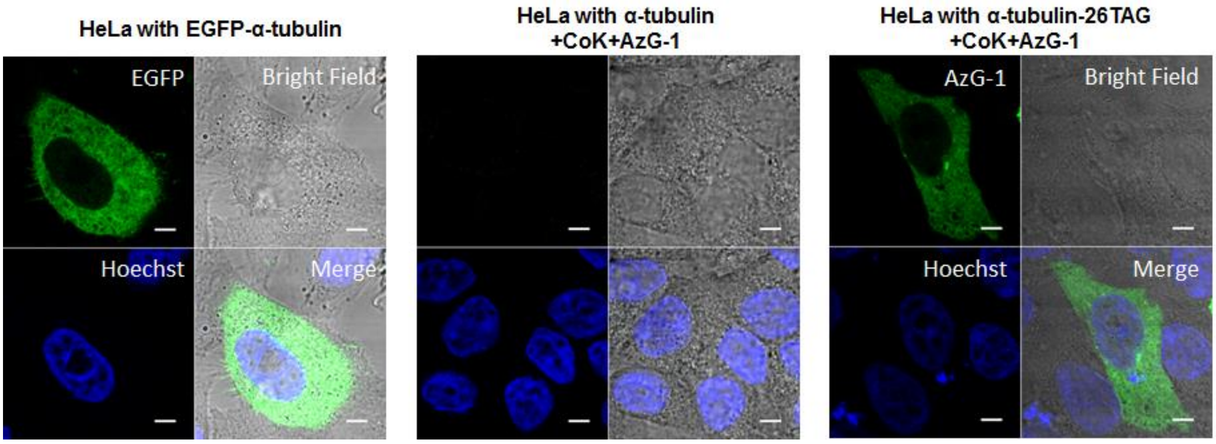

Supplementary Figure 21 Fluorescence imaging of α-tubulin in live HeLa.

HeLa cells were co-transfected with plasmids pCoKRS-tRNA and pTub-26TAG or plasmid pTubwt followed by sequential treatment with **CoK** and **AzG-1** as described above. Live cell images clearly show that the dye **AzG-1** is conjugated specifically with **CoK**-bearing α-tubulin in HeLa cells. Scale bar, 10 μm.

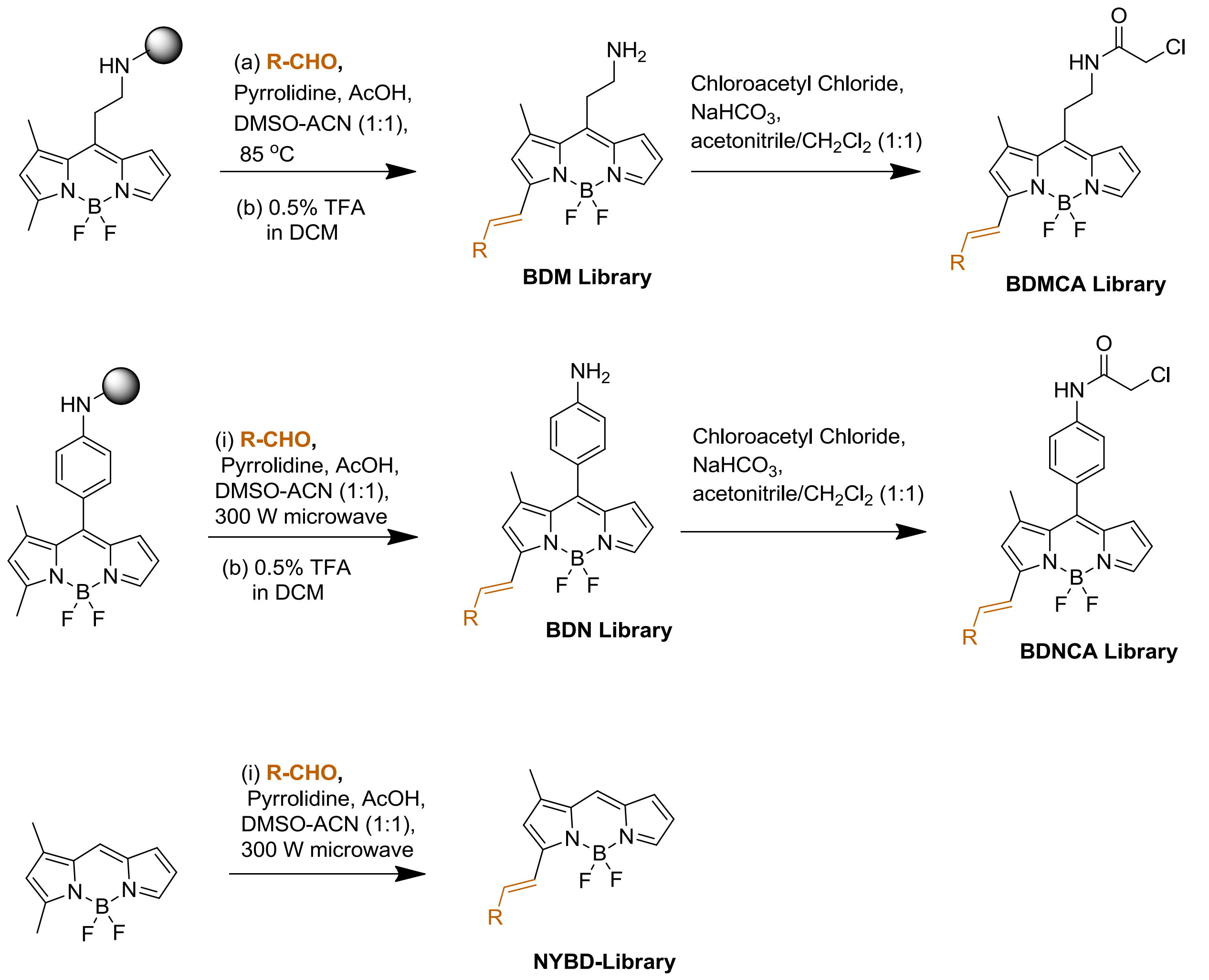

R-CHO = Aldehyde building blocks

(Reported detailed synthetic scheme: BDM[6], BDN[8] and NYBD[9,10])

Supplementary Figure 22 General synthetic scheme of the BDMCA, BDNCA and NYBD libraries.

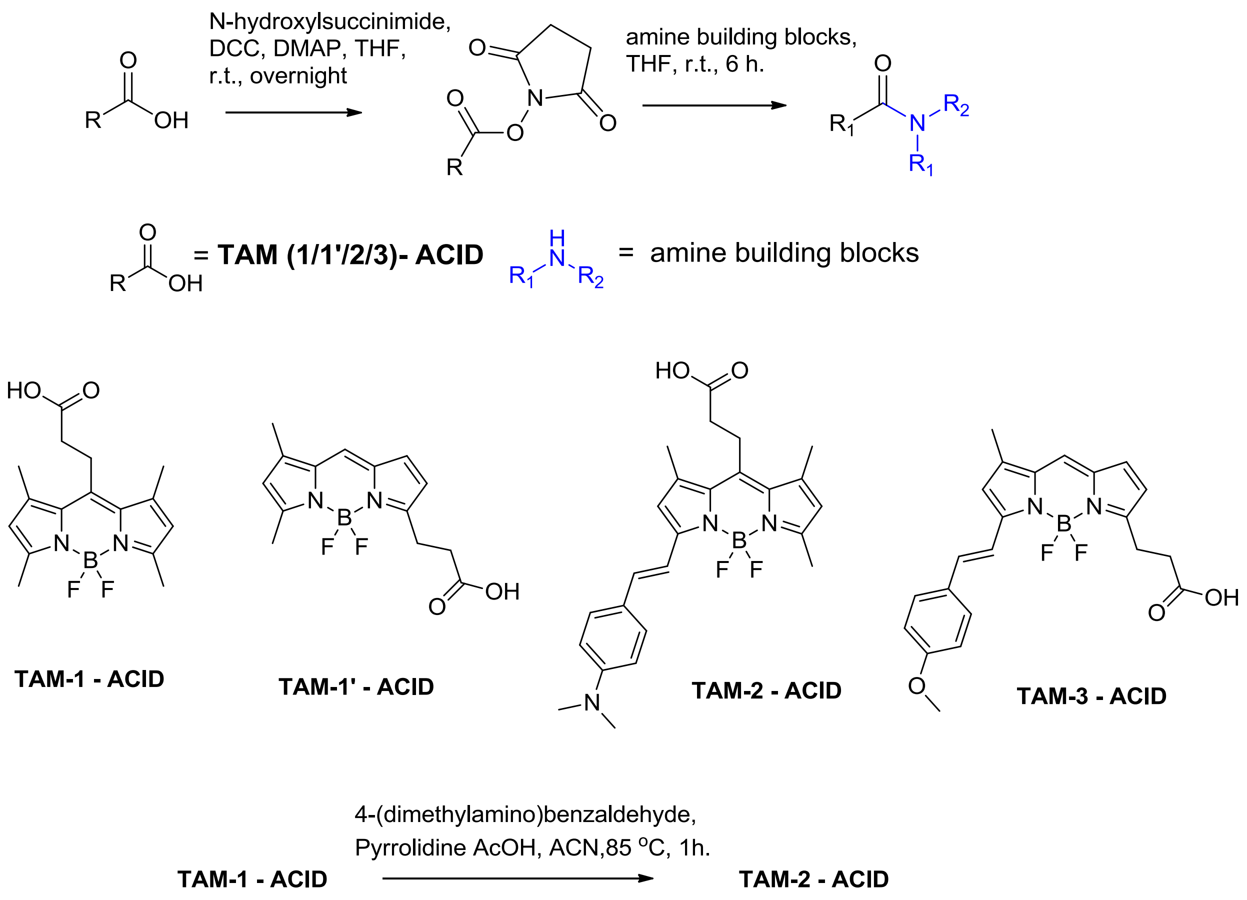

Supplementary Figure 23 General synthetic scheme of the TAM-1, TAM-1', TAM-2 and TAM-3 libraries.

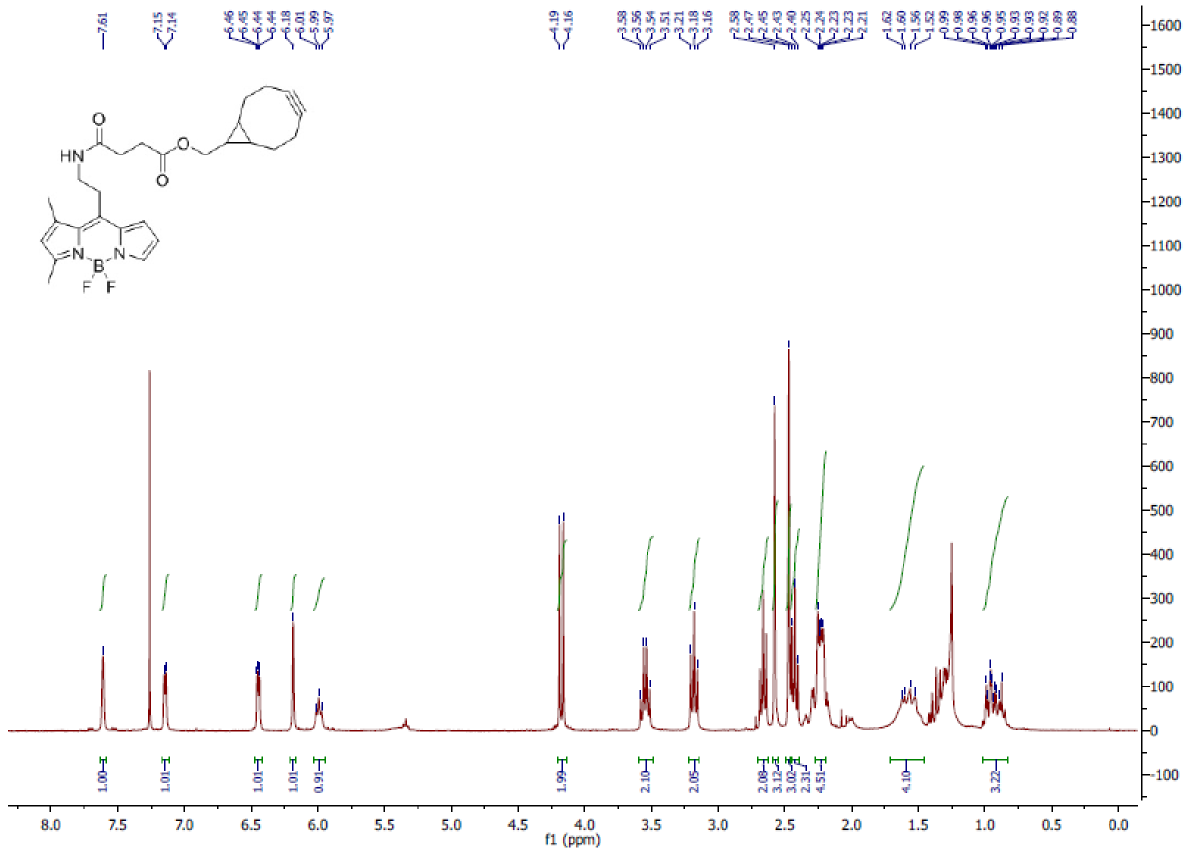

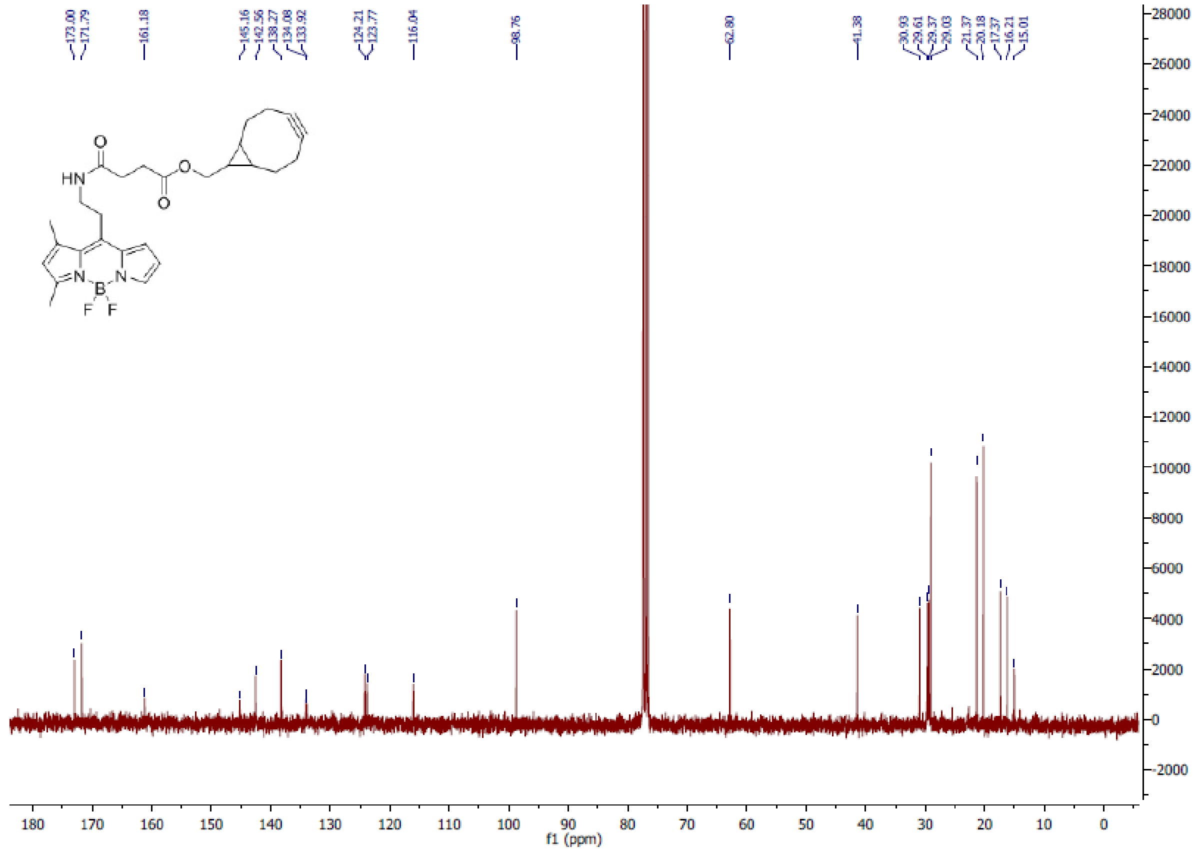

Supplementary Figure 24 ^{1}H (top) and ^{13}C (bottom) NMR spectra for CO-1.

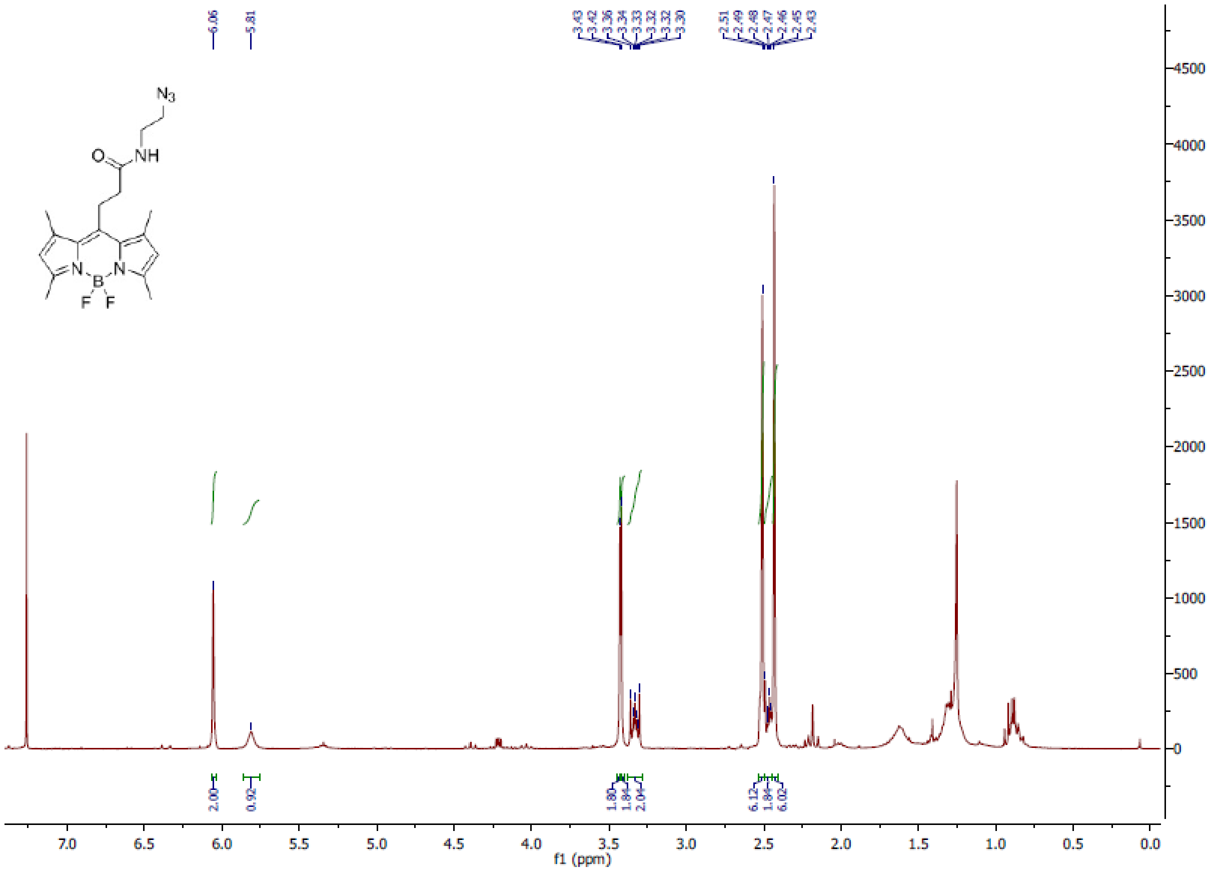

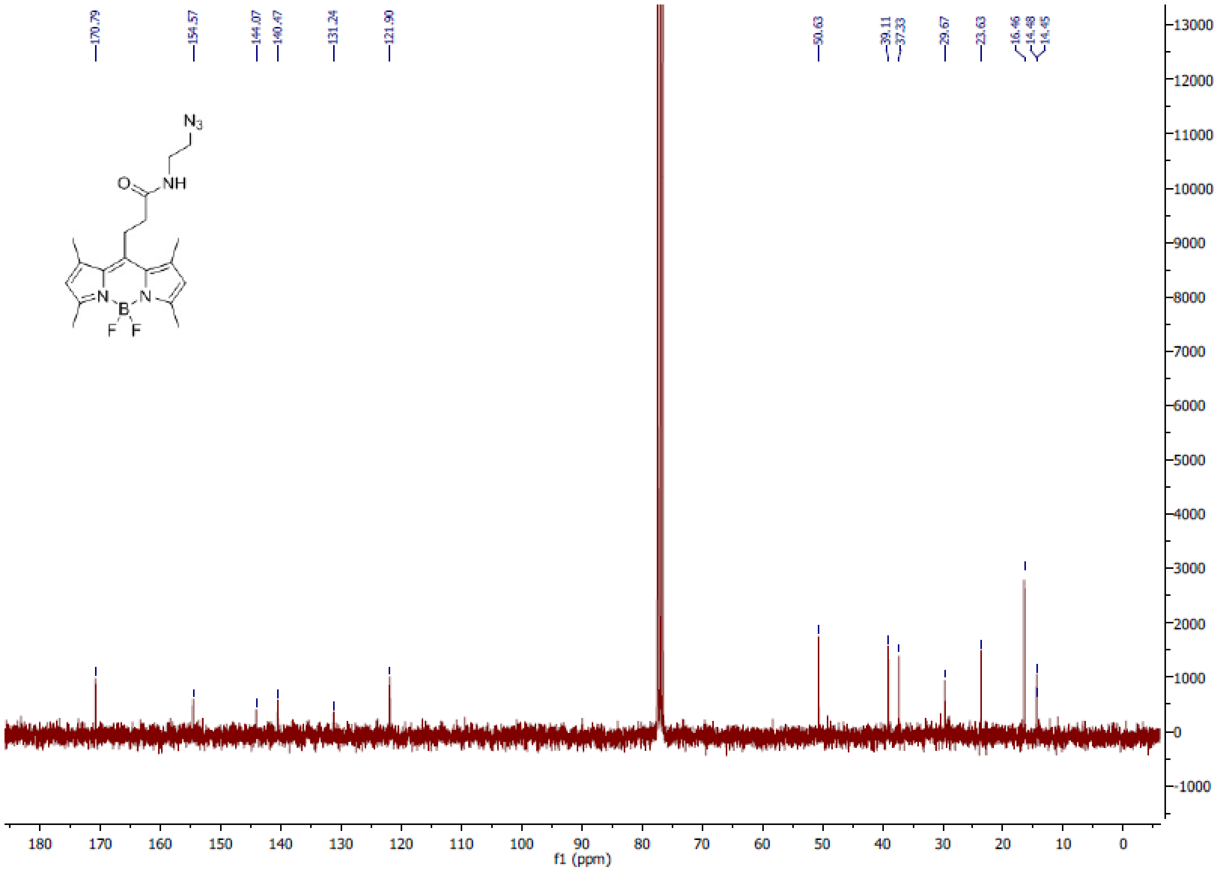

Supplementary Figure 25 ^{1}H (top) and ^{13}C (bottom) NMR spectra for AzG-1.

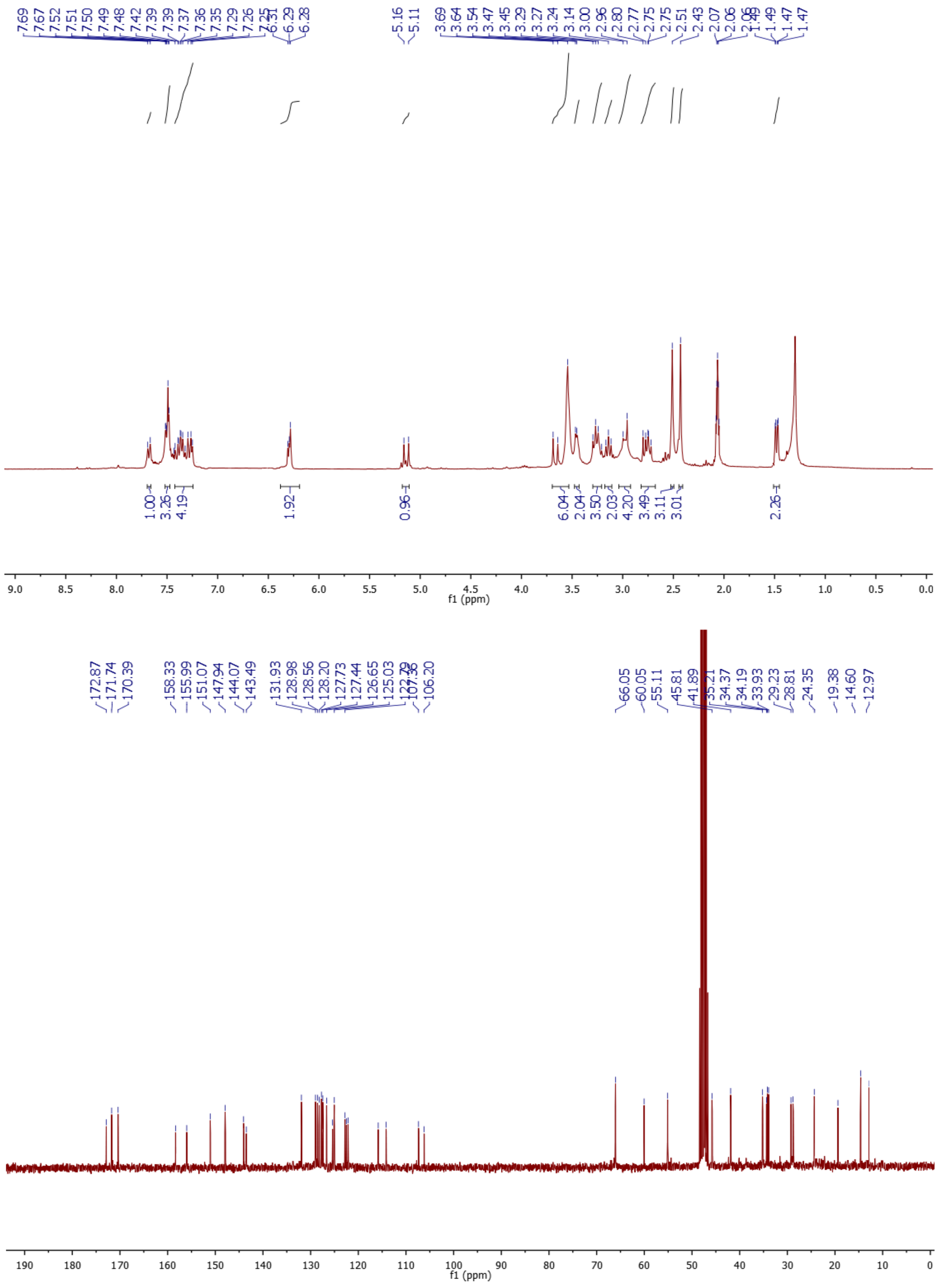

Supplementary Figure 26 ^{1}H (top) and ^{13}C (bottom) NMR spectra for CO-1H.

Supplementary Table 1 **List of representative molecular descriptors generated by MOE software**

Type	Descriptors
Atom counts and bond counts	a_aro, a_count, a_heavy, a_IC, a_ICM, a_nB, a_nBr, a_nC,a_nCl, a_nF, a_nH, a_nI, a_nN, a_nO, a_nP, a_nS, b_1rotN,b_1rotR, b_ar, b_count, b_double, b_heavy, b_rotN, b_rotR,b_single, b_triple, chiral, chiral_u, lip_acc, lip_don, rings,VAdjEq, VAdjMa
Physical properties	apol, bpol, density, FCharge, logP(o/w), logS, mr, SlogP, SMR, TPSA, vdw_area, vdw_vol, Weight
Subdivided surface areas	SlogP_VSA0, SlogP_VSA1, SlogP_VSA2, SlogP_VSA3,SlogP_VSA4, SlogP_VSA5, SlogP_VSA6, SlogP_VSA7,SlogP_VSA8, SlogP_VSA9, SMR_VSA0, SMR_VSA1,SMR_VSA2, SMR_VSA3, SMR_VSA4, SMR_VSA5,SMR_VSA6, SMR_VSA7
Pharmacophore feature descriptors	a_acc, a_acid, a_base, a_don, a_hyd, vsa_acc, vsa_acid,vsa_base, vsa_don, vsa_hyd, vsa_other, vsa_pol
Kier&Hall connectivity and kappa shape indices	chi0, chi0_C, chi0v, chi0v_C, chi1, chi1_C, chi1v, chi1v_C, Kier1,Kier2, Kier3, KierA1, KierA2, KierA3, KierFlex, zagreb
Partial charge descriptors	PEOE_PC+, PEOE_PC-, PEOE_RPC+, PEOE_RPC-,PEOE_VSA+0, PEOE_VSA+1, PEOE_VSA+2,PEOE_VSA+3, PEOE_VSA+4, PEOE_VSA+5,PEOE_VSA+6, PEOE_VSA-0, PEOE_VSA-1, PEOE_VSA-2,PEOE_VSA-3, PEOE_VSA-4, PEOE_VSA-5, PEOE_VSA-6,PEOE_VSA_FHYD, PEOE_VSA_FNEG,PEOE_VSA_FPNEG, PEOE_VSA_FPOL, PEOE_VSA_FPOS,PEOE_VSA_FPPOS, PEOE_VSA_HYD, PEOE_VSA_NEG,PEOE_VSA_PNEG, PEOE_VSA_POL, PEOE_VSA_POS,PEOE_VSA_PPOS, Q_PC+, Q_PC-, Q_RPC+, Q_RPC-,Q_VSA_FHYD, Q_VSA_FNEG, Q_VSA_FPNEG,Q_VSA_FPOL, Q_VSA_FPOS, Q_VSA_FPPOS,Q_VSA_HYD, Q_VSA_NEG, Q_VSA_PNEG, Q_VSA_POL,Q_VSA_POS, Q_VSA_PPOS

Supplementary Table 2 Thirty-nine molecular descriptors selected for feature selection

No	Descriptor	Description	Class*
1	diameter	Largest value in the distance matrix	2D[1]
2	b_double	Number of double bonds. Aromatic bonds are not considered to be double bonds	2D
3	b_rotR	Fraction of rotatable bonds	2D
4	Weight	Molecular weight	2D
5	a_nN	Number of nitrogen atoms	2D
6	a_nO	Number of oxygen atoms	2D
7	Q_VSA_FHYD	Fractional hydrophobic van der Waals surface area	2D
8	Q_VSA_FNEG	Fractional negative van der Waals surface area	2D
9	Q_VSA_FPOL	Fractional polar van der Waals surface area	2D
10	Q_VSA_NEG	Total negative van der Waals surface area	2D
11	Q_VSA_POL	Total polar van der Waals surface area	2D
12	Q_VSA_POS	Total positive van der Waals surface area	2D
13	lip_acc	The number of hydrogen bond acceptor atoms (O and N atoms)	2D
14	lip_don	The number of hydrogen dond donor (OH and NH atoms)	2D
15	KierFlex	Kier molecular flexibility index	2D[2]
16	logS	Log of the solubility in water	2D[3]
17	dipole	Dipole moment calculated from the partial charges of the molecule	3D
18	a_acc	Number of hydrogen bond acceptor atoms (not counting acidic atoms but counting atoms that are both hydrogen bond donors and acceptors such as -OH)	2D
19	a_don	Number of hydrogen bond donor atoms (not counting basic atoms but counting atoms that are both hydrogen bond donors and acceptors such as -OH)	2D
20	vsa_acc	Approximation to the sum of VDW surface areas of pure hydrogen bond acceptors (not counting acidic atoms and atoms that are both hydrogen bond donors and acceptors such as -OH)	2D
21	vsa_don	Approximation to the sum of VDW surface areas of pure hydrogen bond donors (not counting basic atoms and atoms that are both hydrogen bond donors and acceptors such as -OH)	2D
22	vsa_pol	Approximation to the sum of VDW surface areas of polar (both hydrogen bond donors and acceptors) atoms (such as -OH)	2D
23	SlogP	Log of the octanol/water partition coefficient (including implicit hydrogens). This property is an atomic contribution model that calculates logP from the given structure; i.e., the correct protonation state (washed structures)	2D[4]
24	ASA_A	Water accessible surface area of all atoms with negative partial charge	3D
25	ASA_H	Water accessible surface area of all hydrophobic atoms	3D
26	ASA_P	Water accessible surface area of all polar atoms	3D
27	DASA	Absolute value of the difference between water accessible surface area of all atoms with negative and positive partial charge	3D

28	DCASA	Absolute value of the difference between negative and positive charge weighted surface area	3D
29	FASA_A	Fractional of water accessible surface area of all atoms with negative partial charge	3D
30	FASA	Fractional of water accessible surface area of all atoms with positive partial charge	3D
31	FASA_H	Fractional of water accessible surface area of all hydrophobic atoms	3D
32	FASA_P	Fractional of water accessible surface area of all polar atoms	3D
33	FCASA_A	Fractional of negative charge weighted surface area	3D
34	FCASA	Fractional of positive charge weighted surface area	3D
35	VSA	van der Waals surface area. A polyhedral representation is used for each atom in calculating the surface area	3D
36	TPSA	Topological polar surface area	3D[5]
37	dens	Molecular mass density	3D
38	glob	Globularity. A value of 1 indicates a perfect sphere while a value of 0 indicates a two- or one-dimensional object	3D
39	logPow	Log of the octanol/water partition coefficient	2D

*2D: two-dimensional molecular descriptors, 3D: three-dimensional molecular descriptors

Supplementary Table 3 Results of Shapiro-Wilk normality test

Descriptor	Phenotype	Shapiro-Wilk			Descriptor	Phenotype	Shapiro-Wilk		
		Statistic	df	Sig.			Statistic	df	Sig.
diameter	N-group	0.864	127.000	0.000	vsa_acc	N-group	0.765	127.000	0.000
	L-group	0.951	24.000	0.289		L-group	0.624	24.000	0.000
	H-group	0.967	654.000	0.000		H-group	0.863	654.000	0.000
b_double	N-group	0.702	127.000	0.000	vsa_don	N-group	0.618	127.000	0.000
	L-group	0.503	24.000	0.000		L-group	0.618	24.000	0.000
	H-group	0.721	654.000	0.000		H-group	0.649	654.000	0.000
b_rotR	N-group	0.807	127.000	0.000	vsa_pol	N-group	0.912	127.000	0.000
	L-group	0.976	24.000	0.804		L-group	0.809	24.000	0.000
	H-group	0.962	654.000	0.000		H-group	0.895	654.000	0.000
Weight	N-group	0.897	127.000	0.000	SlogP	N-group	0.875	127.000	0.000
	L-group	0.984	24.000	0.955		L-group	0.977	24.000	0.837
	H-group	0.963	654.000	0.000		H-group	0.982	654.000	0.000
a_nN	N-group	0.808	127.000	0.000	ASA_A	N-group	0.987	127.000	0.289
	L-group	0.771	24.000	0.000		L-group	0.838	24.000	0.001
	H-group	0.772	654.000	0.000		H-group	0.996	654.000	0.081
a_nO	N-group	0.893	127.000	0.000	ASA_H	N-group	0.760	127.000	0.000
	L-group	0.760	24.000	0.000		L-group	0.948	24.000	0.240
	H-group	0.908	654.000	0.000		H-group	0.944	654.000	0.000
Q_VSA_FHYD	N-group	0.967	127.000	0.003	ASA_P	N-group	0.939	127.000	0.000
	L-group	0.973	24.000	0.743		L-group	0.955	24.000	0.352
	H-group	0.958	654.000	0.000		H-group	0.945	654.000	0.000
Q_VSA_FNEG	N-group	0.895	127.000	0.000	DASA	N-group	0.900	127.000	0.000
	L-group	0.862	24.000	0.004		L-group	0.967	24.000	0.583
	H-group	0.954	654.000	0.000		H-group	0.910	654.000	0.000
Q_VSA_FPOL	N-group	0.967	127.000	0.003	DCASA	N-group	0.818	127.000	0.000
	L-group	0.973	24.000	0.743		L-group	0.955	24.000	0.346
	H-group	0.958	654.000	0.000		H-group	0.828	654.000	0.000
Q_VSA_NEG	N-group	0.983	127.000	0.125	FASA_A	N-group	0.952	127.000	0.000
	L-group	0.797	24.000	0.000		L-group	0.944	24.000	0.195
	H-group	0.977	654.000	0.000		H-group	0.989	654.000	0.000

Q_VSA_POL	N-group	0.947	127.000	0.000	FASA	N-group	0.958	127.000	0.001
	L-group	0.890	24.000	0.013		L-group	0.881	24.000	0.009
	H-group	0.942	654.000	0.000		H-group	0.974	654.000	0.000
Q_VSA_POS	N-group	0.740	127.000	0.000	FASA_H	N-group	0.961	127.000	0.001
	L-group	0.972	24.000	0.721		L-group	0.976	24.000	0.812
	H-group	0.929	654.000	0.000		H-group	0.965	654.000	0.000
lip_acc	N-group	0.932	127.000	0.000	FASA_P	N-group	0.961	127.000	0.001
	L-group	0.777	24.000	0.000		L-group	0.976	24.000	0.812
	H-group	0.943	654.000	0.000		H-group	0.965	654.000	0.000
lip_don	N-group	0.805	127.000	0.000	FCASA_A	N-group	0.989	127.000	0.375
	L-group	0.846	24.000	0.002		L-group	0.916	24.000	0.047
	H-group	0.772	654.000	0.000		H-group	0.994	654.000	0.007
KierFlex	N-group	0.680	127.000	0.000	FCASA	N-group	0.991	127.000	0.624
	L-group	0.913	24.000	0.042		L-group	0.866	24.000	0.004
	H-group	0.962	654.000	0.000		H-group	0.993	654.000	0.002
logS	N-group	0.864	127.000	0.000	VSA	N-group	0.794	127.000	0.000
	L-group	0.917	24.000	0.050		L-group	0.989	24.000	0.994
	H-group	0.972	654.000	0.000		H-group	0.967	654.000	0.000
dipole	N-group	0.462	127.000	0.000	TPSA	N-group	0.949	127.000	0.000
	L-group	0.898	24.000	0.020		L-group	0.849	24.000	0.002
	H-group	0.363	654.000	0.000		H-group	0.959	654.000	0.000
a_acc	N-group	0.874	127.000	0.000	dens	N-group	0.896	127.000	0.000
	L-group	0.659	24.000	0.000		L-group	0.895	24.000	0.017
	H-group	0.897	654.000	0.000		H-group	0.955	654.000	0.000
a_don	N-group	0.794	127.000	0.000	glob	N-group	0.764	127.000	0.000
	L-group	0.722	24.000	0.000		L-group	0.788	24.000	0.000
	H-group	0.741	654.000	0.000		H-group	0.722	654.000	0.000
					logPow	N-group	0.873	127.000	0.000
						L-group	0.962	24.000	0.484
						H-group	0.991	654.000	0.000

Supplementary Table 4 Results of Kruskal-Wallis test

Test Statistics(a,b)

Descriptor	diameter	b_double	b_rotR	Weight	a_nN	a_nO	Q_VSA_FHYD	Q_VSA_FNEG
Chi-Square	55.111	44.237	14.967	69.175	27.021	8.321	10.818	88.823
df	2.000	2.000	2.000	2.000	2.000	2.000	2.000	2.000
Asymp. Sig.	0.000	0.000	0.001	0.000	0.000	0.016	0.004	0.000

Descriptor	Q_VSA_FPOL	Q_VSA_NEG	Q_VSA_POL	Q_VSA_POS	lip_acc	lip_don	KierFlex	logS
Chi-Square	10.818	85.882	22.254	33.091	6.030	16.751	46.444	110.411
df	2.000	2.000	2.000	2.000	2.000	2.000	2.000	2.000
Asymp. Sig.	0.004	0.000	0.000	0.000	0.049	0.000	0.000	0.000

Descriptor	dipole	a_acc	a_don	vsa_acc	vsa_don	vsa_pol	SlogP	ASA_A
Chi-Square	20.277	9.987	2.466	20.238	47.116	3.093	102.268	77.188
df	2.000	2.000	2.000	2.000	2.000	2.000	2.000	2.000
Asymp. Sig.	0.000	0.007	0.291	0.000	0.000	0.213	0.000	0.000

Descriptor	ASA_H	ASA_P	DASA	DCASA	FASA_A	FASA	FASA_H	FASA_P
Chi-Square	50.183	31.548	2.552	5.361	15.877	38.018	7.662	7.662
df	2.000	2.000	2.000	2.000	2.000	2.000	2.000	2.000
Asymp. Sig.	0.000	0.000	0.279	0.069	0.000	0.000	0.022	0.022

Descriptor	FCASA_A	FCASA	VSA	TPSA	dens	glob	logPow
Chi-Square	13.926	56.337	58.816	0.443	27.614	9.172	94.733
df	2.000	2.000	2.000	2.000	2.000	2.000	2.000
Asymp. Sig.	0.001	0.000	0.000	0.801	0.000	0.010	0.000

a Kruskal Wallis Test. Variables were considered to have a statistically significant difference if $p < 0.01$

b Grouping Variable: Group

Supplementary Table 5 Contribution of each principal component to variance

	PC1	PC2	PC3	PC4	PC5
% of variance	72.367	19.845	5.833	1.044	0.910
Cumulative %	72.367	92.213	98.046	99.090	100.000

Extraction Method: Principal Component Analysis.

Supplementary Table 6 Phenotypic group classification of nine selected EP probes

Name	FNEG	logS	SlogP	Predicted group	Experimentally observed group
**CS-2**	0.338	-3.780	3.416	L-group	L-group
**CS-3**	0.284	-4.150	3.588	L-group	L-group
**CS-8**	0.300	-3.092	2.898	L-group	L-group
**CS-11**	0.344	-2.649	2.391	L-group	L-group
**CS-13**	0.277	-2.483	1.412	L-group	L-group
**CS-14**	0.250	-2.626	1.428	L-group	N-group
**CS-42**	0.276	-2.442	1.650	L-group	L-group
**CS-56**	0.265	-3.416	2.931	L-group	L-group
**CS-101**	0.339	-2.757	2.434	L-group	L-group

Supplementary Table 7 Decision logic table illustrating the effect of molecular descriptors on cell permeability and degree of nonspecific binding of the probes

SlogP	Q_VSA_FNEG	logS	Prediction	Remark
1 to 4	0.15 to 0.35	-6 to -2	L-group	
	0.15 to 0.35	< -6	Grey-group*	If logS close to -6 tend to be in L-group
	> 0.35	-6 to -2 or < -6	Grey group*	If Q_VSA_FNEG > 0.53 tend to be in N-group, otherwise in H-group
> 4	> 0.35	-6 to -2	H-group	
	0.15 to 0.35	< -6	H-group	Tend to be in grey group
	> 0.35	< -6	H-group	Slight possibility to be in grey group
> 12 (Extreme high)	any range	< -12 (Extreme low)	N-group	
< 1 (Extreme low)	any range	> -2 (Extreme high)	N-group	

*Grey group is a group where all the three possible cellular behaviors might be found.

Supplementary Table 8 Molecular descriptor values, predicted phenotypic group and observed phenotypic group of BOR-1, BOR-2, BOR-1H and BOR-2H.

Probe name	BOR-1	BOR-2	BOR-1H	BOR-2H
**SlogP**	2.12	2.17	2.69	3.67
**FNEG**	0.31	0.32	0.49	0.42
**LogS**	-4.37	-4.8	-5.61	-6.59
**Predicted group[a]**	L-group	L-group	H-group	H-group
**Observed group[b]**	L-group	L-group	H-group	H-group

a: phenotypic groups were predicted based on molecular descriptor value of the probes
b: phenotypic groups were observed from cellular retention and efflux experiments in U-2 OS and CHO cell lines

Supplementary Table 9. Spectrocopic properties and purity table for CO-1 and CO-1H.

Calculated mass, experimental mass, absorbance maximum (λ_{abs}), fluorescent emission maximum (λem), extinction coefficient (ε), quantum yield (Φ), and purity.

Name	mass (calc)	m/z (exp)	λabs (nm)	λem (nm)	ε (1/M.cm)	QY (Φ)[b]	Purity (%)[c]
**CO-1**	494.24[a]	494.24[a]	490	510	66668	0.66	98
**CO-1H**	692.31[a]	692.31[a]	500	520	83333	0.83	98

Absorbance and fluorescence emission data were recorded by a Synergy 4, Biotek Inc. fluorescent plate reader at 10 µM concentration in DMSO in 96-well plates. HRMS *a*: found mass (M-H), *b*: tetramethyl BODIPY was used as standard, *c*: purity data was calculated on the basis of the integration in HPLC trace at 254 nm.

Supplementary Table 10 Decoding table for BDMCA, BDNCA and NYBD,

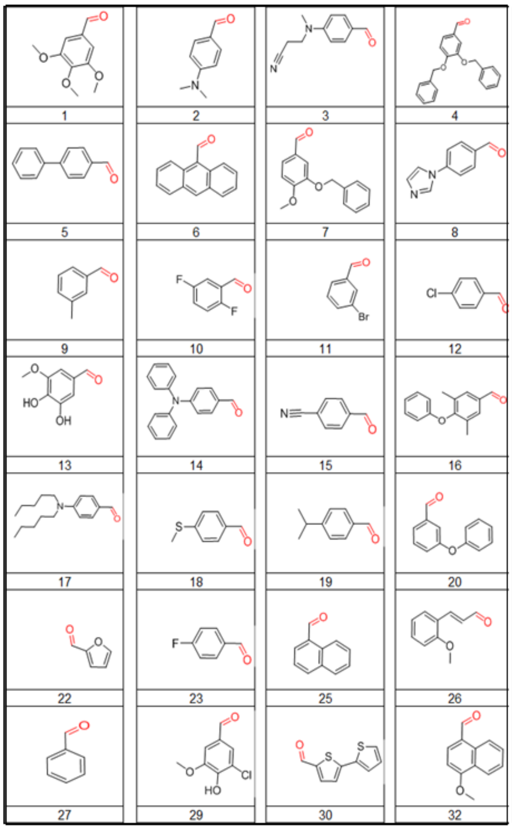

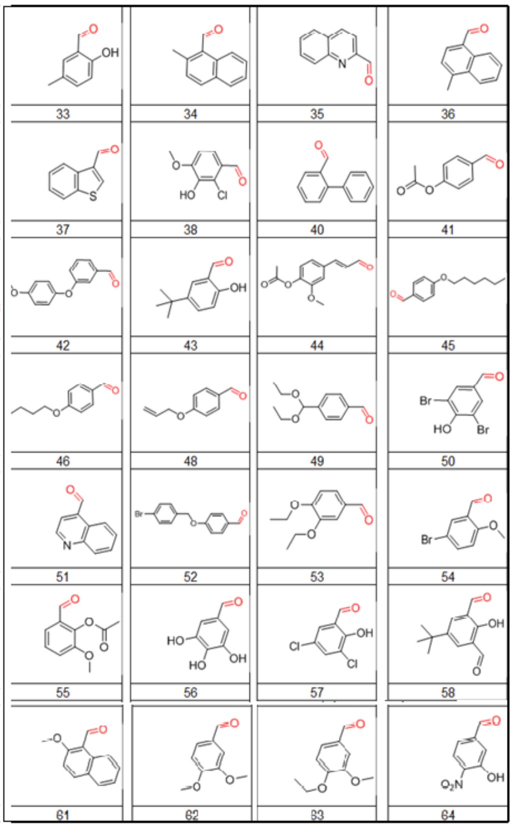

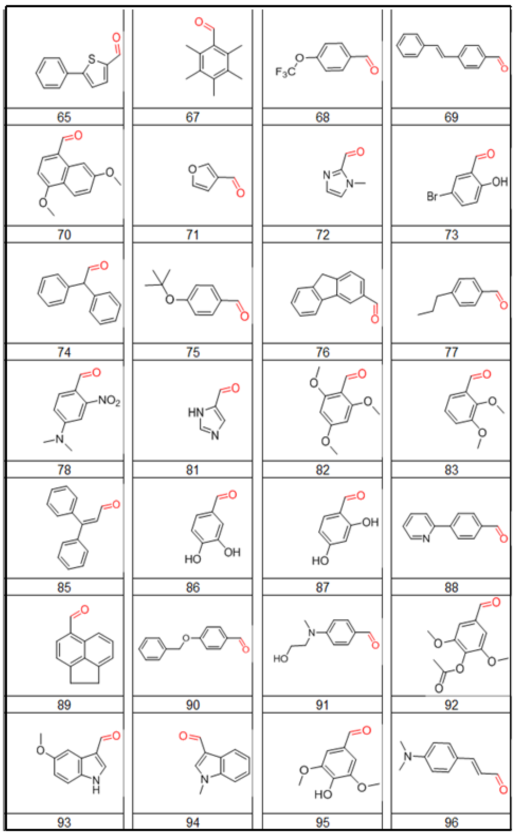

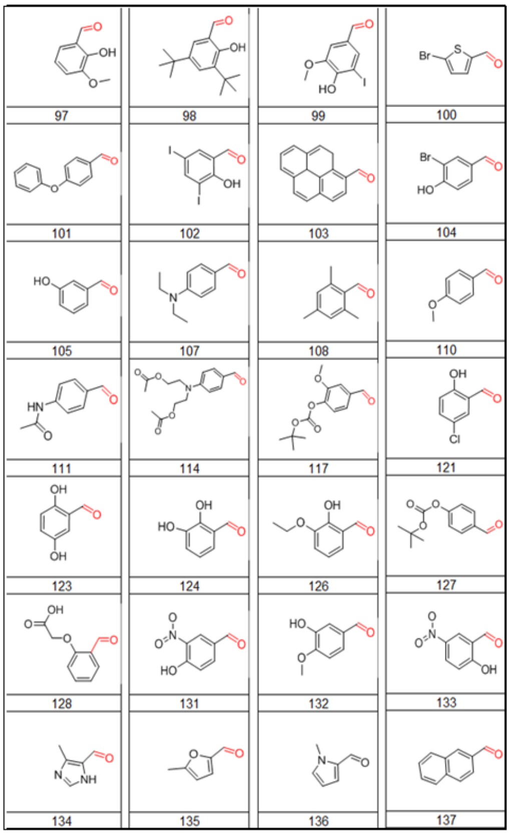

(Cont'd) Supplementary Table 10 Decoding table for BDMCA, BDNCA and NYBD.

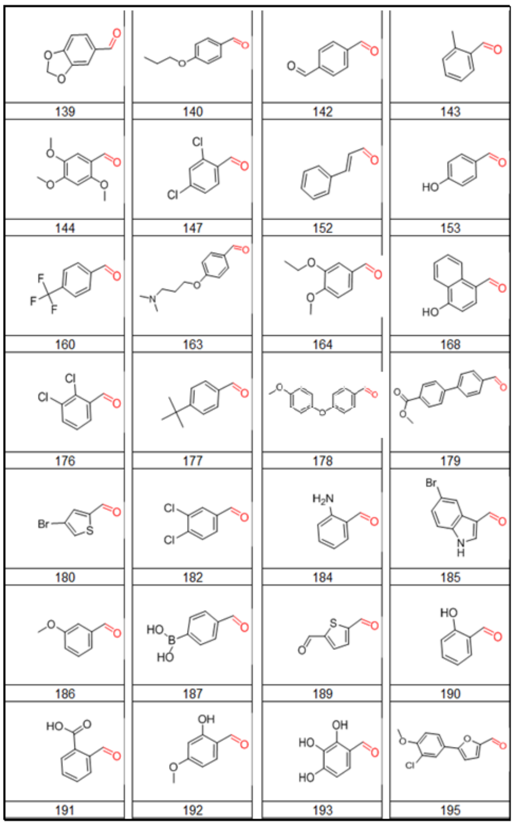

139	140	142	143
144	147	152	153
160	163	164	168
176	177	178	179
180	182	184	185
186	187	189	190
191	192	193	195

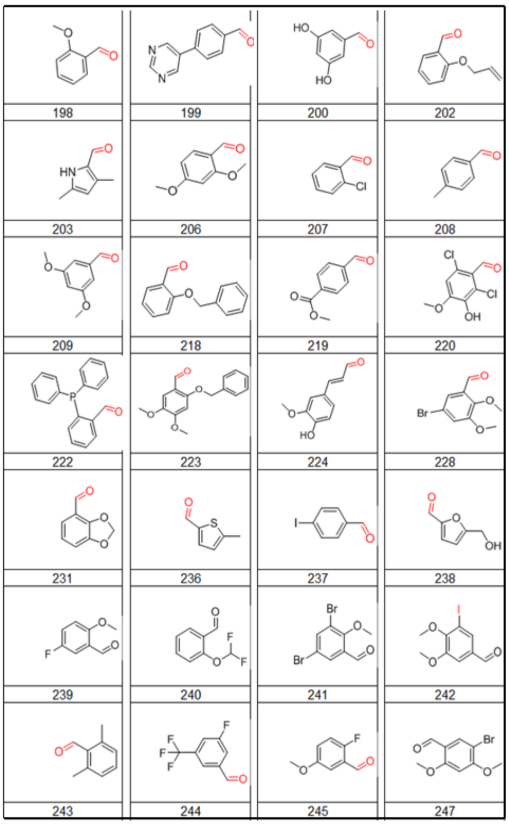

198	199	200	202
203	206	207	208
209	218	219	220
222	223	224	228
231	236	237	238
239	240	241	242
243	244	245	247

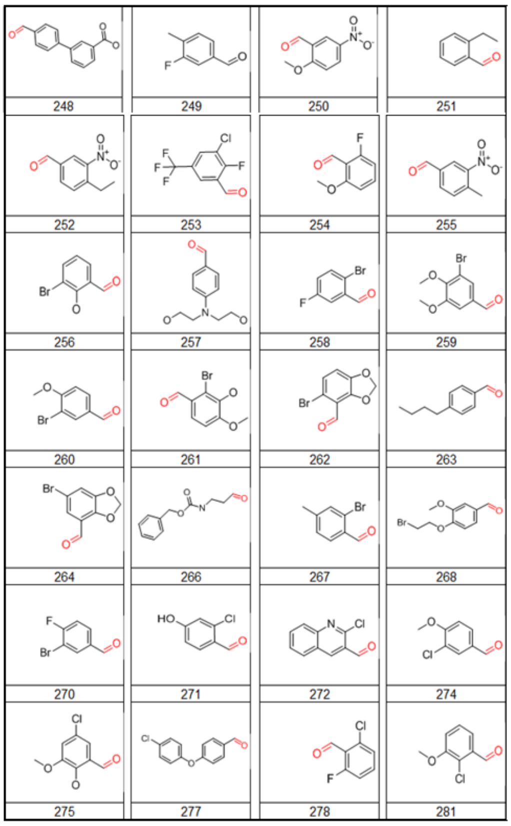

248	249	250	251
252	253	254	255
256	257	258	259
260	261	262	263
264	266	267	268
270	271	272	274
275	277	278	281

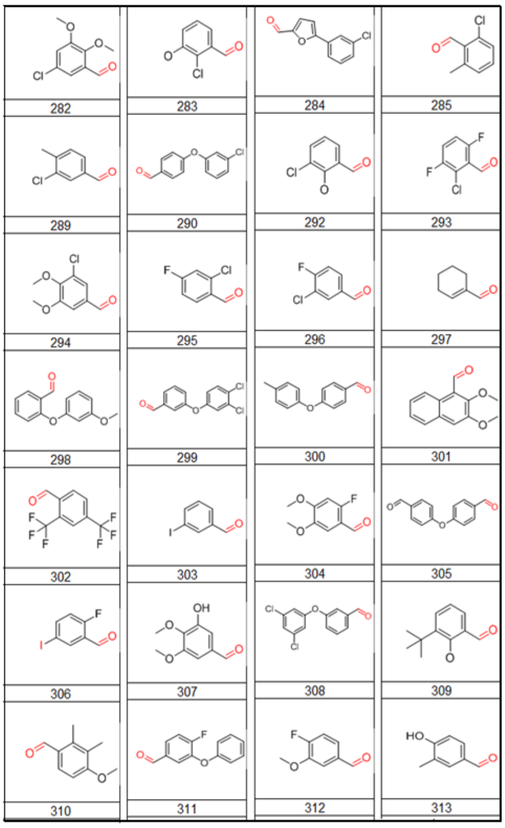

282	283	284	285
289	290	292	293
294	295	296	297
298	299	300	301
302	303	304	305
306	307	308	309
310	311	312	313

(Cont'd) Supplementary Table 10 Decoding table for BDMCA, BDNCA and NYBD.

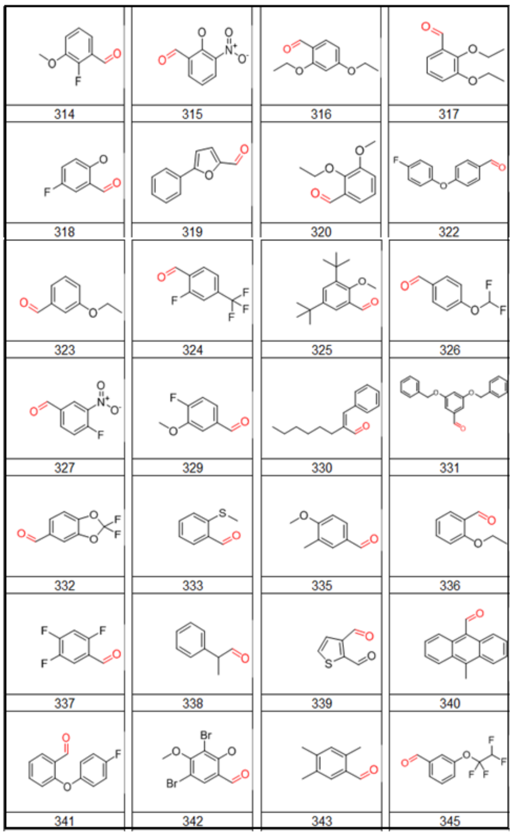

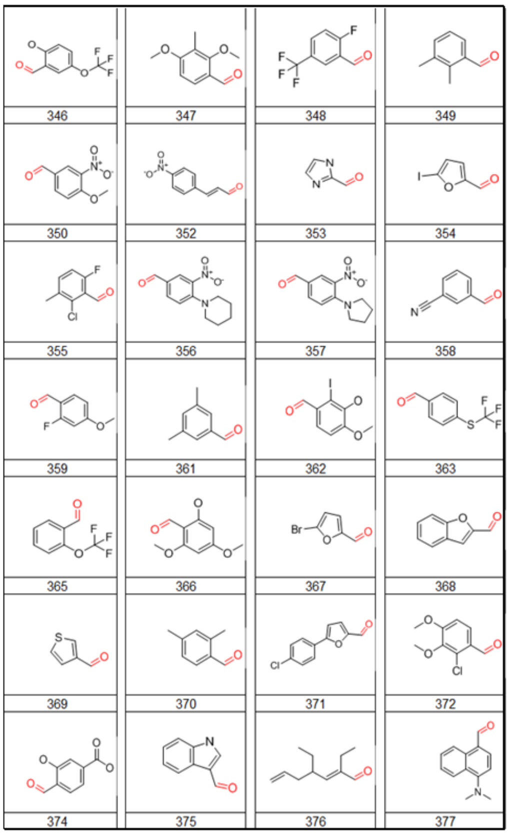

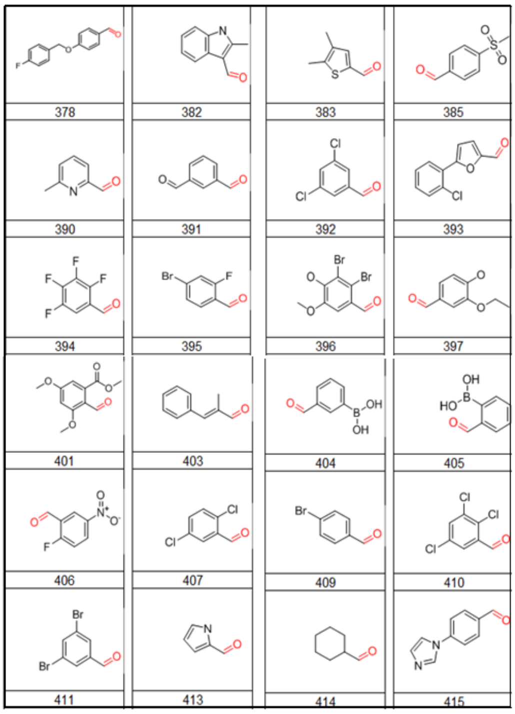

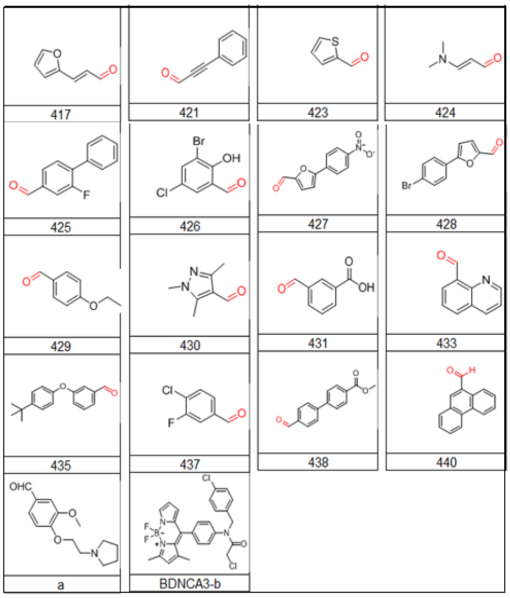

Supplementary Table 11 Decoding table for TAM-1, TAM-1’, TAM-2, TAM-3.

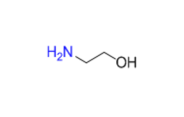	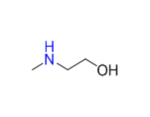	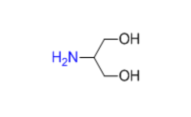	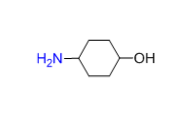	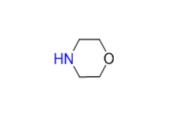
1	2	3	4	5
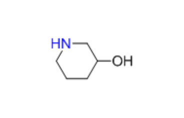	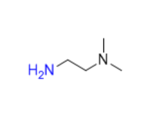	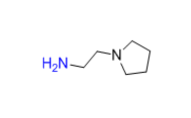	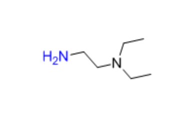	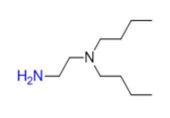
6	7	8	9	10
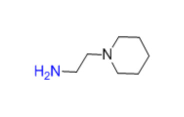	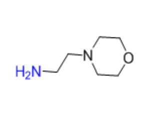	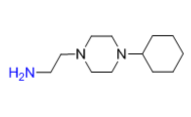	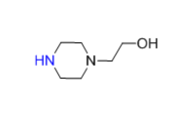	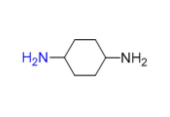
11	12	13	14	15
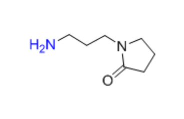	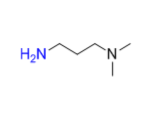	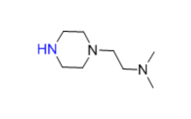	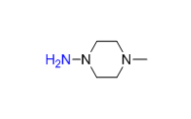	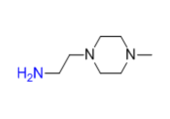
16	17	18	19	20
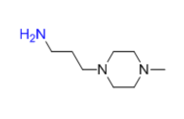	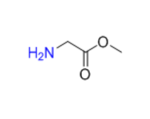	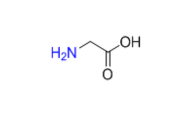	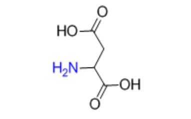	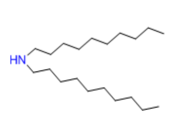
21	22	23	24	25
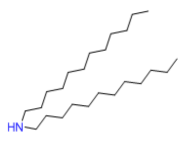	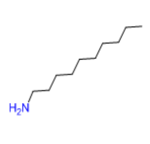	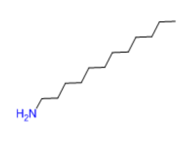	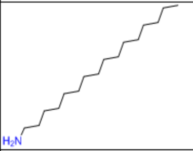	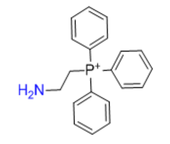
26	27	28	29	30

Supplementary Methods

Reagent- All the chemicals and solvents were purchased from Sigma Aldrich, Alfa Aesar, Fluka, MERCK, Tocris or Acros, and used without further purification. Normal phase purifications were carried out using Merck Silica Gel 60 (particle size: 0.040-0.063 mm, 230-400 mesh). Analytical characterization was performed on a HPLC-MS (Agilent-1200 series) with a DAD detector and a single quadrupole mass spectrometer (6130 series) with an ESI probe. ^{1}H-NMR and ^{13}C-NMR spectra were recorded on Bruker Avance 300 MHz NMR spectrometers, and chemical shifts are expressed in parts per million (ppm) and coupling constants are reported as a J value in Hertz (Hz). High resolution mass spectrometry (HRMS) data was recorded on a Micro mass VG 7035 (Mass Spectrometry Laboratory at National University of Singapore (NUS)).Spectroscopic and quantum yield data were measured on spectroscopic measurements, performed on a fluorometer and UV/VIS instrument, Synergy 4 of Bioteck Company. The slit width was 1 nm for both excitation and emission, and the data analysis was performed using GraphPrism 5.0.

Synthesis of CO-1

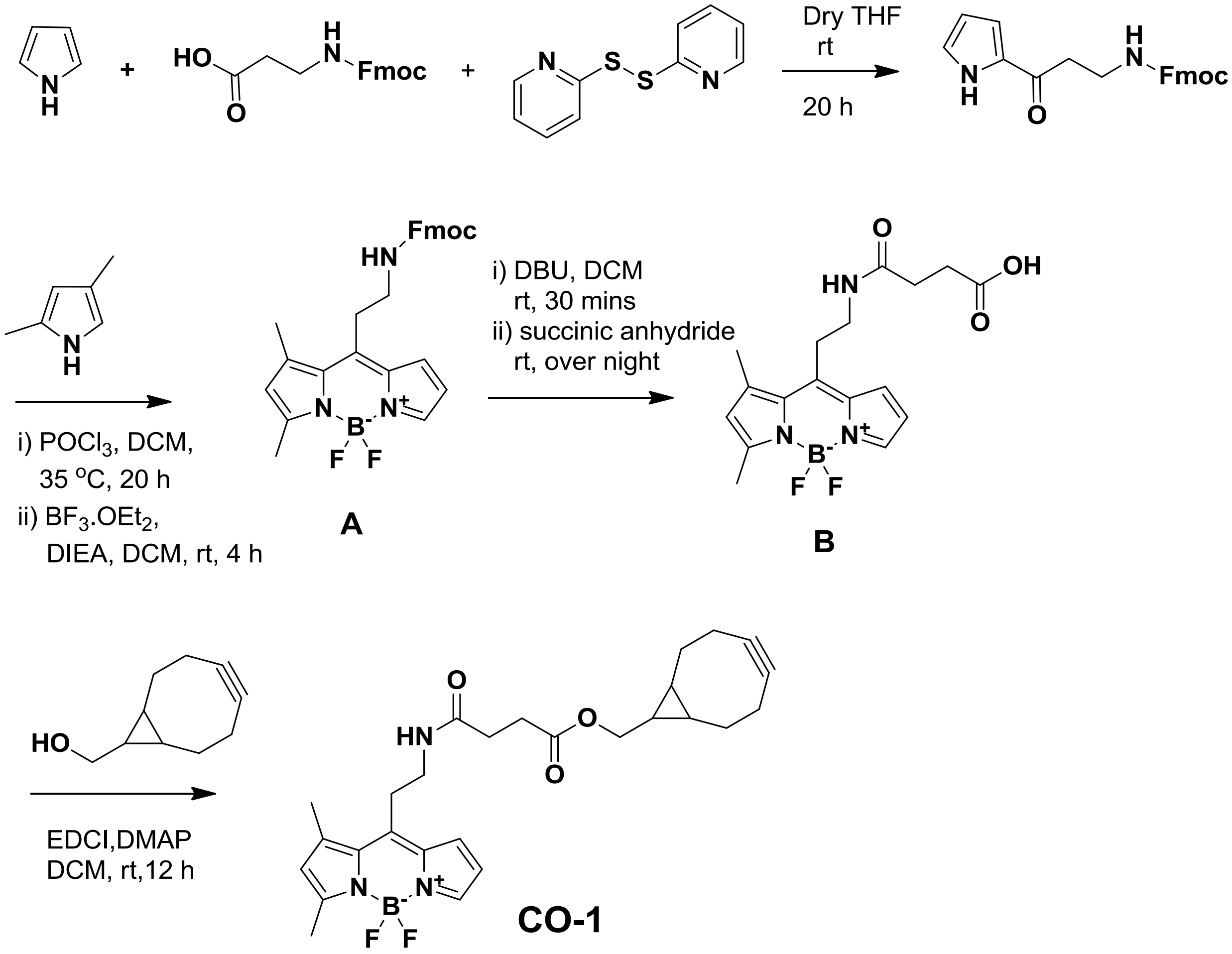

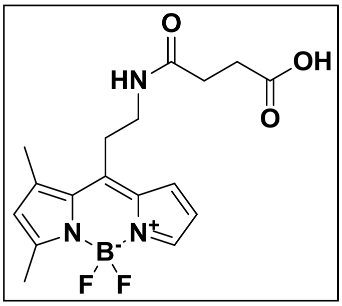

*Compound **B** (10-(2-(3-carboxypropanamido)ethyl)-5,5-difluoro-7,9-dimethyl-5H-dipyrrolo[1,2-c:2',1'-f][1,3,2]diazaborinin-4-ium-5-uide):*

Compound **A** was prepared according to the reported procedure[6]. Compound **A** (20 mg, 0.04 mmol) was dissolved in DCM (1 mL). To it, DBU (6.27 mg, 6.1 µL, 0.04 umol) was added drop wise and the reaction was stirred for 30 mins in rt. To the reaction mixture, succinic anhydride (8mg, 0.08 mmol) was added and stirred over night in rt. The reaction mixture was evaporated and crude product was purified by column chromatography (MeOH:DCM = 1:10). Product was obtained as red solid (10.9 mg, 75.4%). ^{1}H NMR ($CDCl_3$,

300 MHz): δ 7.61 (s, 1H), 7.12 (d, j= 3 Hz, 1H), 6.46 (d, j= 3 Hz, 1H), 6.19 (s, 1H), 3.19 (t, j= 6 Hz, 2H), 2.68-2.63 (m, 4H), 2.58 (s, 3H), 2.46 (s, 3H), 2.43 (t, j= 6 Hz, 2H). EI-MS (m/z): Calcd for $C_{17}H_{20}BF_2N_3O_3$ 363.15; found 362.1 (M-H).

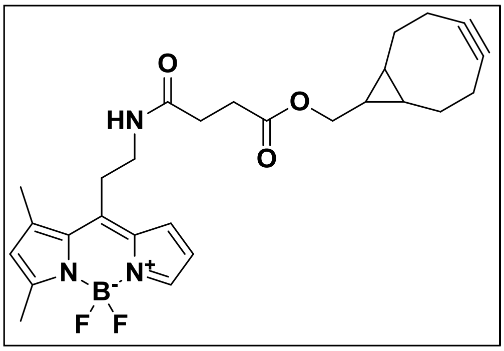

*Synthesis and characterization of **CO-1**:*

Compound **B** (5 mg, 0.013 mmol), bicyclo[6.1.0]non-4-yn-9-ylmethanol (2.47 mg, 0.016 mmol), EDCI (5.25 mg, 0.027 mmol) and DMAP (0.8 mg, 0.006 mmol) were dissolved together in DCM (0.2 mL) and stirred 12h in rt. Water (0.5 mL) was added to the reaction mixture and the organic layer was extracted in DCM. The crude was purified by column chromatography (MeOH:DCM = 0.5 : 10). Product was obtained as red solid (5.1 mg, 76.3%). ^{1}H NMR ($CDCl_3$, 300 MHz): δ 7.61 (s, 1H), 7.15 (d, j= 3 Hz, 1H), 6.45 (dd, j= 6 Hz, j= 3 Hz, 1H), 6.19 (s, 1H), 4.19 (d, j= 9 Hz, 2H), 3.19 (t, j= 7.5 Hz, 2H), 2.66 (m, 2H), 2.58 (s, 3H), 2.47 (s, 3H), 2.43 (t, j= 6 Hz, 2H), 2.30-2.22 (m, 4 H), 1.61-1.52 (m, 2H), 1.31—1.25 (m, 3H), 0.90-0.85 (m, 2H). ^{13}C NMR (75 MHz, $CDCl_3$) δ 173.00, 171.79, 161.18, 145.16, 142.56, 138.27, 134.08, 133.92, 124.21, 123.77, 116.04, 98.76, 62.80, 41.38, 30.93, 29.61, 29.37, 29.03, 21.37, 20.18, 17.37, 16.21, 15.01. HRMS: m/z calcd for $C_{27}H_{31}BF_2N_3O_3$ $(M\text{-}H)^-$ 494.2437, found 494.2424. $\lambda_{abs}/\lambda_{em}$ = 495/510 nm and quantum yield =0.66 (Tetramethyl Bodipy as standard), extinction co-efficient of **CO-1** = 66,668 M^{-1} cm^{-1} measured in DMSO.

Synthesis of CO-1H

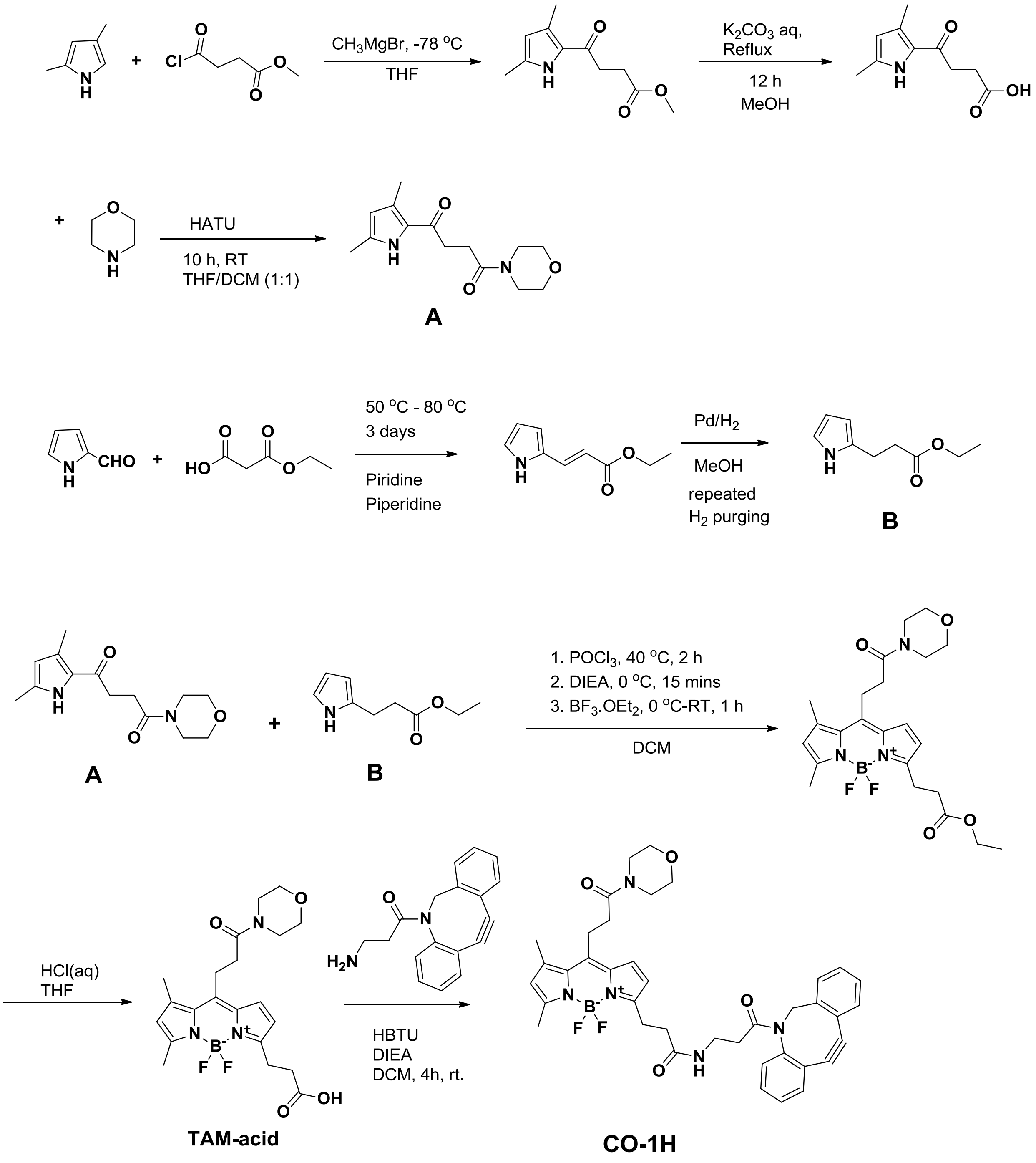

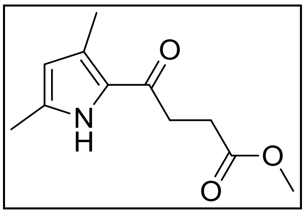

Methyl 4-(3,5-dimethyl-1H-pyrrol-2-yl)-4-oxobutanoate:

2,4-dimethyl-1H-pyrrole (3.16 g, 3.42 mL, 33.21 mmole) was dissolved in dry THF (120 mL) and cooled to -78° C under nitrogen atmosphere. CH_3MgBr (3 M in THF, 7.2 mL, 39.85 mmol) was added drop wise and the reaction mixture was stirred at -78° C for 30 mins. Temperature of the reaction mixture was raised to -20° C and stirred for another 30 mins. Methyl 4-chloro-4-oxobutanoate (7.9 g, 6.54 mL, 53.13 mmol) was added to the reaction mixture and stirred at -78 ^{0}C for 30 mins and at room temperature for 30 mins. The reaction mixture was quenched by the addition of saturated NH_4Cl solution at 0° C. Organic layer was extracted after repeated washing the mixture in water and ethyl acetate. Organic layer was dried over Na_2SO_4 and evaporated over rotary evaporator. Crude was purified by column chromatography (EA: Hexane = 1: 5) to obtain pure product as a white floppy solid (3.2 g, 46%). ^{1}H NMR (300 MHz, $CDCl_3$) δ 9.54 (s, 1H), 5.81 (s, 1H), 3.68 (s, 3H), 3.04 (t, J = 6.7 Hz, 2H), 2.73 (t, J = 6.7 Hz, 2H), 2.36 (s, 3H), 2.25 (s, 3H). EI-MS (m/z): Calcd for $C_{11}H_{15}NO_3$ 209.1; found 210.1 (M+H).

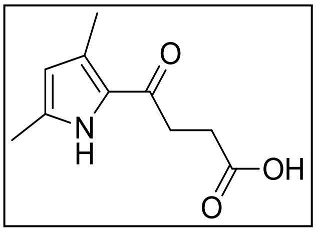

4-(3,5-dimethyl-1H-pyrrol-2-yl)-4-oxobutanoic acid:

Methyl 4-(3,5-dimethyl-1H-pyrrol-2-yl)-4-oxobutanoate (1 g, 4.78 mmol) was dissolved in ethanol (40 mL). K_2CO_3 (1. 32 g, 9.57 mmol), dissolved in water (15 mL) was added to it. The reaction mixture was heated to reflux overnight. Then the solution was cooled to room temperature. The solvent was removed in rotary evaporator. Water (20 mL) was added and the solution was acidified (pH= 2) upon addition of 10% HCl solution. The mixture was filtered and dried over vacuum to obtain product as gray powdered solid (0.7 g, 75 %). ^{1}H NMR (300 MHz, DMSO-d6) δ 12.03 (s, 1H), 11.19 (s, 1H), 5.77 (s, 1H), 2.91 (t, J = 6.5 Hz, 2H), 2.52 (t, J = 6.5 Hz, 2H), 2.26 (s, 3H), 2.17 (s, 3H). ^{13}C

NMR (75 MHz, DMSO-d6) δ 187.04, 174.44, 134.04, 127.81, 112.08, 28.17, 14.33, 12.91. EI-MS (m/z): Calculated for $C_{10}H_{13}NO_3$ 195.0; found 194.0 (M-H).

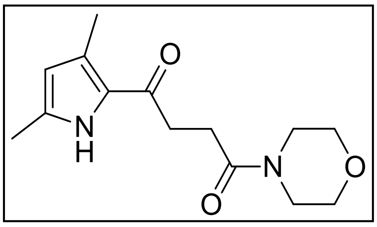

1-(3,5-dimethyl-1H-pyrrol-2-yl)-4-morpholino butane-1,4-dione (A):

4-(3, 5-dimethyl-1H-pyrrol-2-yl)-4-oxobutanoic acid (0.6 g, 3.07 mmol), morpholine (0.53 g, 6.1 mmol), HBTU (1.4 g, 3.68 mmol) were dissolved in dry THF. DIEA (1.6 mL, 9.21 mmol) was added to it and the reaction mixture was stirred for 4 hours under nitrogen atmosphere. Then the solvent was evaporated in rota vapour. Crude was dissolved in ethyl acetate and washed with water two times. Organic layer was dried over Na_2SO_4 and evaporated over rotary evaporator. Crude was purified by column chromatography (EA: Hexane = 1: 4) to obtain pure product as a yellowish white solid (0.62 g, 78%). ^{1}H NMR (300 MHz, $CDCl_3$) δ 9.42 (s, 1H), 5.80 (s, 1H), 3.66-3.55 (m, 8H), 3.08 (t, J = 6.6 Hz, 2H), 2.72 (t, J = 6.3 Hz, 2H), 2.36 (s, 3H), 2.23 (s, 3H). ^{13}C NMR (75 MHz, $CDCl_3$) δ 187.55, 170.81, 128.01, 112.70, 77.41, 76.98, 76.56, 66.77, 66.51, 45.81, 42.05, 34.11, 26.90, 14.45, 12.91. EI-MS (m/z): Calcd for $C_{14}H_{20}N_2O_3$ 264.1; found 265.1 (M+H).

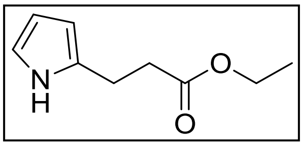

Ethyl 3-(1H-pyrrol-2-yl)propanoate:

This compound was synthesized as reported.[7]

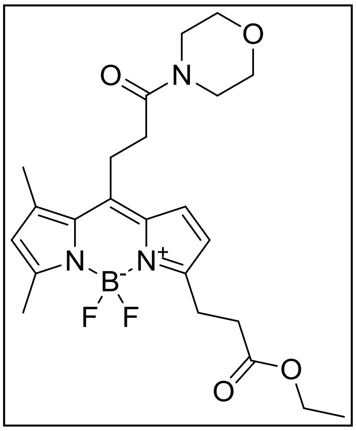

3-(3-ethoxy-3-oxopropyl)-5,5-difluoro-7,9-dimethyl-10-(3-morpholino-3-oxopropyl)-5H-dipyrrolo[1,2-c:2',1'-f][1,3,2]diazaborinin-4-ium-5-uide:

1-(3,5-dimethyl-1H-pyrrol-2-yl)-4-morpholinobutane-1,4-dione (A) (58 mg, 0.22 mmol) and ethyl 3-(1H-pyrrol-2-yl)propanoate (B) (37 mg, 0.22 mmol) were dissolved in DCM (1 mL). $POCl_3$ (67 mg, 41 µL, 0.44 mmol) was added dropwise to the reaction mixture in nitrogen atmosphere. The reaction mixture was stirred at 40° C for 2h. Then it was cooled to 0° C, DIEA (128 mg, 172 µL, 0.99 mmole) was added drop wise and stirred for 15 mins. $BF_3.OEt_3$ (140 mg, 122 µL, 0.99 mmole) was added to the reaction mixture at 0° C and stirred for 1h. Solvent was evaporated from the reaction mixture and crude was purified by column chromatography (EA: Hexane = 1: 1). Product was obtained as red solid (46 mg, 45.3%). ^{1}H NMR (300 MHz, $CDCl_3$) δ 7.03 (d, J = 4.1 Hz, 1H), 6.25 (d, J = 4.1 Hz, 1H), 6.11 (s, 1H), 4.14 (t, J = 7.5 Hz, 3H), 3.60 – 3.52 (m, 4H), 3.31 – 3.23 (m, 8H), 2.71 – 2.59 (m, 4H), 2.53 (s, 3H), 2.38 (s, 3H), 1.23 (t, J = 6 Hz, 3H). ^{13}C NMR (75 MHz, $CDCl_3$) δ 172.43, 169.27, 158.62, 156.04, 143.98, 142.79, 133.75, 132.31, 124.96, 122.81, 116.19, 66.29, 60.44, 45.75, 42.06, 38.52, 35.06, 33.38, 24.59, 23.72, 15.93, 14.70, 14.12. EI-MS (m/z): Calcd for $C_{23}H_{30}BF_2N_3O_4$ 461.2; found 460.1 (M-H).

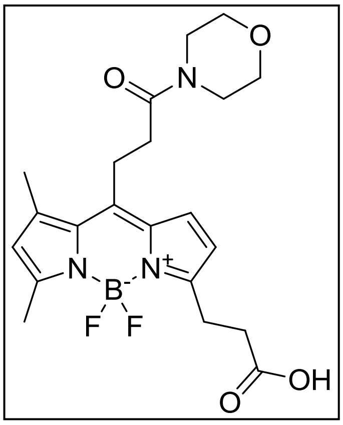

3-(2-carboxyethyl)-5,5-difluoro-7,9-dimethyl-10-(3-morpholino-3-oxopropyl)-5H-dipyrrolo[1,2-c:2',1'-f][1,3,2]diazaborinin-4-ium-5-uide:

3-(3-ethoxy-3-oxopropyl)-5,5-difluoro-7,9-dimethyl-10-(3-morpholino-3-oxopropyl)-5H-dipyrrolo[1,2-c:2',1'-f][1,3,2]diazaborinin-4-ium-5-uide (45 mg, 0.1 mmol) was dissolved in THF (6 mL). To it, mixture of 37% HCl (0.2 mL) and water (1 mL) was added of drop wise. The reaction mixture was stirred for 72 h in room temperature. The organic layer was poured in water (20 mL) followed by ethyl acetate (10 mL). Then the organic layer was extracted and washed several times with water to remove excess HCl in the reaction mixture. The organic layer was dried in Na_2SO_4 and dried in rotary evaporator. The crude was purified by column

chromatography (EA: Hexane = 9: 1). Product was obtained as red solid (17.7 mg, 41%). ^{1}H NMR (300 MHz, $CDCl_3$) δ 7.05 (d, J = 4.0 Hz, 1H), 6.30 (d, J = 4.1 Hz, 1H), 6.13 (s, 1H), 3.62 – 3.53 (m, 4H), 3.34-3.23 (m, 8H), 2.80 (t, J = 7.5 Hz, 2H), 2.68 – 2.60 (t, J = 7.5 Hz, 2H), 2.55 (s, 3H), 2.40 (s, 3H). EI-MS (m/z): Calcd for $C_{21}H_{26}BF_2N_3O_4$ 433.19; found 432.1 (M-H).

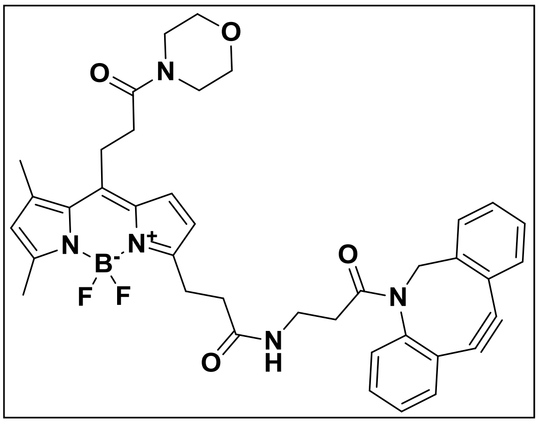

Synthesis and characterizations of CO-1H:

3-(2-carboxyethyl)-5,5-difluoro-7,9-dimethyl-10-(3-morpholino-3-oxopropyl)-5H-dipyrrolo[1,2-c:2',1'-f][1,3,2]diazaborinin-4-ium-5-uide (5 mg, 0.011 mmol), Dibenzocyclooctyne-amine (3.2 mg, 0.011 mmol), HBTU (5.2 mg, 0.014 mmol) and DIEA (1.8 mg, 2.4 μL, 0.014 mmol) were dissolved together in DCM (0.2 mL) and stirred 8h in rt. Water (0.5 mL) was added to the reaction mixture and the organic layer was extracted in DCM. The crude was purified by column chromatography (MeOH: DCM = 1: 5). Product was obtained as red solid (6.4 mg, 84.1%). ^{1}H NMR (300 MHz, $CDCl_3$) δ 7.61 (s, 1H), 7.14 (d, *J* = 3.8 Hz, 1H), 6.45 (dd, *J* = 4.0, 2.1 Hz, 1H), 6.18 (s, 1H), 5.99 (t, *J* = 6.1 Hz, 1H), 4.17 (d, *J* = 8.2 Hz, 2H), 3.55 (dd, *J* = 13.7, 6.9 Hz, 2H), 3.18 (t, *J* = 7.1 Hz, 2H), 2.66 (t, *J* = 6.7 Hz, 2H), 2.58 (s, 3H), 2.47 (s, 3H), 2.43 (t, *J* = 6.7 Hz, 2H), 2.25 – 2.21 (m, 4H), 1.62 – 1.52 (m, 4H), 0.99 – 0.88 (m, 3H). 13C NMR (75 MHz, CD3OD) δ 172.87, 171.74, 170.39, 158.33, 155.99, 151.07, 147.94, 144.07, 143.49, 131.93, 128.98, 128.56, 128.20, 127.73, 127.44, 126.65, 125.03, 122.79, 122.51, 122.16, 115.82, 114.17, 107.36, 106.20, 66.05, 60.05, 55.11, 45.81, 41.89, 35.21, 34.37, 34.19, 33.93, 29.23, 28.81, 24.35, 19.38, 14.60, 12.97. HRMS: m/z calcd for $C_{39}H_{40}BF_2N_5O_4$ $(M+H)^+$ 692.3141, found 692.3166.

Synthesis of TPP-Az

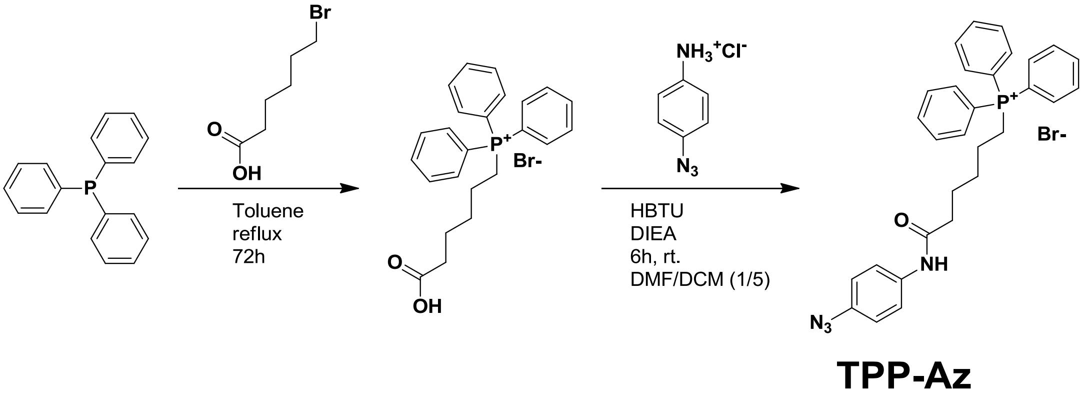

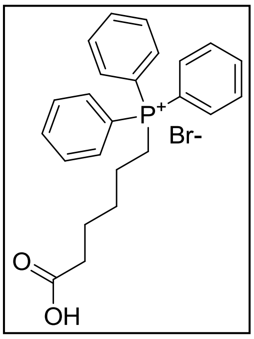

(5-carboxypentyl)triphenylphosphonium bromide:

Triphenylphosphine (20 mg, 0.076 mmol) and 6-bromohexanoic acid (14.8 mg, 0.076 mmol) were dissolved in dry toluene (0.2 mL). The reaction mixture was refluxed over 72h. The solution was concentrated. The residue was washed consecutively with benzene (3 × 1 mL), hexane (1 mL), and Et_2O (2 × 1 mL). The crystalline white solid was dried to give the pure product (28 mg, 97.5 %). ^{1}H NMR (300 MHz, $CDCl_3$) δ 7.80-7.68 (m, 15H), 3.58 (bs, 2H), 2.34-2.32 (m,2H), 1.63-1.57 (m, 6H). EI-MS (m/z): Calcd for $C_{24}H_{26}O_2P^+$ 377.16; found 377.1

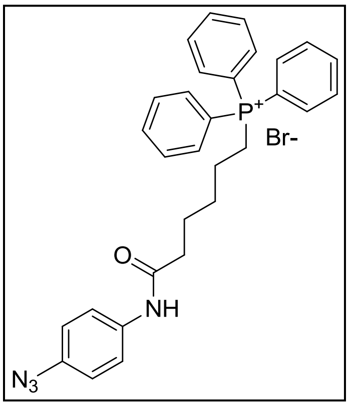

*(6-((4-azidophenyl)amino)-6-oxohexyl)triphenylphosphonium bromide (**TPP-Az**)*:

(5-carboxypentyl)triphenylphosphonium bromide (10 mg, 0.026 mmol), 4-azidobenzenaminium chloride (4.5 mg, 0.026 mmol), HBTU (20 mg, 0.052 mmol) were dissolved in mixture of solvents (dry DMF (0.05 mL) and DCM (0.1 mL). To the reaction mixture, 7 uL of DIEA was added and the reaction mixture was stirred overnight at rt. Solvent was evaporated and the crude was purified by column chromatography (EA: Hexane = 1: 2). Product was obtained as white solid (9.9 mg, 77.7 %). ^{1}H NMR (DMSO-d6, 300 MHz): 7.83-7.70 (m,

15H), 7.63 (d, j= 9Hz, 2H), 7.07 (d, j= 9 Hz, 2H), 3.62 (bs, 2H), 2.30-2.25 (m, 2H), 1.61-1.54 (m, 6H). ^{13}C NMR (75 MHz, DMSO-d6) δ 171.29, 136.94, 135.23, 133.99, 130.67, 120.85, 119.72, 119.47, 118.33, 36.14, 31.13, 24.64, 18.43, 17.08. HRMS: m/z calculated for $C_{30}H_{30}N_4OP^+$ 493.2152, found 493.2153

Synthesis of Morph-Az

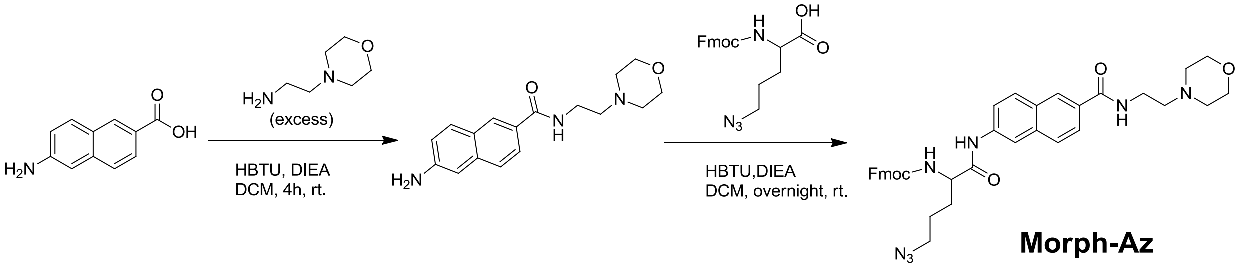

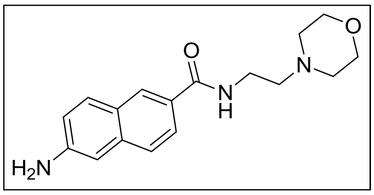

(6-amino-N-(2-morpholinoethyl)-2-naphthamide):

Compound **A** (6-amino-2-naphthoic acid, 25 mg, 0.13 mmol), 2-morpholinoethanamine (69.5 mg, 70 µL, 0.53 mmol), HBTU (98.5 mg, 0.26 mmol) were dissolved together in DCM. To the stirred solution, N,N-Diisopropylethylamine (16.77 mg, 22.6 µL, 0.13 mmol) was added drop wise. The reaction mixture was stirred in rt for 4 hours. The solvent was evaporated in rota vapour and the crude was obtained. The crude was purified by column chromatography using EA as eluent. Product was obtained as brown semi solid (21.24 mg, 54.6%). ^{1}H NMR ($CDCl_3$, 300 MHz): δ 7.21 (d, 1H, j= 3 Hz), 7.78-7.73 (m, 2H), 7.65 (d, j= 9 Hz, 1H), 7.73-7.00 (m, 2H), 3.79 (t, j= 4.5 Hz, 4H), 3.65 (q, j= 6.0 Hz, 2H), 2.68 (t, j= 6.0 Hz 2H), 2.58(t, j= 4.5 Hz, 4H). ^{13}C NMR (75 MHz, $CDCl_3$) δ 167.69, 145.89, 136.50, 130.38, 128.29, 127.52, 126.73, 125.94, 123.96, 118.81, 107.84, 66.81, 57.03, 53.27, 36.00. EI-MS (m/z): Calcd for $C_{17}H_{21}N_3O_2$, 299.16; found 300.0 (M+H).

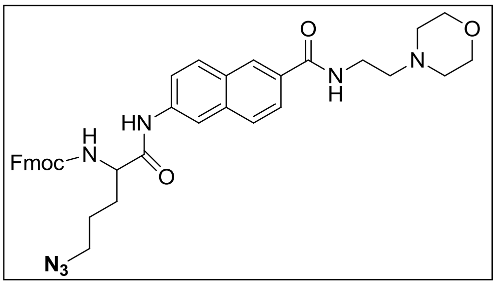

Synthesis of compound ***Morph-Az***

((9H-fluoren-9-yl)methyl(5-azido-1-((6-((2-morpholinoethyl)carbamoyl)naphthalen-2-yl)amino)-1-oxopentan-2-yl)carbamate):

Compound **B** (20 mg, 0.066 mmol), 2-((((9H-fluoren-9-yl)methoxy)carbonyl)amino)-5-azidopentanoic acid (38 mg, 0.01 mmol), HBTU (35 mg, 0.092 mmol) were dissolved together in DCM. To the stirred solution, N,N-Diisopropylethylamine (8.5 mg, 11.5 µL, 0.066 mmol) was added drop wise. The reaction mixture was stirred in rt overnight. The solvent was evaporated in rota vapour and the crude was obtained. The crude was purified by column chromatography using (MeOH:DCM = 1 : 10). Product was obtained as white solid (18.5 mg, 42.5%). ^{1}H NMR ($CDCl_3$, 300 MHz): δ 8.94 (s, 1H), 8.28 (s, 1H), 8.20 (s, 1H), 7.80-7.69 (m, 5H), 7.63-7.59 (m, 2H), 7.44-7.39 (m, 3H), 7.33-7.29 (m, 2H), 5.81 (d, j= 9 Hz, 1H), 4.53-4.49 (m, 2H), 4.26 (t, j= 7.5 Hz, 1H), 3.88 (t, j= 4.5 Hz, 4H), 3.77-3.73 (m, 2H), 3.42-3.35 (m, 2H), 2.92-2.85 (m, 2H), 2.84-2.74 (m, 4H), 2.07-1.65 (m, 4H). ^{13}C NMR (75 MHz, $CDCl_3$) δ. 171.69, 166.71, 156.47, 144.23, 144.13, 141.09, 138.40, 135.11, 130.00, 129.90, 129.14, 128.00, 127.73, 127.68, 127.59, 127.42,125.69, 125.06, 121.02, 120.47, 115.34, 66.12, 66.06, 55.54, 50.74, 47.06, 35.48, 31.62, 29.38, 26.96, 25.49, 22.43. HRMS: m/z calcd for $C_{37}H_{39}N_7O_5$ $(M+H)^-$ 661.3085, found 661.3046.

Synthesis of Sphingo-Az

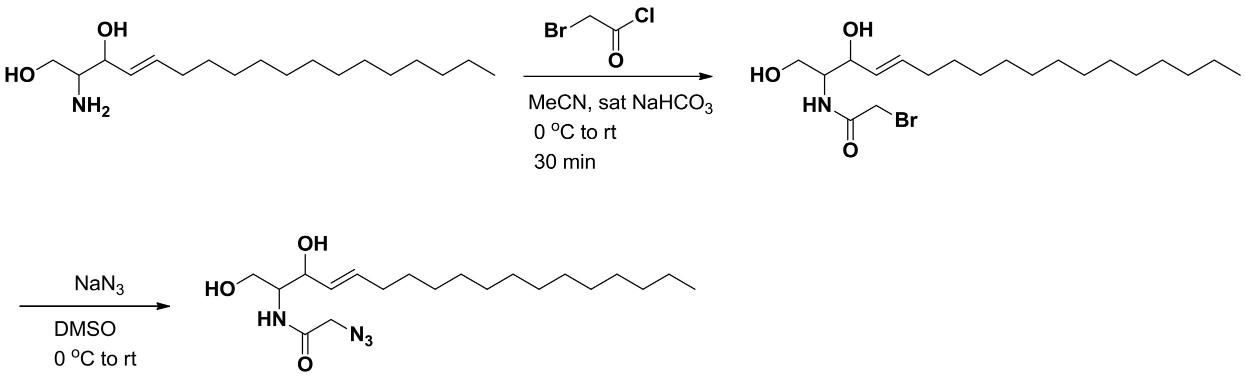

Sphingo-Az

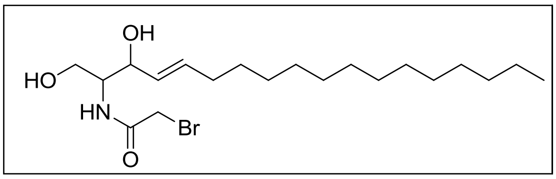

((E)-2-bromo-N-(1,3-dihydroxyoctadec-4-en-2-yl)acetamide):

Sphingosine (20 mg, 0.066 mmol) was dissolved in acetonitrile (30 mL). To this solution, 100 µL of saturated sodium bicarbonate solution was added. The solution was kept in ice bath and then bromoacetyl chloride (42 µL, 0.266 mmol) was added slowly and reaction was allowed to stir at room temperature for 30 min. The reaction was monitored by TLC using 20% Ethyl acetate/Hexane system. Upon completion of the reaction, solvent was removed under reduced pressure to dryness. Then around 20 mL of Ethyl acetate was added and then the organic layer was washed with water, brine and collected, dried over sodium sulphate and then evaporated to obtain 25 mg of white solid crude. Crude was directly carried forward for the next reaction.

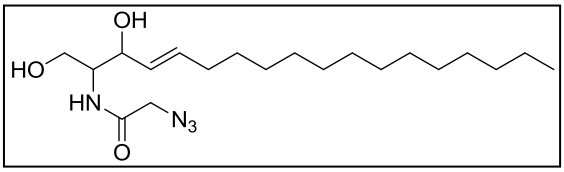

*((E)-2-azido-N-(1,3-dihydroxyoctadec-4-en-2-yl)acetamide) (**Sphingo-Az**)*:

Crude compound **2** (25 mg, 0.047 mmol) was dissolved in of DMSO (10 mL) and then sodium azide (15 mg, 0.23 mmol) was added to the solution. The reaction mixture was allowed to stir at room temperature for 4 hours. The reaction was monitored by TLC using 20% Ethylacetate/Hexane system. Upon completion of the

reaction, water (20 mL) was added slowly to the reaction mixture followed by Ethyl acetate (20 mL). The organic layer was extracted out and then dried over sodium sulphate and then evaporated to obtain white solid crude which was then purified by column chromatography (EA: Hexane = 1: 9). Product was obtained as white solid (12.1 mg, 67.5%). ^{1}H NMR (300 MHz, $CDCl_3$) δ 6.70 (d, *J* = 8.0 Hz, 1H), 5.87 – 5.70 (m, 1H), 5.49 (dd, *J* = 15.5, 6.2 Hz, 1H), 4.40 (d, *J* = 4.9 Hz, 1H), 4.29 – 4.24 (m, 2H), 4.00 (s, 1H), 3.89 (s, 1H), 2.09 – 2.02 (m, 2H), 1.36 – 1.25 (m, 22H), 0.87 (t, *J* = 6.7 Hz, 3H). ^{13}C NMR (75 MHz, $CDCl_3$) δ 166.92, 135.50, 127.63, 72.64, 63.74, 52.59, 52.46, 50.31, 31.83, 29.59, 29.51, 29.39, 29.26, 29.12, 28.98, 22.60, 14.02. HRMS: m/z calcd for $C_{20}H_{38}N_4O_3$ 405.2836 $(M+Na)^+$, found 405.2847.

Synthesis of BOR-1

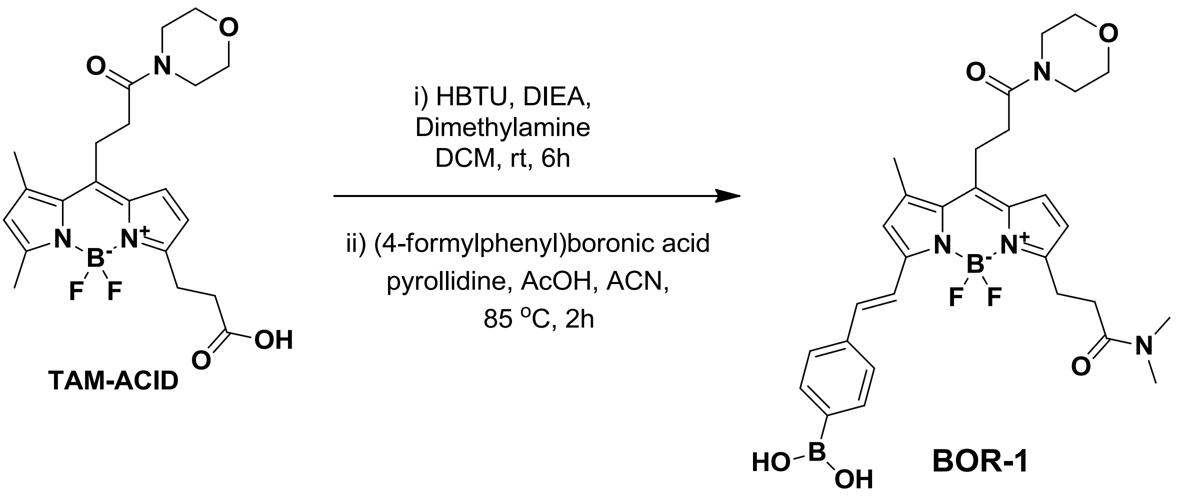

TAM-ACID (100 mg, 0.23 mmol), Dimethyl amine (31 µL, 0.46 mmol), HBTU (174.3 mg, 0.46 mmol) and DIEA (80 µL, 0.46 mmol)) were dissolved in dry DCM (4 mL). The solution was stirred 6h in rt. The reaction was monitored by TLC. Then the solvent was evaporated and amide product was purified in column chromatography. The product was taken to the next step. The amide product (40 mg, 0.086 mmol), (4-formylphenyl)boronic acid (28 µL, 0.347 mmol),were dissolved in ACN (1 mL). Acetic acid (30 µL, 0.516 mmol) and pyrrolidine (43 µL, 0.516 mmol) were added to the reaction mixture and the solution was stirred at 85 ^{o}C. TLC was monitored in every 10 mins interval. After 2 hours, the solution was evaporated and the product was purified in column chromatography to obtain BOR 1 as red solid (29 mg, 58.5%). ^{1}H NMR (d-acetone, 300 MHz): δ 7.95 (d, j = 9 Hz, 2H), 7.65 (d, j = 6 Hz, 2H), 7.35 (d, j = 3 Hz, 1H), 7.30 (s, 1H), 7.04 (s, 1H), 6.43 (d, j = 3 Hz, 1H), 5.78 (s, 1H), 3.57-3.51 (m, 4H), 3.33-3.23 (m, 8H), 2.63-2.56 (m, 2H), 2.52 (s, 3H), 2.16 (d, j = 9 Hz, 2H), 1.59 (m, 2H), 1.10 (t, j = 6Hz, 3H). HRMS (m/z): Calcd for $C_{30}H_{36}B_2F_2N_4O_5$ (-F) 573.2856; found 573.2842 (M-F).

Synthesis of BOR-2

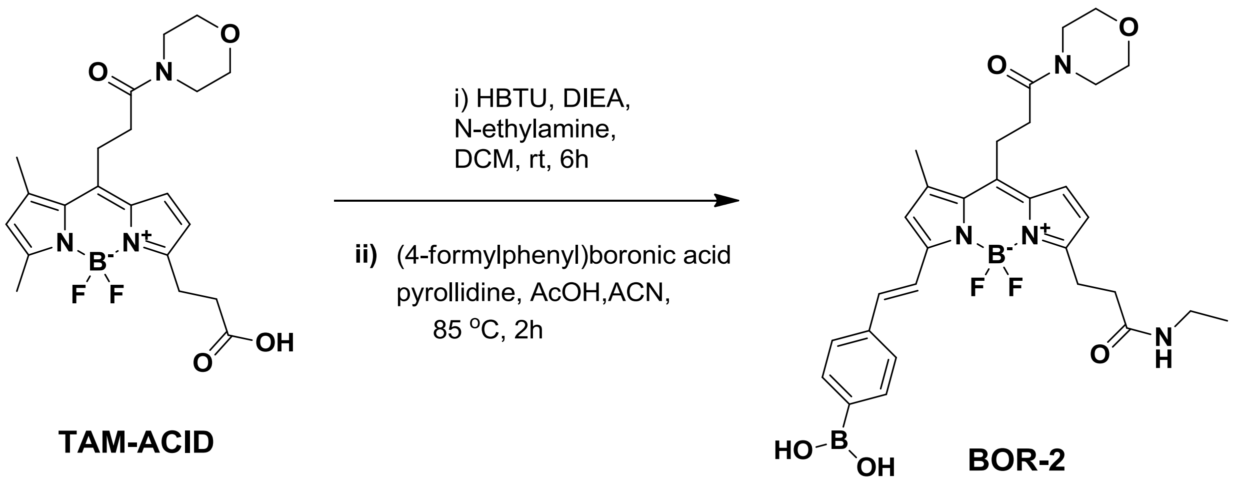

TAM-ACID (100 mg, 0.23 mmol), ethylamine hydrochloride (37.5 mg, 0.46 mmol), HBTU (174.3 mg, 0.46 mmol) and DIEA (80 µL, 0.46 mmol) were dissolved in dry DCM (4 mL). The solution was stirred 6h in rt. The reaction was monitored by TLC. Then the solvent was evaporated and amide product was purified in column chromatography. The product was taken to the next step. The amide product (25 mg, 0.054 mmol), (4-formylphenyl)boronic acid (32.5 mg, 0.217 mmol),were dissolved in ACN (0.8 mL). Acetic acid (19 µL, 0.324 mmol) and pyrrolidine (27 µL, 0.324 mmol) were added to the reaction mixture and the solution was stirred at 85 ^{o}C. TLC was monitored in every 10 mins interval. After 2 hours, the solution was evaporated and the product was purified in column chromatography to obtain BOR 2 as red solid (17.5 mg, 54.7%). δ 7.96 (d, j = 9 Hz, 2H), 7.67 (d, j = 6 Hz, 2H), 7.36 (d, j = 3 Hz, 1H), 7.32 (s, 1H), 7.06 (s, 1H), 6.45 (d, j = 3 Hz, 1H), 5.79 (s, 1H), 3.58-3.44 (m, 10H), 3.34-3.24 (m, 6H), 2.64-2.57 (m, 2H), 2.52 (s, 3H), 2.16 (d, j= 9Hz, 2H), 1.61 (m, 2H). HRMS (m/z): Calcd for $C_{30}H_{36}B_2F_2N_4O_5$ (-F) 573.2856; found 573.2841 (M-F).

Synthesis of BOR-1H

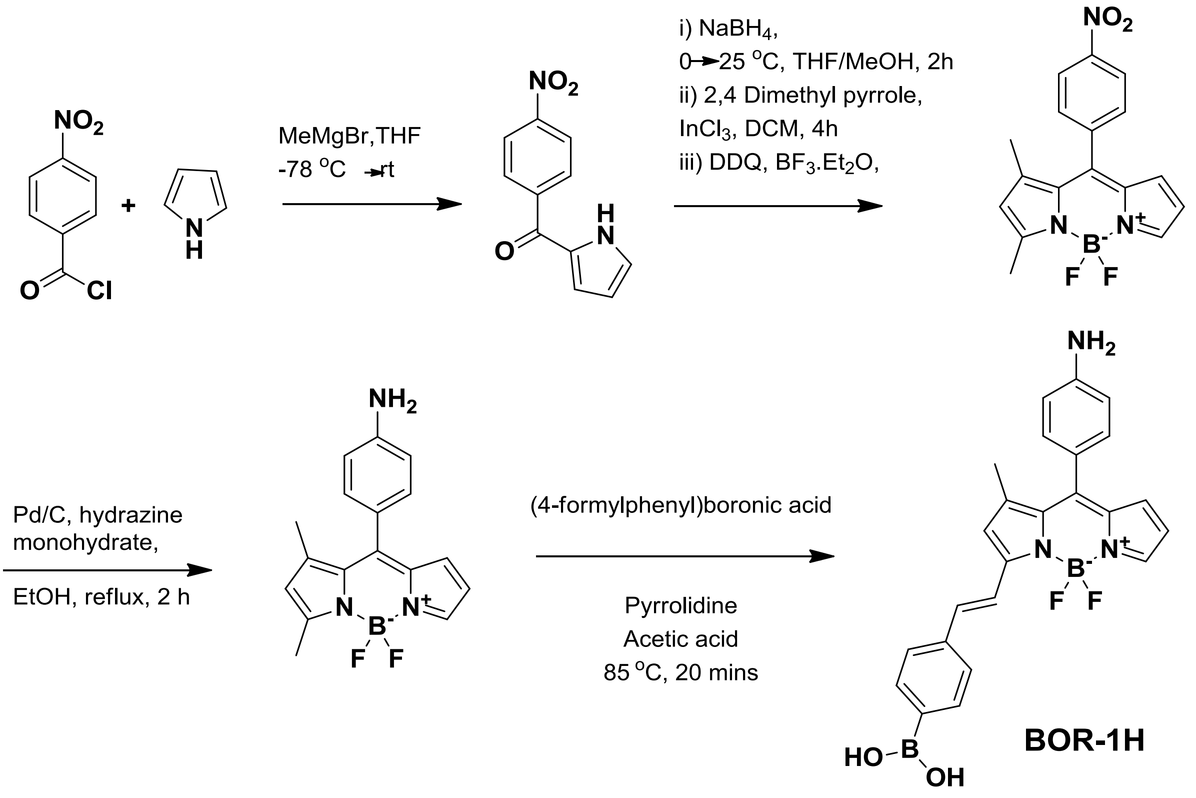

BDN-amine was prepared according to the reported procedure[8]. BDN amine (24 mg, 0.077 mmol), (4-formylphenyl)boronic acid (46.2 mg, 0.308 mmol),were dissolved in ACN (1 mL). Acetic acid (27 µL, 0.462 mmol) and pyrrolidine (38 µL, 0.462 mmol) were added to the reaction mixture and the solution was stirred at 85 ^{o}C. TLC was monitored in every 10 mins interval. After 2 hours, the solution was evaporated and the product was purified in column chromatography (MeOH:DCM = 15:85) to obtain BOR 1H as red solid (16.1 mg, 47.2%). ^{1}H NMR (d-acetone, 300 MHz): δ 7.96 (d, j= 9 Hz, 2H), 7.70-7.65 (m, 4H), 7.21 (d, j = 9 Hz, 2H), 7.06 (s, 1H), 7.04 (s, 1H), 6.86 (d, 2H), 6.63 (s, 1H), 6.49 (s, 1H), 1.83 (s, 3H). HRMS (m/z): Calcd for $C_{24}H_{21}B_2F_2N_3O_2$ 443.1788; found 442. 1706 (M-H).

Synthesis of BOR-2H

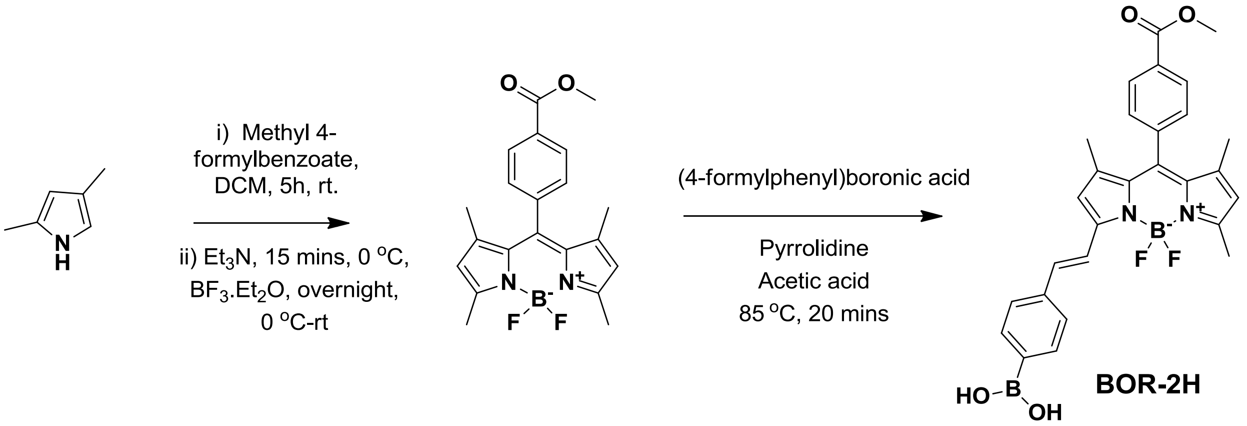

Methyl 4-formylbenzoate (4g, 20 mmol), 2,4-dimethyl-1H-pyrrole (4.56 mL, 44.3 mmol) were dissolved in dry DCM and stirred in N_2 atmosphere for 5h. The reaction mixture was cooled to 0 ^oC. Triethyl amine (10 mL, 100 mmol) was added slowly to it and stirred for 15 mins. Then, $BF_3.OEt_2$ (12.6 mL (100 mmol) was added to the reaction mixture slowly at 0 ^oC and stirred overnight at rt. Solvent was evaporated and the product was purified by column chromatography (EA:Hex = 1:10). Product was obtained as brown solid (6.65 g, 86.4%). ^1H NMR (d-acetone, 300 MHz): δ 8.22 (d, j = 9 Hz, 2H), 7.60 (d, j = 6 Hz, 2H), 6.15 (s, 2H), 3.96 (s, 3H), 2.52 (s, 6H), 1.41 (s, 6H). EI-MS (m/z): Calcd for $C_{21}H_{21}BF_2N_2O_2$ 382.16; found 381.1 (M-H). The above product (5,5-difluoro-10-(4-(methoxycarbonyl)phenyl)-1,3,7,9-tetramethyl-5H-dipyrrolo[1,2-c:2',1'-f][1,3,2]diazaborinin-4-ium-5-uide) (50 mg, 0.13 mmol), (4-formylphenyl)boronic acid (19.6 mg, 0.13 mmol) were dissloved in ACN (1 mL). To it, acetic acid (29.7 µL, 0.52 mmol) and pyrrolidine (43 µL, 0.52 mmol) were added. The reaction mixture was stirred at 85 ^oC. TLC was monitored in every 5 mins interval. After 20 mins, the reaction mixture was evaporated. The crude product was purified in column chromatography (EA:Hex = 8:2). The product obtained as dark red solid (30.8 mg, 45.9 %). ^1H NMR (d-acetone, 300 MHz): δ 8.24 (d, j = 9Hz, 2H), 7.95 (d, j = 9 Hz, 2H), 7.68-7.62 (m, 5H), 7.28 (s, 1H), 6.91 (s, 1H), 6.21 (s, 1H), 3.97 (s, 3H), 2.58 (s, 3H), 1.48 (s, 3H), 1.44 (s, 3H). HRMS (m/z): Calcd for $C_{28}H_{26}B_2F_2N_2O_4$ 514.2047; found 513.1968 (M-H).

Synthesis of AzG-1

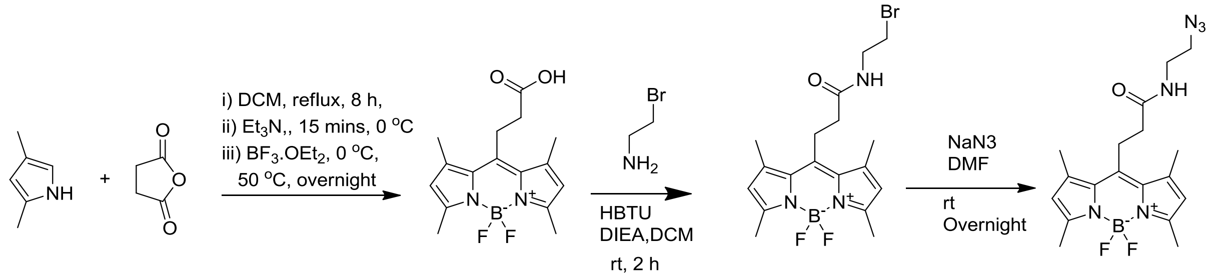

3-(4,4-Difluoro-1,3,5,7-tetramethyl-4-bora-3a,4a-diaza-s-indacene-8-yl)-propionic Acid: In a RB flask, a mixture of succinic anhydride (32 mg, 0.3 mmol) and 2,4-dimethylpyrrole (60 mg, 0.6 mmol) were dissolved in a mixture of dry CH_3CN (3 mL) was heated to reflux under nitrogen for 8 h. After the solution was cooled to 0 °C, Et_3N (180 mg, 18 mmol) was added dropwise and stirred for 15 mins. Then, $BF_3.OEt_2$ (340 mg, 24 mmol) was added at 0 °C. The reaction mixture was stirred under N_2 at 50 °C overnight. The mixture was quenched and washed with water and extracted with CH_2Cl_2, and then the organic phase was washed with brine and dried over anhydrous Na_2SO_4. Compound was purified by column chromatography on silica gel (Hexane : EA = 10:2) afforded pure compound as an orange red powder (34 mg, yield 21%). ^{1}H NMR ($CDCl_3$, 500 MHz) δ: 6.10 (s, 2H); 3.35 (t, 2H, J = 8.7Hz); 2.68 (t, 2H, J = 8.7Hz); 2.54 (s, 6H); 2.47 (s, 6H). ^{13}C NMR ($CDCl_3$, 125 MHz) δ: 175.69, 159.36, 142.77, 140.36, 131.24, 122.11, 34.85, 23.42, 16.39, 14.52. MS (m/z):319.3 ($[M-H]^+$), found 319.1.

10-(3-((2-bromoethyl)amino)-3-oxopropyl)-5,5-difluoro-1,3,7,9-tetramethyl-5H-dipyrrolo[1,2-c:2',1'-f][1,3,2]diazaborinin-4-ium-5-uide:

3-(4,4-Difluoro-1,3,5,7-tetramethyl-4-bora-3a,4a-diaza-s-indacene-8-yl)-propionic Acid (15 mg, 0.04 mmol), 2-bromoethanamine hydrochloride (19.2 mg, 0.09 mmol), HBTU (35.5 mg, 0.09 mmol) were dissolved in dry DCM in N_2 atmosphere. To the reaction mixture, DIEA (12 mg, 16.3 μL, 0.09 mmol) was added drop wise. The reaction mixture was stirred for 2 h in rt. The

mixture was washed with water and extracted with EA. Then the organic phase was washed with brine and dried over anhydrous Na_2SO_4. Compound was purified by column chromatography on silica gel (Hexane : EA = 10:7) afforded pure compound (18. 1 mg, yield 91.0 %). ^{1}H NMR (300 MHz, CDCl3) δ 6.05 (s, 2H), 5.94 (s, 1H), 3.65 (q, *J* = 5.8 Hz, 2H), 3.45 (t, *J* = 5.8 Hz, 2H), 3.37 – 3.24 (m, 2H), 2.50 (s, 6H), 2.49 – 2.44 (m, 2H), 2.43 (s, 6H). ^{13}C NMR (75 MHz, CDCl3) δ 170.64, 154.57, 144.05, 140.47, 131.23, 121.93, 41.16, 37.32, 32.00, 29.67, 23.70, 16.47, 14.44, 14.41. MS (m/z):406.10 ([M-F]), found 405.9.

Synthesis of AzG1:

10-(3-((2-bromoethyl)amino)-3-oxopropyl)-5,5-difluoro-1,3,7,9-tetramethyl-5H-dipyrrolo[1,2-c:2',1'-f][1,3,2]diazaborinin-4-ium-5-uide (18 mg, 0.04 mmol) was dissolved in dry DMF (0.2 mL) in N2 atmosphere. NaN3 (8.2 mg, 0.12 mmol) was added to the mixture and stirred overnight at room temperature. The mixture was washed with water and extracted with EA. Then the organic phase was washed with brine and dried over anhydrous Na_2SO_4. Compound was purified by column chromatography on silica gel (Hexane : EA = 10:7) afforded pure compound as brown solid (14.8 mg, yield 95.6 %). ^{1}H NMR (300 MHz, CDCl3) δ 6.06 (s, 2H), 5.81 (s, 1H), 3.43 (s, 2H), 3.42 (s, 2H), 3.38 – 3.29 (m, 2H), 2.51 (s, 6H), 2.50 – 2.45 (m, 2H), 2.43 (s, 6H). ^{13}C NMR (126 MHz, $CDCl_3$) δ 170.79, 154.59, 144.07, 140.49, 131.27, 121.93, 50.65, 39.11, 37.36, 23.64, 16.48, 14.46. HRMS (m/z):369.2005 ([M-F]), found 369.1998.

Synthesis of TPP-BCN

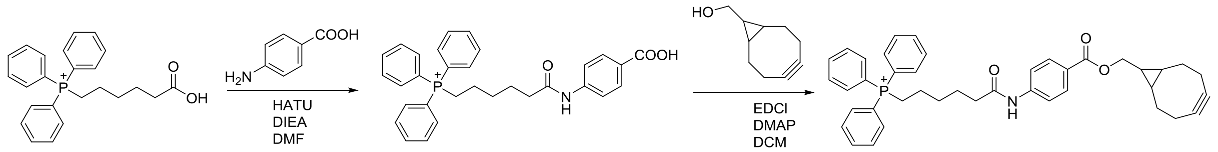

(6-((4-carboxyphenyl)amino)-6-oxohexyl)triphenylphosphonium bromide:

(5-carboxypentyl)triphenylphosphonium bromide (25 mg, 0.066 mmol), 4-aminobenzoic acid (13.7 mg, 0.1 mmol), HATU (38 mg, 0.1 mmol) were dissolved in dry DMF (2 mL). To the reaction mixture, 17 uL of DIEA was added and the reaction mixture was stirred for 2h at rt. After reaction, add EA. Solvent was washed with brine and water, organic layer was dried over anhydrous Na_2SO_4. The crude was purified by column chromatography (DCM: MeOH = 9:1). Product was obtained as white solid (12 mg, 36.6 %).^{1}H NMR (500 MHz, $CDCl_3$-MeOD) δ 8.30 (d, *J* = 6.4 Hz, 2H), 8.18 (d, *J* = 5.8 Hz, 2H), 8.10 – 7.94 (m, 15H), 3.55 (s, 2H), 2.74 (s, 2H), 2.07 (s, 4H), 1.99 (d, *J* = 4.2 Hz, 2H). ^{13}C NMR (126 MHz, $CDCl_3$-MeOD) δ 172.50, 168.04, 142.28, 134.84, 132.89, 132.81, 130.28, 130.13, 130.03, 118.47, 117.84, 117.16, 113.07, 35.90, 29.08, 23.80, 21.78, 21.59. EI-MS (m/z): Calcd for $C_{31}H_{31}NO_3P^+$ 496.57; found 496.2.

(6-((4-((bicyclo[6.1.0]non-4-yn-9-ylmethoxy)carbonyl)phenyl)amino)-6-oxohexyl)triphenylphosphonium bromide:

(6-((4-carboxyphenyl)amino)-6-oxohexyl)triphenylphosphonium bromide (6 mg, 0.012 mmol), bicyclo[6.1.0]non-4-yn-9-ylmethanol (2.7 mg, 0.018 mmol), EDCI (3.45 mg, 0.018 mmol) and DMAP (0.8 mg, 0.006 mmol) were dissolved together in the mixture of DMF and DCM (1 mL, v/v=1:9) and stirred for 12h in rt. Water (0.5 mL) was added to the reaction mixture and the organic layer was extracted in DCM. The crude was purified by column chromatography (DCM: MeOH = 9:1). Product was obtained as white solid (4 mg, 53.0 %). ^{1}H NMR (500 MHz, DMSO) δ 10.24 (s, 1H), 7.95 – 7.84 (m, 4H), 7.84 – 7.68 (m, 15H), 4.39 – 4.23 (m, 2H), 3.46 (dd, *J* = 7.5, 4.8 Hz, 2H), 2.27 – 2.08 (m, 8H), 1.67 – 1.38 (m, 8H), 0.78-0.74 (m, 3H); ^{13}C NMR (126

MHz, DMSO) δ 172.08, 165.90, 144.07, 135.37, 134.09, 134.01, 130.75, 130.66, 124.53, 119.36, 118.84, 118.68, 99.58, 99.45, 62.83, 57.76, 36.57, 29.15, 29.09, 24.62, 21.71, 21.44, 21.32, 20.33, 19.71, 17.73. EI-MS (m/z): Calcd for $C_{41}H_{43}NO_3P^+$ 628.77; found 628.3.

Synthesis of library in the training set

General procedure for the synthesis of **BDNCA** library: Purified aniline bodipy compound (BDN Library)[8] (1eq) was diluted in DCM/ACN (2/1), 10 µL of $NaHCO_3$ saturated solution was added and chloroacetyl chloride (5 eq.) was added immediately. Organic layer was washed with $NaHCO_3$ saturated solution twice. Product identity and purity was confirmed with LC-MS chromatography.

General procedure for the synthesis of **TAM** libraries: **TAM** (1/1''/2/3) acid (1 eq), N-hydroxylsuccinimide (1 eq), EDCI (1.5 eq), DMAP (0.2 eq) were dissolved in THF and stirred overnight at room temperature. The NHS-active ester formed was treated with amine (1eq) and stirred at room temperature for 6h. The crude amide was purified by preparative TLC. Product identity and purity was confirmed with LC-MS chromatography. Spectroscopic and quantum yield data were measured on a SpectraMax M2 spectrophotometer (Molecular Devices). Quantum yields were obtained by comparing the areas under the corrected emission spectrum in its respective solvents. The Supplementary Equation (1) was used to calculate quantum yield:

$$\Phi_x = \Phi_{ref}(I_x/I_{ref})(A_{ref}/A_x)(\eta_x^2/\eta_{ref}^2) \qquad (1)$$

Where Φ_{st} is the reported quantum yield of the standard, I is the integrated emission spectrum, A is the absorbance at the excitation wavelength, and ηx is the refractive index of the solvents used. The subscript x and ref denotes unknown and reference, respectively.

Supplementary References.

1. Petitjean, M. Applications of the radius-diameter diagram to the classification of topological and geometrical shapes of chemical compounds. *J. Chem. Inf. Comput. Sci.* **32**, 331–337 (1992).

2. Hall, L. H. & Kier, L. B. The Molecular Connectivity Chi Indexes and Kappa Shape Indexes in Structure-Property Relations. *Review in Computational Chemistry.* **9**, 367–422 (1991).

3. Hou, T. J.,Xia, K., Zhang, W & Xu, X. J. ADME Evaluation in Drug Discovery. 4. Prediction of Aqueous Solubility Based on Atom Contribution Approach. *J. Chem. Inf. Comput. Sci.* **44**, 266–275 (2004).

4. Wildman, S. A. & Crippen, G. M. Prediction of Physicochemical Parameters by Atomic Contributions. *J. Chem. Inf. Comput. Sci.* **39**, 868–873 (1999).

5. Ertl, P., Rohde, B. & Selzer, P. J. Fast Calculation of Molecular Polar Surface Area as a Sum of Fragment-Based Contributions and Its Application to the Prediction of Drug Transport Properties. *Med. Chem.* **43**, 3714–3717 (2000).

6. Vendrell, M. *et al.* Solid-phase synthesis of BODIPY dyes and development of an immunoglobulin fluorescent sensor. *Chem. Commun*. **47**, 8424–8426 (2011).

7. Yun, S. -W. *et al.* Neural stem cell specific fluorescent chemical probe binding to FABP7. *Proc. Natl. Acad. Sci. USA.* **109**, 10214–10217 (2012).

8. Kang, N.Y. *et al*. Visualization and isolation of Langerhans islets by fluorescent probe PiY. *Angew. Chem. Int. Ed*. **52**, 8557–8560 (2013).

9. Lee, J. S. *et al.* Synthesis of a BODIPY Library and Its Application to the Development of Live Cell Glucagon Imaging Probe. *J. Am. Chem. Soc*. **131**, 10077–10082 (2009).

10. Lee, J. S. *et al.* Accelerating fluorescent sensor discovery: unbiased screening of a diversity-oriented BODIPY library. *Chem. Commun*. **47**, 2339–2341 (2011).
